# Supplementary material for: How well do mothers recall their own and their infants’ perinatal events? A two-district study using cross-sectional stratified random sampling in Bihar, India
Source: BMJ Open. 2019 Dec 18;9(12):e031289. doi: 10.1136/bmjopen-2019-031289 (PMC6937048; doi:10.1136/bmjopen-2019-031289)
Supplement: Supplementary data [file bmjopen-2019-031289supp001.pdf]

Table S1. Subject domain and indicators labels		
Domain[a]	Indicator	Indicator text
1	1	Proportion of mothers of infants (0-2/0-5) months who were registered during their last pregnancy
1	2	Proportion of mothers of infants (0-2/0-5) months whose last pregnancy was registered in the first three months of pregnancy
1	3	Proportion of mothers of infants (0-2/0-5) months attended at least one ANC visit during their last pregnancy
1	4	Proportion of mothers of infants (0-2/0-5) months attended 3 or more ANC visits during their last pregnancy
1	5	Proportion of mothers of infants (0-2/0-5) months attended 4 or more ANC visits during their last pregnancy
1	6	Proportion of mothers of infants (0-2/0-5) months who attended at least one ANC visit where BP was checked during her last pregnancy
1	7	Proportion of mothers of infants (0-2/0-5) months who attended at least one ANC where at least one sonography was performed during her last pregnancy
1	8	Proportion of mothers of infants (0-2/0-5) months who attended at least one ANC where at least one abdominal examination was performed during her last pregnancy
1	9	Proportion of mothers of infants (0-2/0-5) months who attended at least one ANC where at least one urine test was performed during her last pregnancy
1	10	Proportion of mothers of infants (0-2/0-5) months who attended at least one ANC where at least one blood test was performed during her last pregnancy
1	40	Proportion of mothers of infants (0-2/0-5) months who have used an ambulance to reach an institution for last delivery
2	11	Proportion of mothers of infants (0-2/0-5) months who were protected against tetanus in their last pregnancy (Neonatal TT)
2	12	Proportion of mothers of infants (0-2/0-5) months who received two or more doses of TT injection in their last pregnancy
2	13	Proportion of mothers of infants (0-2/0-5) months who received IFA for 100 days or more during their pregnancy
2	14	Proportion of mothers of infants (0-2/0-5) months consumed IFA for 100 days or more in their pregnancy
3	15	Proportion of mothers (home + institutional delivery) of infants (0-2/0-5) months who planned transportation to health facility in their last pregnancy
3	16	Proportion of mothers (home + institutional delivery) of infants (0-2/0-5) months who have identified anybody who would donate blood in the case of emergency in their last pregnancy

3	17	Proportion of mothers (home + institutional delivery) of infants (0-2/0-5) months who have identified persons who would take care of the baby immediately after birth
3	18	Proportion of mothers (home + institutional delivery) of infants (0-2/0-5) months who have arranged new blade & thread for their last delivery
3	19	Proportion of mothers who planned for institutional delivery of infants (0-2/0-5) months who arranged new blade & thread for their delivery
3	20	Proportion of mothers (home + institutional delivery) of infants (0-2/0-5) months who have arranged clean cloth for mothers and baby
3	21	Proportion of mothers who planned institutional delivery of infants (0-2/0-5) months who arranged clean cloth for mothers and baby
3	22	Proportion of mothers of infants (0-2/0-5) months who have saved money for the delivery
3	23	Proportion of mothers who planned for institutional delivery of infants (0-2/0-5) months identified person to accompany her during the delivery
4	24	Proportion of mothers of infants (0-2/0-5) months who were visited by ASHA at least once during their last pregnancy
4	25	Proportion of mothers of infants (0-2/0-5) months who were visited by AWW at least once during their last pregnancy
4	26	Proportion of mothers of infants (0-2/0-5) months who were visited by FLWs at least once during their last pregnancy
4	27	Proportion of mothers of infants (0-2/0-5 months) visited by ASHAs at least once during their last trimester of pregnancy
4	28	Proportion of mothers of infants (0-2/0-5) months who were visited by AWW at least once during their last pregnancy
4	29	Proportion of mothers of infants (0-2/0-5) months who were visited by any FLW in the last trimester during their last pregnancy
4	30	Proportion of mothers of infants (0-2/0-5) months who were visited 2 or more times by any FLW in the last trimester during their last pregnancy
4	31	Proportion of mothers of infants (0-2/0-5) months who were visited home by ASHA within 24 hours of last delivery
4	32	Proportion of mothers of infants (0-2/0-5) months who were visited home by AWW within 24 hours of last delivery
4	33	Proportion of mothers of infants (0-2/0-5) months who were visited home by any FLW within 24 hours of last delivery
4	34	Proportion of mothers of infants (0-2/0-5) months who were visited home by any AWW within the first week of last delivery

4	35	Proportion of mothers of infants (0-2/0-5) months who were visited home by any FLW within first week of last delivery
5	37	Proportion of mothers of infants (0-2/0-5) months whose last child was delivered at a health facility (private or public facility)
5	38	Proportion of mothers of infants (0-2/0-5) months whose last child was delivered at public facility
5	39	Proportion of mothers (home delivery) of infants (0-2/0-5) months who had home delivery attended by skilled birth attendant (SBA)
5	39.5	Proportion of mothers (home + institutional delivery) of infants (0-2/0-5) months who had home delivery attended by skill birth attendant (SBA)
6	42	Proportion of infants (home + institutional delivery) aged (0-2/0-5) months with nothing applied to the umbilical cord after cutting and tying in their last delivery
6	43	Proportion of mothers (home + institutional delivery) of infants (0-2/0-5) months who have delivered baby practiced skin to skin care (STSC) immediately after birth
6	44	Proportion of mothers with institutional delivery of infants (0-2/0-5) months who continued skin to skin care (STSC) at home
6	45	Proportion of mothers with home delivery of infants (0-2/0-5) months who continued skin to skin care (STSC) later
6	46	Proportion of mothers of infants (0-2/0-5) months who were breastfed within one hour of birth
6	47	Proportion of infants aged (0-2/0-5) months who have received delayed bath (between 48 hours and before 7 days after birth) (Public facility/Private facility/Home)
6	48	Proportion of infants aged (0-2/0-5) months who were weighed after birth (Public facility/Private facility/Home)
6	49	Proportion of infants aged (0-2/0-5) months who were delivered at HF received dry cord care
6	50	Proportion of infants aged (0-2/0-5) months who were delivered at HF continued with dry cord care
6	51	Proportion of infants aged (0-2/0-5) months who were delivered at home continued with dry cord care
7	52	Proportion of infants (0-2/0-5) months who were breast-fed in the past 24 hours (Exclusively Breast-fed)

a. 1 Antenatal care, 2 Maternal health, 3 Birth preparedness, 4 Front Line Worker Support, 6 Place of birth & attendant, 7 Exclusive Breastfeeding
