# Supplementary material for: How well do mothers recall their own and their infants’ perinatal events? A two-district study using cross-sectional stratified random sampling in Bihar, India
Source: BMJ Open. 2019 Dec 18;9(12):e031289. doi: 10.1136/bmjopen-2019-031289 (PMC6937048; doi:10.1136/bmjopen-2019-031289)
Supplement: Supplementary data [file bmjopen-2019-031289supp002.pdf]

Table S2. Indicators from two samples in Aurangabad and Gopalganj of mothers of infants aged 0-2 months compared to 0-5 months: Estimates, Cochran-Mantel-Haenszel Chi-squares, and probabilities: Bihar 2015

			Unweighted				Weighted			
Domain[a]	Indicator[b]	District[c]	Percent 0-2	Percent 0-5	Chi-square	Probability	Percent 0-2	Percent 0-5	Chi-square	Probability
1	1	1	86.6	79.9	3.6775	0.0552	85.2	77.6	4.3758	0.0365
1	1	2	85.3	85.3	0.0000	1.0000	85.0	85.4	0.0187	0.8911
1	2	1	44	40.7	0.4996	0.4797	43.0	38.3	1.0147	0.3138
1	2	2	47.7	47.0	0.0304	0.8616	47.5	48.2	0.0212	0.8843
1	3	1	84.7	80.9	1.2398	0.2655	83.8	80.4	0.9592	0.3274
1	3	2	81.2	78.6	0.5985	0.4391	80.6	77.8	0.6424	0.4229
1	4	1	60.3	63.6	0.4931	0.4826	61.1	63.1	0.1792	0.6720
1	4	2	59.4	63.2	0.8252	0.3637	57.4	61.0	0.7211	0.3958
1	5	1	42.1	45.0	0.3491	0.5546	43.2	45.7	0.2561	0.6128
1	5	2	44.7	47.7	0.4866	0.4854	43.4	46.9	0.6302	0.4273
1	6	1	59.8	59.3	0.0107	0.9177	58.9	60.1	0.0644	0.7996
1	6	2	67.7	63.5	1.0487	0.3058	66.3	63.3	0.5176	0.4719
1	7	1	42.6	39.2	0.5043	0.4776	42.0	39.5	0.2824	0.5952
1	7	2	70.3	73.3	0.6025	0.4376	69.2	72.6	0.7537	0.3853
1	8	1	44.0	45.9	0.1685	0.6815	42.7	44.8	0.2028	0.6525
1	8	2	53.4	54.1	0.0322	0.8576	52.4	54.5	0.2438	0.6215
1	9	1	45.9	42.6	0.5004	0.4793	45.8	41.7	0.7523	0.3857
1	9	2	42.9	43.6	0.0321	0.8579	42.8	43.5	0.0231	0.8792
1	10	1	67.0	59.8	2.6555	0.1032	67.2	59.6	2.9924	0.0837
1	10	2	55.3	58.3	0.5191	0.4712	54.8	58.5	0.7674	0.3810
1	40	1	11.5	17.7	3.4797	0.0621	10.6	16.0	2.8329	0.0924
1	40	2	8.6	10.2	0.3686	0.5438	8.5	9.5	0.1374	0.7109
2	11	1	98.6	98.1	0.1451	0.7033	98.5	98.1	0.0844	0.7715
2	11	2	97.0	96.6	0.0614	0.8044	96.9	97.1	0.0131	0.9089
2	12	1	68.4	66.5	0.1763	0.6746	69.3	67.5	0.1626	0.6868
2	12	2	79.7	79.3	0.0116	0.9141	81.9	80.4	0.2143	0.6434
2	13	1	3.8	2.4	0.7384	0.3902	4.0	2.6	0.6021	0.4378
2	13	2	3.4	5.6	1.5745	0.2096	3.0	5.1	1.6016	0.2057

Table S2. Indicators from mothers two samples in Aurangabad and Gopalganj of mothers of infants aged 0-2 months compared to 0-5 months: Estimates, Cochran-Mantel-Haenszel Chi-squares, and probabilities: Bihar 2015 - Continued

			Unweighted				Weighted			
Domain[a]	Indicator[b]	District[c]	Percent 0-2	Percent 0-5	Chi-square	Probability	Percent 0-2	Percent 0-5	Chi-square	Probability
2	14	1	1.4	1.9	0.1474	0.7010	1.6	2.0	0.1025	0.7489
2	14	2	1.9	3.8	1.7161	0.1902	1.7	3.6	1.8535	0.1734
3	15	1	26.3	34.0	3.1510	0.0759	25.9	33.3	2.9594	0.0854
3	15	2	47.0	56.4	4.9192	0.0266	45.7	56.0	5.8262	0.0158
3	16	1	4.3	3.8	0.0639	0.8004	4.3	3.6	0.1410	0.7073
3	16	2	10.2	13.9	1.8263	0.1766	9.7	14.3	2.8157	0.0933
3	17	1	48.8	56.5	2.5852	0.1079	48.5	55.6	2.2155	0.1366
3	17	2	53.4	62.8	4.9914	0.0255	51.8	62.6	6.5790	0.0103
3	18	1	24.9	22.5	0.3601	0.5485	26.1	23.4	0.4531	0.5009
3	18	2	39.5	45.1	2.2194	0.1363	38.2	43.7	2.1362	0.1439
3	19	1	22.6	14.4	3.4821	0.0620	23.5	14.5	4.0983	0.0429
3	19	2	45.2	40.7	1.1508	0.2834	43.4	39.4	0.6771	0.4106
3	20	1	40.7	37.8	0.4565	0.4993	44.1	39.5	1.1927	0.2748
3	20	2	51.9	57.1	1.8538	0.1733	50.0	55.9	2.3539	0.1250
3	21	1	40.1	30.4	3.6953	0.0546	43.6	31.2	6.0749	0.0137
3	21	2	58.1	52.8	1.7277	0.1887	55.6	51.6	0.8220	0.3646
3	22	1	65.1	59.3	1.5561	0.2122	65.5	59.4	1.7643	0.1841
3	22	2	62.4	68.4	2.2445	0.1341	60.2	68.0	3.6903	0.0547
3	23	1	65.0	49.0	8.3301	0.0039	62.5	47.0	7.8078	0.0052
3	23	2	68.8	63.0	1.8993	0.1682	68.0	62.5	1.5725	0.2098
4	24	1	61.2	75.1	9.2638	0.0023	62.2	75.2	8.2048	0.0042
4	24	2	65.8	74.4	4.8055	0.0284	63.5	73.0	5.6444	0.0175
4	25	1	11.5	12.9	0.2096	0.6471	11.1	12.5	0.2064	0.6496
4	25	2	20.7	18.4	0.4319	0.5111	19.3	19.1	0.0019	0.9656
4	26	1	62.7	76.6	9.4840	0.0021	63.5	76.7	8.6936	0.0032
4	26	2	68.4	75.9	3.8007	0.0512	66.9	74.3	3.5169	0.0607
4	27	1	42.1	47.8	1.4400	0.2301	42.3	47.6	1.2194	0.2695
4	27	2	54.5	62.4	3.4903	0.0617	52.6	61.1	4.0230	0.0449

Table S2. Indicators from mothers two samples in Aurangabad and Gopalganj of mothers of infants aged 0-2 months compared to 0-5 months: Estimates, Cochran-Mantel-Haenszel Chi-squares, and probabilities: Bihar 2015 - Continued										
			Unweighted				Weighted			
Domain[a]	Indicator[b]	District[c]	Percent 0-2	Percent 0-5	Chi-square	Probability	Percent 0-2	Percent 0-5	Chi-square	Probability
4	28	1	8.6	8.1	0.0323	0.8575	8.2	7.6	0.0561	0.8127
4	28	2	16.2	13.9	0.5345	0.4647	15.4	13.8	0.2732	0.6012
4	29	1	43.1	48.8	1.4361	0.2308	43.3	48.5	1.1846	0.2764
4	29	2	57.5	64.7	2.9183	0.0876	55.9	63.5	3.2352	0.0721
4	30	1	29.7	35.4	1.6374	0.2007	28.7	34.2	1.5497	0.2132
4	30	2	48.1	53	1.2686	0.2600	46.9	51.4	1.0908	0.2963
4	31	1	30.1	45.9	10.9581	0.0000	29.9	44.9	9.9713	0.0016
4	31	2	47.4	53.0	1.6849	0.1943	44.5	52.7	3.6136	0.0573
4	32	1	5.3	3.8	0.4948	0.4818	5.3	3.6	0.7401	0.3896
4	32	2	9.8	9.0	0.0897	0.7646	9.1	9.9	0.0974	0.7550
4	33	1	32.5	47.8	10.1305	0.0015	32.2	46.7	9.0939	0.0026
4	33	2	50.8	56.4	1.7133	0.1906	48.2	56.2	3.5264	0.0604
4	34	1	6.2	10.0	2.0808	0.1492	6.2	9.9	1.9617	0.1613
4	34	2	13.5	9.0	2.7721	0.0959	14.4	8.9	3.9431	0.0471
4	35	1	45.0	60.3	9.7398	0.0018	44.5	59.3	9.0930	0.0026
4	35	2	64.7	69.5	1.4491	0.2287	62.5	69.0	2.5201	0.1124
5	37	1	73.2	76.6	0.6496	0.4203	72.9	74.9	0.2182	0.6404
5	37	2	80.1	86.1	3.4220	0.0643	78.9	85.5	3.9858	0.0459
5	38	1	55.5	56.5	0.0389	0.8436	54.7	55.3	0.0117	0.9139
5	38	2	52.3	61.3	4.3834	0.0363	51.2	61.6	5.8090	0.0159
5	39	1	5.7	0	3.3034	0.0691	5.4	0	3.6637	0.0556
5	39	2	10.2	8.3	0.2393	0.6247	9.1	6.6	0.3451	0.5569
5	39.5	1	1.4	0	0.0003	0.0833	1.4	0	2.8978	0.0887
5	39.5	2	1.9	1.1	0.5139	0.4735	1.8	0.9	0.7629	0.3824
6	42	1	42.6	47.4	1.0165	0.3134	43.8	47.6	0.6553	0.4182
6	42	2	41.4	46.2	1.3134	0.2518	41.8	46.3	1.1228	0.2893
6	43	1	24.9	29.7	1.3262	0.2495	25.6	30.4	1.3008	0.2541
6	43	2	27.1	29.7	0.4968	0.4809	26.9	31.2	1.2586	0.2619

Table S2. Indicators from mothers two samples in Aurangabad and Gopalganj of mothers of infants aged 0-2 months compared to 0-5 months: Estimates, Cochran-Mantel-Haenszel Chi-squares, and probabilities: Bihar 2015 - Continued

			Unweighted				Weighted			
Domain[a]	Indicator[b]	District[c]	Percent 0-2	Percent 0-5	Chi-square	Probability	Percent 0-2	Percent 0-5	Chi-square	Probability
6	44	1	9.8	12.5	1.2833	0.2573	12.2	13.0	0.3958	0.5293
6	44	2	21.1	20.1	0.0586	0.8087	21.6	18.7	0.5178	0.4718
6	45	1	7.5	10.2	0.6321	0.4266	7.8	11.1	0.8502	0.3565
6	45	2	8.2	5.6	0.4009	0.5266	5.2	8.3	0.1670	0.6828
6	46	1	55.5	58.9	0.4904	0.4837	56.6	59.2	0.2960	0.5864
6	46	2	62.8	68.0	1.6764	0.1954	61.7	66.3	1.2736	0.2591
6	47	1	34.0	32.1	0.1768	0.6741	35.9	32.8	0.4369	0.5086
6	47	2	67.3	66.5	0.0344	0.8528	66.6	68.3	0.1745	0.6762
6	48	1	65.6	67.5	0.1782	0.6729	64.9	66.6	0.1312	0.7172
6	48	2	71.1	77.8	3.2208	0.0727	70.7	78.2	3.9664	0.0464
6	49	1	62.7	59.4	0.1537	0.6950	64.5	61.2	0.1691	0.6809
6	49	2	68.1	67.2	0.0166	0.8974	67.3	66.8	0.0011	0.9735
6	50	1	49.0	55.0	1.2527	0.2630	48.4	54.7	1.3188	0.2508
6	50	2	56.8	55.9	0.0964	0.7562	56.1	55.5	0.0615	0.8042
6	51	1	81.1	44.9	14.9736	0.0000	78.0	45.4	11.7395	0.0000
6	51	2	63.3	38.9	4.0901	0.0431	63.7	41.1	3.4654	0.0627
7	52	1	70.3	59.8	5.1727	0.0229	69.2	59.7	4.1723	0.0411
7	52	2	82.3	67.3	16.2014	0.0000	82.1	68.4	13.3711	0.0000

a . 1 Antenatal care, 2 Maternal health, 3 Birth preparedness, 4 FLW Support, 5 Place of birth & attendant, 6 Neonatal Health, 7 Exclusive breastfeeding
b . For text see Table S1
c. 1 Aurangabad, 2 Gopalganj
