# Supplementary material for: How well do mothers recall their own and their infants’ perinatal events? A two-district study using cross-sectional stratified random sampling in Bihar, India
Source: BMJ Open. 2019 Dec 18;9(12):e031289. doi: 10.1136/bmjopen-2019-031289 (PMC6937048; doi:10.1136/bmjopen-2019-031289)
Supplement: Supplementary data [file bmjopen-2019-031289supp003.pdf]

Table S3. Weighted indicators and confidence intervals from two samples in two districts of Bihar										
Indicator[a]	District[b]	0-2 Month Sample			0-5 Month Sample			Difference		
		Point estimate	Confidence interval[c]		Point estimate	Confidence interval[c]		Point estimate	Confidence interval	
			Lower	Upper		Lower	Upper		Lower	Upper
1	1	85.2	80.2	90.2	77.6	72.6	82.6	7.6	0.1	15.2
1	2	85.0	80.5	89.6	85.4	80.9	90.0	-0.4	-6.7	5.9
2	1	43.0	36.3	49.8	38.3	31.5	45.0	4.7	-4.5	14.0
2	2	47.5	41.5	53.6	48.2	42.1	54.2	-0.6	-9.5	8.2
3	1	83.8	78.8	88.8	80.4	75.4	85.4	3.4	-3.6	10.4
3	2	80.6	75.7	85.5	77.8	72.9	82.7	2.8	-4.5	10.0
4	1	61.1	54.4	67.8	63.1	56.4	69.9	-2.0	-11.4	7.3
4	2	57.4	51.2	63.6	61.0	54.8	67.2	-3.5	-12.3	5.2
5	1	43.2	36.4	50.1	45.7	38.9	52.6	-2.5	-12.2	7.2
5	2	43.4	37.1	49.8	46.9	40.5	53.2	-3.4	-12.4	5.6
6	1	58.9	52.3	65.6	60.1	53.5	66.7	-1.2	-10.4	8.0
6	2	66.3	60.2	72.4	63.3	57.2	69.4	2.9	-5.7	11.6
7	1	42.0	35.3	48.8	39.5	32.7	46.3	2.5	-7.0	12.1
7	2	69.2	63.2	75.2	72.6	66.6	78.6	-3.4	-11.7	4.9
8	1	42.7	36.1	49.3	44.8	38.2	51.4	-2.1	-11.4	7.2
8	2	52.4	46.2	58.7	54.5	48.2	60.8	-2.1	-10.9	6.7
9	1	45.8	39.0	52.6	41.7	34.9	48.4	4.1	-5.4	13.6
9	2	42.8	36.6	49.1	43.5	37.3	49.7	-0.6	-9.5	8.2
10	1	67.2	60.9	73.6	59.6	53.2	65.9	7.6	-1.2	16.5
10	2	54.8	48.5	61.1	58.5	52.2	64.8	-3.7	-12.5	5.2
11	1	98.5	96.7	100.2	98.1	96.4	99.9	0.4	-2.2	2.9
11	2	96.9	94.8	99.1	97.1	94.9	99.3	-0.2	-3.0	2.7
12	1	69.3	63.0	75.7	67.5	61.2	73.9	1.8	-7.2	10.8
12	2	81.9	77.3	86.6	80.4	75.7	85.0	1.6	-5.2	8.4
13	1	4.0	1.2	6.7	2.6	-0.1	5.4	1.3	-2.2	4.9
13	2	3.0	1.0	5.0	5.1	3.1	7.1	-2.2	-5.5	1.2
14	1	1.6	-0.2	3.4	2.0	0.2	3.8	-0.4	-3.1	2.3
14	2	1.7	0.2	3.2	3.6	2.1	5.1	-1.9	-4.7	0.9
15	1	25.9	20.1	31.6	33.3	27.5	39.0	-7.4	-15.9	1.1
15	2	45.7	39.6	51.8	56.0	49.9	62.1	-10.3	-19.0	-1.5
16	1	4.3	1.5	7.0	3.6	0.8	6.3	0.7	-3.0	4.4
16	2	9.7	6.1	13.3	14.3	10.8	17.9	-4.7	-10.5	1.1
17	1	48.5	41.8	55.2	55.6	48.9	62.3	-7.1	-16.5	2.4
17	2	51.8	45.6	58.0	62.6	56.4	68.8	-10.8	-19.6	-2.1
18	1	26.1	20.2	32.1	23.4	17.5	29.4	2.7	-5.5	10.9
18	2	38.2	33.0	43.3	43.7	38.5	48.8	-5.5	-13.2	2.2
19	1	23.5	16.4	30.7	14.5	7.3	21.7	9.1	0.4	17.7
19	2	43.4	37.3	49.5	39.4	33.3	45.5	4.0	-4.6	12.6
20	1	44.1	38.3	50.0	39.5	33.7	45.4	4.6	-3.7	12.9
20	2	50.0	44.7	55.4	55.9	50.5	61.3	-5.9	-13.7	2.0
21	1	43.6	36.7	50.5	31.2	24.3	38.1	12.4	3.3	21.5
21	2	55.6	49.5	61.7	51.6	45.5	57.7	4.0	-4.6	12.6
22	1	65.5	59.0	72.0	59.4	52.9	65.9	6.1	-3.0	15.2
22	2	60.2	54.0	66.3	68.0	61.8	74.2	-7.8	-16.4	0.7
23	1	62.5	54.9	70.2	47.0	39.3	54.7	15.5	5.4	25.7
23	2	68.0	61.2	74.8	62.5	55.7	69.3	5.5	-3.8	14.8

24	1	62.2	55.5	68.9	75.2	68.5	81.9	-13.0	-22.0	-4.1
24	2	63.5	57.4	69.7	73.0	66.9	79.2	-9.5	-17.9	-1.1
25	1	11.1	7.0	15.1	12.5	8.4	16.5	-1.4	-7.5	4.7
25	2	19.3	14.4	24.2	19.1	14.3	24.0	0.1	-6.9	7.2
26	1	63.5	56.8	70.1	76.7	70.1	83.4	-13.2	-22.1	-4.4
26	2	66.9	60.9	73.0	74.3	68.3	80.3	-7.4	-15.7	1.0
27	1	42.3	35.6	49.1	47.6	40.8	54.4	-5.3	-14.8	4.3
27	2	52.6	46.3	58.9	61.1	54.8	67.4	-8.5	-17.2	0.3
28	1	8.2	4.6	11.8	7.6	4.0	11.2	0.6	-4.4	5.7
28	2	15.4	11.0	19.8	13.8	9.4	18.3	1.6	-4.7	7.8
29	1	43.3	36.5	50.1	48.5	41.7	55.3	-5.2	-14.8	4.3
29	2	55.9	49.7	62.2	63.5	57.2	69.8	-7.5	-16.3	1.2
30	1	28.7	22.6	34.7	34.2	28.1	40.2	-5.5	-14.3	3.2
30	2	46.9	40.5	53.3	51.4	45.0	57.8	-4.5	-13.5	4.5
31	1	29.9	23.5	36.3	44.9	38.5	51.3	-15.0	-24.4	-5.6
31	2	44.5	38.2	50.8	52.7	46.5	59.0	-8.2	-17.2	0.7
32	1	5.3	2.2	8.5	3.6	0.4	6.7	1.7	-2.3	5.8
32	2	9.1	5.7	12.6	9.9	6.4	13.4	-0.8	-6.0	4.4
33	1	32.2	25.8	38.7	46.7	40.2	53.2	-14.5	-23.9	-5.0
33	2	48.2	41.9	54.5	56.2	49.9	62.5	-8.1	-16.9	0.8
34	1	6.2	2.8	9.6	9.9	6.5	13.3	-3.7	-8.9	1.5
34	2	14.4	9.8	19.0	8.9	4.4	13.5	5.5	-0.3	11.3
35	1	44.5	37.7	51.4	59.3	52.5	66.2	-14.8	-24.4	-5.1
35	2	62.5	56.4	68.7	69.0	62.9	75.2	-6.5	-15.1	2.1
37	1	72.9	66.7	79.1	74.9	68.7	81.0	-2.0	-10.5	6.5
37	2	78.9	73.5	84.2	85.5	80.2	90.8	-6.6	-13.7	0.4
38	1	54.7	47.8	61.7	55.3	48.4	62.2	-0.5	-10.3	9.2
38	2	51.2	44.7	57.6	61.6	55.2	68.0	-10.4	-19.4	-1.5
39	1	5.4	-0.9	11.8	0.0	-6.4	6.4	5.4	-0.9	11.8
39	2	9.1	1.3	16.9	6.6	-1.2	14.4	2.5	-9.1	14.1
39.5	1	1.4	-0.2	3.0	0.0	-1.6	1.6	1.4	-0.2	3.0
39.5	2	1.8	0.2	3.4	0.9	-0.6	2.5	0.9	-1.0	2.8
40	1	10.6	6.5	14.7	16.0	12.0	20.1	-5.4	-11.7	0.8
40	2	8.5	4.9	12.2	9.5	5.8	13.1	-0.9	-5.9	4.1
42	1	43.8	36.9	50.6	47.6	40.7	54.5	-3.8	-13.3	5.6
42	2	41.8	35.6	48.1	46.3	40.1	52.6	-4.5	-13.4	4.4
43	1	25.6	19.8	31.5	30.4	24.6	36.2	-4.8	-13.3	3.7
43	2	26.9	21.5	32.4	31.2	25.7	36.6	-4.2	-12.0	3.5
44	1	12.2	7.3	17.1	13.0	8.1	17.9	-0.8	-7.7	6.1
44	2	21.6	16.3	26.8	18.7	13.4	24.0	2.8	-4.4	10.1
45	1	7.8	0.4	15.2	11.1	3.7	18.5	-3.3	-16.2	9.6
45	2	5.2	0.3	10.1	8.3	3.4	13.2	-3.1	-17.4	11.2
46	1	56.6	49.9	63.2	59.2	52.5	65.8	-2.6	-12.2	7.0
46	2	61.7	55.5	67.9	66.3	60.1	72.6	-4.6	-13.3	4.1
47	1	35.9	29.3	42.5	32.8	26.2	39.5	3.0	-6.2	12.3
47	2	66.6	60.6	72.7	68.3	62.3	74.4	-1.7	-10.1	6.7
48	1	64.9	58.5	71.4	66.6	60.1	73.0	-1.6	-10.8	7.5
48	2	70.7	64.9	76.5	78.2	72.4	84.0	-7.5	-15.2	0.2
49	1	64.5	57.1	71.8	61.2	53.9	68.5	3.3	-6.7	13.3
49	2	67.3	60.6	73.9	66.8	60.1	73.4	0.5	-8.9	9.9
50	1	48.4	40.3	56.4	54.7	46.7	62.8	-6.4	-17.4	4.7
50	2	56.1	49.0	63.2	55.5	48.4	62.6	0.7	-9.2	10.5

51	1	78.0	65.9	90.2	45.4	33.3	57.6	32.6	13.0	52.2
51	2	63.7	48.8	78.5	41.1	26.2	56.0	22.5	-2.2	47.2
52	1	69.2	62.7	75.6	59.7	53.3	66.1	9.5	0.2	18.8
52	2	82.1	77.1	87.0	68.4	63.5	73.4	13.6	6.0	21.2

a. For text see Table S1
b. 1 Aurangabad, 2 Gopalganj
c. Estimated with Stata command svy
