# Supplementary material for: How well do mothers recall their own and their infants’ perinatal events? A two-district study using cross-sectional stratified random sampling in Bihar, India
Source: BMJ Open. 2019 Dec 18;9(12):e031289. doi: 10.1136/bmjopen-2019-031289 (PMC6937048; doi:10.1136/bmjopen-2019-031289)
Supplement: Supplementary data [file bmjopen-2019-031289supp004.pdf]

Supplementary Text for Tables S4a through S6

We make four comparisons to inform an evaluation of these data. The first compares the 0-5 month sample to the 0-2 month sample and is presented in Table 2 in the main text and Tables S2-3. In a second comparison, indicators estimated for the 0-2 month old **subsample** are compared to the 3-5 month old **subsample** to assess the internal consistency of estimates for the entire 0-5 sample (Table 4a-b). In the third comparison, the 0-2 **subsample** of the 0-5 sample is compared to the 0-2 month **sample** to assess the sampling variability for this age group (Tables S5a-b). Finally, in the fourth comparison, the 3-5 **subsample** of the 0-5 sample is compared to the 3-5 month **sample** (Table S6). This last comparison is limited because the 3-5 month sample collected limited data; there are only 3 indicators common to the two samples.

Table S4a has the complete results for the second comparison with point estimates for the two **subsamples** 0-2 and 3-5 of the 0-5 sample, differences and estimated confidence intervals for the estimates and differences of 104 comparisons. Fourteen indicators for which the estimated confidence interval of the difference does not include zero are listed in Table S4b. The table has 2 panels. In the top panel are 6 indicators for which a difference is also reported in Table S2. Table S2 compares the 0-2 month sample and the 0-5 month sample. For each of these indicators the reported difference is in the same direction. Two of these indicators are indicator 52, exclusive infant breast feeding, in the 2 provinces. These differences are not surprising as it is common for mothers to introduce supplemental foods as infants age. The authors speculate in their paper that the timing of the monsoon may have reduced some of the indicators for the younger infants. Of the remaining four indicators of the top panel, three of the indicator differences are negative, lower for the younger infants and might plausibly be related to a monsoon. In the bottom panel of Table S4b are 8 indicators where zero is not in the confidence interval of the difference between subsamples, which suggests difference, but the results between two full-samples in Tables S2 and S3 suggest there is no meaningful difference, except that one of them, indicator 37 in Gopalganj; it does show a difference in Table S2. Of these 8, the difference between samples is in the same direction five times and in a different direction three times. For the 5 indicators where the differences are in the same direction, the absolute value of the differences between 0-2 subsample and the 3-5 subsample are larger than the absolute values of the differences between the 0-2 month sample and the 3-5 subsample. For these 5 indicators the 0-2 month **sample** is more like the 3-5 **subsample** than the 0-2 **subsample**. For the 3 indicators where the differences are in the different directions the 0-2 **subsample** closely resembles the 0-2 month **sample**; two of the estimates are different by less than 1 percent and the third by 2.4 percent.

A third comparison is in Tables S5a-b. In Table S5a the 0-2 **subsample** is compared to the 0-2 month **sample**. The expectation here would be that there are no differences because these two samples are designed to represent the same population. However, this expectation is not met; there are 10 differences where the confidence interval for the difference does not contain 0. These differences are listed in Table S5b. Nine of these 10 indicators are also among the differences in Table S3 and Table 3 in the paper. For 3 of these 9 differences the 3-5 subsample is closer to the 0-2 month sample than it is the 0-2 subsample (indicators 15, 17-2, 51); and for 3 indicators the 3-5 subsample is further from the 0-2 month sample than the 0-2 subsample (indicators 31, 33, 35). For 3 indicators the 0-2 and 3-5 subsample indicator values are nearly equal (indicators 23, 24, 26). For the final indicator in this list (indicator 17-1, the indicator for which zero is in the confidence interval of the difference between the 0-2

month sample and the 0-5 sample in Table S3) the difference between the indicator values for the 0-2 and 3-5 subsamples and the 0-2 month sample are about equal in magnitude but have opposite signs.

Results from the fourth comparison, comparing the 3-5 **subsample** of the 0-5 **sample** to the 3-5 month sample are limited, as noted above. There are only 3 indicators in the 2 districts – six comparisons. Zero is within the confidence interval of 5 of the differences (Table S6).

We note in the paper that in making this number of comparisons one must expect that some will be large enough to be considered meaningful by chance alone. In the above analysis there are 3 comparisons of 51 indicators in 2 provinces producing 16, 14 and 10 differences and a fourth comparison with 1 difference in 6. In their paper the authors find that some of these differences are readily understood and others may be interpreted effects of monsoon rains. Post hoc interpretation is risky here; many of these differences may be noise in the data. There is evidence in the third comparison to support this position with 3 differences moving the 0-5 **sample** closer to the 0-2 month **sample**, 3 moving it away, and 4 not moving it one way or the other. Furthermore, 9 of the 10 differences in the third comparison are also differences in the first comparison, suggesting that about half the differences between the 0-2 month sample and the 0-5 sample may be due to differences between the 0-2 month **sample** and the 0-2 **subsample** of the 0-5 sample.

The second comparison, comparing the 0-2 subsample to the 3-5 subsample, provides evidence both undermining and supporting the conclusion that a 0-5 sample will provide the same answers as two samples 0-2 and 3-5. On the one hand 5 of the fourteen differences in the third comparison are also in the first, the comparison of the 0-5 sample to the 0-2 sample, suggesting that the inclusion of 3-5 month olds in the 0-5 sample might contribute to the differences. On the other hand, in 5 out of the 8 indicators that are not different between the 0-2 and 0-5 samples, the 3-5 subsample more closely matches the 0-2 month sample than does the 0-2 subsample of the 0-5 sample. That this is true in general and not only for these extreme differences is suggested by the mean absolute differences between samples which are 5.2 for the differences between the 0-2 month **sample** and 3-5 month **subsample** and 6.6 for the differences between the 0-2 month **subsample** and the 3-5 month **subsample**.

Finally, the overall result of all these comparisons is the same as that of the comparison presented in the main text of the paper: in each comparison 85% or more of the indicators show no differences between the samples. The most consistent evidence of differencCae is between the 0-2 **subsample** of the 0-5 sample and the 0-2 month **sample**.
