# Supplementary material for: How well do mothers recall their own and their infants’ perinatal events? A two-district study using cross-sectional stratified random sampling in Bihar, India
Source: BMJ Open. 2019 Dec 18;9(12):e031289. doi: 10.1136/bmjopen-2019-031289 (PMC6937048; doi:10.1136/bmjopen-2019-031289)
Supplement: Supplementary data [file bmjopen-2019-031289supp005.pdf]

Table S4a: Point estimates and confidence intervals of indicators for subsamples of 0-2 and 3-5 month old infants in two districts: Bihar, India, 2015

Indicator[a]	District[b]	0-2 month subsample			3-5 month subsample			Difference		
		Point estimate	Confidence interval[c]		Point estimate	Confidence interval[c]		Point estimate	Confidence interval	
			Lower	Upper		Lower	Upper		Lower	Upper
1	1	79.2	71.9	86.5	75.2	66.0	84.4	4.0	-7.7	15.7
1	2	80.8	74.4	87.2	92.3	87.5	97.2	-11.5	-19.5	-3.5
2	1	36.1	27.8	44.4	41.5	31.7	51.3	-5.4	-18.2	7.4
2	2	46.5	38.1	54.8	50.6	40.3	61.0	-4.2	-17.5	9.1
3	1	81.0	75.0	87.1	79.5	71.5	87.5	1.5	-8.5	11.6
3	2	79.9	73.4	86.5	74.7	65.8	83.7	5.2	-5.9	16.3
4	1	61.8	53.1	70.4	65.2	55.0	75.3	-3.4	-16.7	10.0
4	2	62.7	54.7	70.7	58.4	48.4	68.3	4.4	-8.4	17.1
5	1	45.0	36.2	53.9	46.8	35.5	58.0	-1.7	-16.0	12.6
5	2	49.1	40.8	57.4	43.5	33.3	53.7	5.6	-7.6	18.7
6	1	60.3	51.9	68.7	59.8	49.6	70.0	0.5	-12.7	13.7
6	2	68.5	60.8	76.1	55.8	45.7	65.8	12.7	0.0	25.4
7	1	40.3	31.8	48.8	38.4	27.4	49.3	1.9	-11.9	15.8
7	2	74.5	67.2	81.9	69.8	60.3	79.3	4.7	-7.3	16.8
8	1	49.3	41.2	57.3	38.2	27.9	48.5	11.1	-2.0	24.2
8	2	60.0	52.0	67.9	46.4	36.7	56.2	13.6	1.0	26.1
9	1	39.8	31.3	48.4	44.4	33.9	54.8	-4.5	-18.0	9.0
9	2	44.7	36.6	52.8	41.7	31.5	51.9	3.0	-10.0	16.0
10	1	61.9	54.4	69.4	56.1	45.3	66.9	5.7	-7.4	18.9
10	2	63.9	56.1	71.7	50.5	40.3	60.6	13.5	0.7	26.2
11	1	98.4	96.3	100.6	97.6	94.3	100.9	0.8	-3.1	4.8
11	2	96.7	94.1	99.3	97.7	95.1	100.3	-1.0	-4.7	2.7
12	1	65.0	56.4	73.6	71.3	61.8	80.7	-6.3	-19.1	6.5
12	2	77.8	71.1	84.6	84.1	76.7	91.4	-6.2	-16.2	3.8
13	1	3.3	0.1	6.5	1.6	-1.5	4.8	1.7	-2.8	6.2
13	2	6.7	2.6	10.9	2.8	0.0	5.6	3.9	-1.1	8.9
14	1	2.2	-0.4	4.8	1.6	-1.5	4.8	0.6	-3.5	4.7
14	2	4.5	1.0	8.0	2.3	-0.4	4.9	2.2	-2.2	6.6
15	1	35.2	26.8	43.6	30.4	20.9	39.9	4.8	-7.8	17.5
15	2	56.8	48.7	64.9	54.8	44.3	65.3	2.0	-11.3	15.2
16	1	5.3	1.4	9.3	0.9	-0.8	2.6	4.5	0.2	8.7
16	2	16.6	10.2	23.0	11.0	4.8	17.1	5.7	-3.2	14.5
17	1	59.6	51.4	67.9	49.5	38.7	60.3	10.1	-3.5	23.7
17	2	64.4	56.5	72.4	59.9	49.8	70.0	4.5	-8.3	17.4
18	1	24.2	16.8	31.6	22.2	13.9	30.5	2.0	-9.1	13.1
18	2	45.9	38.1	53.8	40.3	32.0	48.6	5.7	-5.7	17.1
19	1	17.9	11.2	24.5	9.2	2.4	16.1	8.6	-0.9	18.2
19	2	40.5	32.4	48.6	37.7	29.0	46.4	2.8	-9.0	14.6
20	1	42.5	34.8	50.2	35.1	26.0	44.2	7.4	-4.5	19.4
20	2	59.4	51.7	67.0	50.7	42.3	59.1	8.6	-2.7	20.0
21	1	37.0	29.2	44.8	22.1	13.1	31.2	14.9	2.9	26.8
21	2	54.2	46.1	62.3	47.7	38.9	56.6	6.5	-5.6	18.5
22	1	62.5	54.0	70.9	54.7	44.8	64.6	7.8	-5.2	20.8
22	2	69.7	62.3	77.1	65.5	55.4	75.5	4.2	-8.2	16.7
23	1	47.5	39.1	55.9	46.1	35.0	57.3	1.4	-12.5	15.3

23	2	64.9	56.9	73.0	58.9	48.4	69.5	6.0	-7.3	19.3
24	1	75.1	67.4	82.9	75.3	66.0	84.7	-0.2	-12.4	11.9
24	2	67.6	59.7	75.5	81.0	72.7	89.3	-13.4	-24.8	-1.9
25	1	12.1	6.1	18.0	13.1	5.9	20.2	-1.0	-10.3	8.3
25	2	16.7	10.3	23.1	22.7	13.8	31.6	-6.0	-16.9	4.9
26	1	76.2	68.6	83.8	77.5	68.5	86.6	-1.3	-13.1	10.5
26	2	69.4	61.6	77.2	81.6	73.3	89.8	-12.2	-23.6	-0.8
27	1	50.4	41.5	59.3	43.4	33.0	53.9	6.9	-6.8	20.7
27	2	57.2	48.9	65.4	66.9	57.7	76.0	-9.7	-22.0	2.7
28	1	8.6	3.7	13.6	6.0	1.3	10.6	2.7	-4.1	9.5
28	2	9.8	5.1	14.5	19.7	11.4	28.1	-9.9	-19.5	-0.3
29	1	50.4	41.5	59.3	45.6	35.4	55.9	4.8	-8.8	18.4
29	2	59.2	51.1	67.4	69.7	60.5	79.0	-10.5	-22.9	1.8
30	1	32.7	24.5	41.0	36.3	25.9	46.7	-3.6	-16.9	9.7
30	2	49.2	41.0	57.4	54.7	44.3	65.1	-5.5	-18.8	7.7
31	1	42.0	33.0	51.1	49.2	39.0	59.5	-7.2	-20.9	6.5
31	2	48.6	40.3	56.8	58.9	48.8	69.0	-10.3	-23.3	2.7
32	1	3.9	0.4	7.3	3.2	-0.4	6.8	0.7	-4.3	5.7
32	2	9.4	4.2	14.5	10.7	4.7	16.8	-1.4	-9.3	6.5
33	1	43.6	34.5	52.6	51.4	41.1	61.7	-7.8	-21.6	5.9
33	2	52.9	44.8	61.0	61.1	51.1	71.2	-8.2	-21.1	4.7
34	1	7.0	2.5	11.5	14.2	7.0	21.4	-7.2	-15.7	1.2
34	2	8.6	3.8	13.4	9.5	4.0	15.0	-0.9	-8.2	6.4
35	1	57.2	48.2	66.2	62.5	52.7	72.4	-5.3	-18.7	8.0
35	2	63.2	55.1	71.4	77.6	68.6	86.6	-14.3	-26.5	-2.2
37	1	76.6	69.1	84.1	72.3	62.7	81.8	4.3	-7.8	16.4
37	2	81.6	74.9	88.3	91.3	85.8	96.8	-9.7	-18.4	-1.0
38	1	59.0	49.9	68.0	49.7	39.3	60.2	9.2	-4.6	23.1
38	2	56.1	47.8	64.4	69.7	61.2	78.2	-13.6	-25.5	-1.7
39	1	0.0	0.0	0.0	0.0	0.0	0.0	0.0	0.0	0.0
39	2	5.8	-3.1	14.7	9.6	-40.1	59.3	-3.8	-54.3	46.7
39.5	1	0.0	0.0	0.0	0.0	0.0	0.0	0.0	0.0	0.0
39.5	2	1.1	-0.4	2.6	0.7	-0.7	2.1	0.3	-1.7	2.4
40	1	17.6	10.9	24.4	13.6	7.5	19.8	4.0	-5.2	13.2
40	2	8.0	3.9	12.1	11.6	5.6	17.7	-3.7	-11.0	3.6
42	1	52.6	44.3	61.0	40.1	29.6	50.6	12.5	-0.9	25.9
42	2	47.5	39.4	55.6	44.6	34.4	54.8	2.9	-10.2	16.0
43	1	35.6	27.4	43.8	22.6	13.6	31.5	13.1	0.9	25.2
43	2	35.5	28.0	43.0	24.8	16.4	33.1	10.8	-0.5	22.0
44	1	10.1	4.2	16.0	17.6	9.5	25.7	-7.5	-17.6	2.5
44	2	20.6	14.1	27.0	16.2	8.7	23.8	4.3	-5.6	14.3
45	1	9.0	-4.7	22.7	13.7	-0.7	28.1	-4.7	-24.6	15.2
45	2	6.6	-9.9	23.2	14.4	14.4	14.4	-7.7	-24.3	8.8
46	1	63.4	54.6	72.2	52.8	41.5	64.0	10.7	-3.6	24.9
46	2	68.9	61.1	76.7	62.5	52.6	72.4	6.4	-6.2	19.0
47	1	33.0	24.7	41.4	32.5	22.2	42.9	0.5	-12.8	13.8
47	2	70.4	62.8	77.9	65.3	55.9	74.8	5.1	-7.1	17.2
48	1	68.8	60.6	77.0	63.2	52.5	73.8	5.7	-7.8	19.1
48	2	76.2	69.3	83.0	81.3	73.9	88.6	-5.1	-15.2	5.0
49	1	66.5	57.9	75.1	52.7	41.0	64.3	13.8	-0.6	28.3
49	2	68.6	59.9	77.3	64.4	53.8	75.0	4.2	-9.5	17.9
50	1	51.7	42.1	61.4	59.5	47.6	71.5	-7.8	-23.1	7.6

50	2	55.2	46.3	64.1	55.8	45.1	66.6	-0.7	-14.6	13.3
51	1	33.2	14.6	51.7	61.0	36.7	85.4	-27.9	-58.5	2.7
51	2	40.6	17.5	63.7	43.0	-6.7	92.7	-2.4	-57.2	52.4
52	1	73.0	65.1	80.9	39.7	28.6	50.7	33.3	19.8	46.9
52	2	83.8	78.2	89.3	45.8	35.6	55.9	38.0	26.4	49.5

a. For text see Table S1
b. 1 Aurangabad, 2 Gopalganj
c. Estimated with Stata command svy
