# Supplementary material for: How well do mothers recall their own and their infants’ perinatal events? A two-district study using cross-sectional stratified random sampling in Bihar, India
Source: BMJ Open. 2019 Dec 18;9(12):e031289. doi: 10.1136/bmjopen-2019-031289 (PMC6937048; doi:10.1136/bmjopen-2019-031289)
Supplement: Supplementary data [file bmjopen-2019-031289supp006.pdf]

Table S4b: Indicators in Table S4a where zero is not in the estimated confidence interval of difference between 0-2 and 3-5 month subsamples							
	Indicator	District[a]	Point estimate		Difference	Confidence interval[b]	
			0-2 month	3-5 month		Lower	Upper
			In Table S4a and in Table S2				
21	Proportion of mothers who planned institutional delivery of infants (0-2/0-5) months who arranged clean cloth for mothers and baby	1	37.0	22.1	14.9	2.9	26.8
24	Proportion of mothers of infants (0-2/0-5) months who were visited by ASHA at least once during their last pregnancy	2	67.6	81.0	-13.4	-24.8	-1.9
37	Proportion of mothers of infants (0-2/0-5) months whose last child was delivered at a health facility (private or public facility)	2	81.6	91.3	-9.7	-18.4	-1.0
38	Proportion of mothers of infants (0-2/0-5) months whose last child was delivered at public facility	2	56.1	69.7	-13.6	-25.5	-1.7
52	Proportion of infants (0-2/0-5) months who were breast-fed in the past	1	73.0	39.7	33.3	19.8	46.9
52	24 hours (Exclusively Breast-fed)	2	83.8	45.8	38.0	26.4	49.5
			In Table S4a but not in Table S2				
1	Proportion of mothers of infants (0-2/0-5) months who were registered during their last pregnancy	2	80.8	92.3	-11.5	-19.5	-3.5
8	Proportion of mothers of infants (0-2/0-5) months who attended at least one ANC where at least one abdominal examination was performed during her last pregnancy	2	60.0	46.4	13.6	1.0	26.1
10	Proportion of mothers of infants (0-2/0-5) months who attended at least one ANC where at least one blood test was performed during her last pregnancy	2	63.9	50.5	13.5	0.7	26.2
16	Proportion of mothers (home + institutional delivery) of infants (0-2/0-5) months who have identified anybody who would donate blood in the case of emergency in their last pregnancy	1	5.3	0.9	4.5	0.2	8.7
26	Proportion of mothers of infants (0-2/0-5) months who were visited by FLWs at least once during their last pregnancy	2	69.4	81.6	-12.2	-23.6	-0.8
28	Proportion of mothers of infants (0-2/0-5) months who were visited by AWW in the last trimester during their last pregnancy	2	9.8	19.7	-9.9	-19.5	-0.3
35	Proportion of mothers of infants (0-2/0-5) months who were visited home by any FLW within first week of last delivery	2	63.2	77.6	-14.3	-26.5	-2.2

43	Proportion of mothers (home + institutional delivery) of infants (0-2/0-5) months who have delivered baby practiced skin to skin care (STSC) immediately after birth	1	35.6	22.6	13.1	0.9	25.2

a. 1 = Aurangabad; 2 = Gopalganj
b. Calculated from standard errors for point estimates estimated with Stata command svy
