# Supplementary material for: How well do mothers recall their own and their infants’ perinatal events? A two-district study using cross-sectional stratified random sampling in Bihar, India
Source: BMJ Open. 2019 Dec 18;9(12):e031289. doi: 10.1136/bmjopen-2019-031289 (PMC6937048; doi:10.1136/bmjopen-2019-031289)
Supplement: Supplementary data [file bmjopen-2019-031289supp007.pdf]

Table S5a: Point estimates and confidence intervals of indicators for a sample 0-2 month old infants and a subsample of 0-2 month old infants from a sample of 0-5 month old infants in two districts: Bihar, India, 2015										
Indicator[a]	District[b]	0-2 month subsample			0-2 month sample			Difference		
		Point estimate	Confidence interval[c]		Point estimate	Confidence interval[c]		Point estimate	Confidence interval	
			Lower	Upper		Lower	Upper		Lower	Upper
1	1	79.2	71.9	86.5	85.2	80.2	90.2	-6.0	-14.8	2.8
1	2	80.8	74.4	87.2	85.0	80.5	89.6	-4.2	-12.1	3.6
2	1	36.1	27.8	44.4	43.0	36.3	49.8	-6.9	-17.6	3.8
2	2	46.5	38.1	54.8	47.5	41.5	53.6	-1.1	-11.3	9.2
3	1	81.0	75.0	87.1	83.8	78.8	88.8	-2.8	-10.7	5.1
3	2	79.9	73.4	86.5	80.6	75.7	85.5	-0.7	-8.8	7.5
4	1	61.8	53.1	70.4	61.1	54.4	67.8	0.7	-10.3	11.6
4	2	62.7	54.7	70.7	57.4	51.2	63.6	5.3	-4.8	15.4
5	1	45.0	36.2	53.9	43.2	36.4	50.1	1.8	-9.4	13.0
5	2	49.1	40.8	57.4	43.4	37.1	49.8	5.7	-4.8	16.2
6	1	60.3	51.9	68.7	58.9	52.3	65.6	1.4	-9.3	12.1
6	2	68.5	60.8	76.1	66.3	60.2	72.4	2.2	-7.6	12.0
7	1	40.3	31.8	48.8	42.0	35.3	48.8	-1.7	-12.6	9.2
7	2	74.5	67.2	81.9	69.2	63.2	75.2	5.3	-4.2	14.8
8	1	49.3	41.2	57.3	42.7	36.1	49.3	6.5	-3.9	16.9
8	2	60.0	52.0	67.9	52.4	46.2	58.7	7.6	-2.6	17.7
9	1	39.8	31.3	48.4	45.8	39.0	52.6	-5.9	-16.8	5.0
9	2	44.7	36.6	52.8	42.8	36.6	49.1	1.8	-8.3	12.0
10	1	61.9	54.4	69.4	67.2	60.9	73.6	-5.3	-15.2	4.5
10	2	63.9	56.1	71.7	54.8	48.5	61.1	9.1	-0.9	19.1
11	1	98.4	96.3	100.6	98.5	96.7	100.2	-0.0	-2.8	2.8
11	2	96.7	94.1	99.3	96.9	94.8	99.1	-0.2	-3.7	3.2
12	1	65.0	56.4	73.6	69.3	63.0	75.7	-4.3	-15.0	6.4
12	2	77.8	71.1	84.6	81.9	77.3	86.6	-4.1	-12.3	4.1
13	1	3.3	0.1	6.5	4.0	1.2	6.7	-0.6	-4.9	3.6
13	2	6.7	2.6	10.9	3.0	1.0	5.0	3.7	-0.9	8.3
14	1	2.2	-0.4	4.8	1.6	-0.2	3.4	0.7	-2.5	3.8
14	2	4.5	1.0	8.0	1.7	0.2	3.2	2.8	-1.1	6.7
15	1	35.2	26.8	43.6	25.9	20.1	31.6	9.3	-0.8	19.5
15	2	56.8	48.7	64.9	45.7	39.6	51.8	11.1	0.9	21.2
16	1	5.3	1.4	9.3	4.3	1.5	7.0	1.1	-3.7	5.9
16	2	16.6	10.2	23.0	9.7	6.1	13.3	7.0	-0.4	14.3
17	1	59.6	51.4	67.9	48.5	41.8	55.2	11.1	0.5	21.7
17	2	64.4	56.5	72.4	51.8	45.6	58.0	12.7	2.6	22.7
18	1	24.2	16.8	31.6	26.1	20.2	32.1	-1.9	-11.4	7.6
18	2	45.9	38.1	53.8	38.2	33.0	43.3	7.8	-1.6	17.1
19	1	17.9	11.2	24.5	23.5	16.4	30.7	-5.7	-15.5	4.1
19	2	40.5	32.4	48.6	43.4	37.3	49.5	-2.9	-13.0	7.3
20	1	42.5	34.8	50.2	44.1	38.3	50.0	-1.6	-11.3	8.0
20	2	59.4	51.7	67.0	50.0	44.7	55.4	9.3	0.0	18.7
21	1	37.0	29.2	44.8	43.6	36.7	50.5	-6.6	-17.0	3.8
21	2	54.2	46.1	62.3	55.6	49.5	61.7	-1.4	-11.6	8.7
22	1	62.5	54.0	70.9	65.5	59.0	72.0	-3.0	-13.7	7.7
22	2	69.7	62.3	77.1	60.2	54.0	66.3	9.5	-0.1	19.2

23	1	47.5	39.1	55.9	62.5	54.9	70.2	-15.0	-26.4	-3.6
23	2	64.9	56.9	73.0	68.0	61.2	74.8	-3.1	-13.7	7.4
24	1	75.1	67.4	82.9	62.2	55.5	68.9	12.9	2.7	23.2
24	2	67.6	59.7	75.5	63.5	57.4	69.7	4.1	-5.9	14.1
25	1	12.1	6.1	18.0	11.1	7.0	15.1	1.0	-6.2	8.2
25	2	16.7	10.3	23.1	19.3	14.4	24.2	-2.6	-10.6	5.5
26	1	76.2	68.6	83.8	63.5	56.8	70.1	12.7	2.6	22.8
26	2	69.4	61.6	77.2	66.9	60.9	73.0	2.4	-7.4	12.3
27	1	50.4	41.5	59.3	42.3	35.6	49.1	8.1	-3.1	19.3
27	2	57.2	48.9	65.4	52.6	46.3	58.9	4.6	-5.8	15.0
28	1	8.6	3.7	13.6	8.2	4.6	11.8	0.5	-5.7	6.6
28	2	9.8	5.1	14.5	15.4	11.0	19.8	-5.6	-12.0	0.8
29	1	50.4	41.5	59.3	43.3	36.5	50.1	7.1	-4.1	18.3
29	2	59.2	51.1	67.4	55.9	49.7	62.2	3.3	-7.0	13.6
30	1	32.7	24.5	41.0	28.7	22.6	34.7	4.1	-6.1	14.3
30	2	49.2	41.0	57.4	46.9	40.5	53.3	2.3	-8.1	12.7
31	1	42.0	33.0	51.1	29.9	23.5	36.3	12.1	1.1	23.2
31	2	48.6	40.3	56.8	44.5	38.2	50.8	4.1	-6.3	14.5
32	1	3.9	0.4	7.3	5.3	2.2	8.5	-1.5	-6.2	3.2
32	2	9.4	4.2	14.5	9.1	5.7	12.6	0.2	-6.0	6.4
33	1	43.6	34.5	52.6	32.2	25.8	38.7	11.3	0.2	22.5
33	2	52.9	44.8	61.0	48.2	41.9	54.5	4.7	-5.5	15.0
34	1	7.0	2.5	11.5	6.2	2.8	9.6	0.8	-4.8	6.4
34	2	8.6	3.8	13.4	14.4	9.8	19.0	-5.8	-12.5	0.8
35	1	57.2	48.2	66.2	44.5	37.7	51.4	12.7	1.3	24.0
35	2	63.2	55.1	71.4	62.5	56.4	68.7	0.7	-9.5	10.9
37	1	76.6	69.1	84.1	72.9	66.7	79.1	3.7	-6.0	13.4
37	2	81.6	74.9	88.3	78.9	73.5	84.2	2.7	-5.8	11.3
38	1	59.0	49.9	68.0	54.7	47.8	61.7	4.2	-7.2	15.6
38	2	56.1	47.8	64.4	51.2	44.7	57.6	4.9	-5.6	15.4
39	1	0.0	0.0	0.0	5.4	-0.9	11.8	-5.4	-11.8	0.9
39	2	5.8	-3.1	14.7	9.1	1.3	16.9	-3.3	-15.1	8.5
40	1	0.0	0.0	0.0	1.4	-0.2	3.0	-1.4	-3.0	0.2
40	2	1.1	-0.4	2.6	1.8	0.2	3.4	-0.7	-2.9	1.4
40	1	17.6	10.9	24.4	10.6	6.5	14.7	7.0	-0.9	14.9
40	2	8.0	3.9	12.1	8.5	4.9	12.2	-0.6	-6.0	4.9
42	1	52.6	44.3	61.0	43.8	36.9	50.6	8.9	-2.0	19.7
42	2	47.5	39.4	55.6	41.8	35.6	48.1	5.7	-4.6	15.9
43	1	35.6	27.4	43.8	25.6	19.8	31.5	10.0	-0.1	20.1
43	2	35.5	28.0	43.0	26.9	21.5	32.4	8.6	-0.7	17.8
44	1	10.1	4.2	16.0	12.2	7.3	17.1	-2.1	-9.8	5.6
44	2	20.6	14.1	27.0	21.6	16.3	26.8	-1.0	-9.3	7.3
45	1	9.0	-4.7	22.7	7.8	0.4	15.2	1.2	-14.3	16.8
45	2	6.6	-9.9	23.2	5.2	0.3	10.1	1.4	-15.8	18.7
46	1	63.4	54.6	72.2	56.6	49.9	63.2	6.9	-4.2	17.9
46	2	68.9	61.1	76.7	61.7	55.5	67.9	7.2	-2.8	17.2
47	1	33.0	24.7	41.4	35.9	29.3	42.5	-2.8	-13.5	7.8
47	2	70.4	62.8	77.9	66.6	60.6	72.7	3.7	-6.0	13.4
48	1	68.8	60.6	77.0	64.9	58.5	71.4	3.9	-6.5	14.3
48	2	76.2	69.3	83.0	70.7	64.9	76.5	5.4	-3.6	14.4
49	1	66.5	57.9	75.1	64.5	57.1	71.8	2.1	-9.2	13.3
49	2	68.6	59.9	77.3	67.3	60.6	73.9	1.3	-9.7	12.3

50	1	51.7	42.1	61.4	48.4	40.3	56.4	3.4	-9.2	15.9
50	2	55.2	46.3	64.1	56.1	49.0	63.2	-0.9	-12.3	10.4
51	1	33.2	14.6	51.7	78.0	65.9	90.2	-44.9	-67.0	-22.7
51	2	40.6	17.5	63.7	63.7	48.8	78.5	-23.0	-50.5	4.5
52	1	73.0	65.1	80.9	69.2	62.7	75.6	3.8	-6.3	14.0
52	2	83.8	78.2	89.3	82.1	77.1	87.0	1.7	-5.7	9.1

a. For text see Table S1
b. 1 Aurangabad, 2 Gopalganj
c. Estimated with Stata command svy
