# Supplementary material for: How well do mothers recall their own and their infants’ perinatal events? A two-district study using cross-sectional stratified random sampling in Bihar, India
Source: BMJ Open. 2019 Dec 18;9(12):e031289. doi: 10.1136/bmjopen-2019-031289 (PMC6937048; doi:10.1136/bmjopen-2019-031289)
Supplement: Supplementary data [file bmjopen-2019-031289supp008.pdf]

Table S5b: Point estimates of weighted indicators and differences between a sample of mothers of 0-2 month old infants and a subsample of mothers of 0-2 month old infants from a sample of mothers of 0-5 month old infants in two districts of Bihar, India

	Indicator	District[a]	Point estimates		Difference		
					Estimate	Confidence interval[b]	
			Subsample	Sample		Lower	Upper
15	Proportion of mothers (home + institutional delivery) of infants (0-2/0-5) months who planned transportation to health facility in their last pregnancy	2	56.8	45.7	11.1	0.9	21.2
17	Proportion of mothers (home + institutional delivery) of infants (0-2/0-5) months who have identified persons who would take care of the baby immediately after birth	1	59.6	48.5	11.1	0.5	21.7
		2	64.4	51.8	12.7	2.6	22.7
23	Proportion of mothers who planned for institutional delivery of infants (0-2/0-5) months identified person to accompany her during the delivery	1	47.5	62.5	-15.0	-26.4	-3.6
24	Proportion of mothers of infants (0-2/0-5) months who were visited by ASHA at least once during their last pregnancy	1	75.1	62.2	12.9	2.7	23.2
26	Proportion of mothers of infants (0-2/0-5) months who were visited by FLWs at least once during their last pregnancy	1	76.2	63.5	12.7	2.6	22.8
31	Proportion of mothers of infants (0-2/0-5) months who were visited home by ASHA within 24 hours of last delivery	1	42.0	29.9	12.1	1.1	23.2
33	Proportion of mothers of infants (0-2/0-5) months who were visited home by any FLW within 24 hours of last delivery	1	43.6	32.2	11.3	0.2	22.5
35	Proportion of mothers of infants (0-2/0-5) months who were visited home by any FLW within first week of last delivery	1	57.2	44.5	12.7	1.3	24.0
51	Proportion of infants aged (0-2/0-5) months who were delivered at home continued with dry cord care	1	33.2	78.0	-44.9	-67.0	-22.7

a. 1 = Aurangabad; 2 = Gopalganj
b. Calculated from standard errors for point estimates estimated with Stata command svy
