# Supplementary material for: How well do mothers recall their own and their infants’ perinatal events? A two-district study using cross-sectional stratified random sampling in Bihar, India
Source: BMJ Open. 2019 Dec 18;9(12):e031289. doi: 10.1136/bmjopen-2019-031289 (PMC6937048; doi:10.1136/bmjopen-2019-031289)
Supplement: Supplementary data [file bmjopen-2019-031289supp009.pdf]

Table S6: Point estimates and confidence intervals of indicators for a sample of 3-5 month infants and subsample of 3-5 month infants in two districts: Bihar, India, 2015

Indicator[a]	District[b]	3-5 month subsample			3-5 month sample			Difference		
		Point estimate	Confidence interval[c]		Point estimate	Confidence interval[c]		Point estimate	Confidence interval	
			Lower	Upper		Lower	Upper		Lower	Upper
37	1	72.3	62.7	81.8	70.4	64.2	76.5	1.9	13.3	-9.5
37	2	91.3	85.8	96.8	86.2	81.8	90.6	5.1	12.2	-2.0
38	1	49.7	39.3	60.2	45.8	38.9	52.6	4.0	16.4	-8.5
38	2	69.7	61.2	78.2	64.4	58.3	70.5	5.4	15.8	-5.1
52	1	39.7	28.6	50.7	65.3	58.8	71.8	-25.6	-12.8	-38.4
52	2	45.8	35.6	55.9	55.2	49.1	61.2	-9.4	2.4	-21.2

a. For text see Table S1
b. 1 Aurangabad, 2 Gopalganj
c. Estimated with Stata command svy
