# Supplementary material for: How well do mothers recall their own and their infants’ perinatal events? A two-district study using cross-sectional stratified random sampling in Bihar, India
Source: BMJ Open. 2019 Dec 18;9(12):e031289. doi: 10.1136/bmjopen-2019-031289 (PMC6937048; doi:10.1136/bmjopen-2019-031289)
Supplement: Author's manuscript [file bmjopen-2019-031289.draft_revisions.pdf]

BMJ Open is committed to open peer review. As part of this commitment we make the peer review history of every article we publish publicly available.

When an article is published we post the peer reviewers' comments and the authors' responses online. We also post the versions of the paper that were used during peer review. These are the versions that the peer review comments apply to.

The versions of the paper that follow are the versions that were submitted during the peer review process. They are not the versions of record or the final published versions. They should not be cited or distributed as the published version of this manuscript.

BMJ Open is an open access journal and the full, final, typeset and author-corrected version of record of the manuscript is available on our site with no access controls, subscription charges or pay-per-view fees (http://bmjopen.bmj.com).

If you have any questions on BMJ Open's open peer review process please email info.bmjopen@bmj.com

BMJ Open

How well do mothers recall their own and their infant’s perinatal events? a two district study using cross-sectional stratified random sampling in Bihar, India

Journal:	*BMJ Open*
Manuscript ID	bmjopen-2019-031289
Article Type:	Research
Date Submitted by the Author:	26-Apr-2019
Complete List of Authors:	Valadez, Joseph; Liverpool School of Tropical Medicine, International Public Health<br>Devkota, Baburam; Liverpool School of Tropical Medicine, International Public Health<br>Jeffery, Caroline; Liverpool School of Tropical Medicine<br>Hadden, Wilbur; University of Maryland at College Park, Sociology
Keywords:	Public health < INFECTIOUS DISEASES, maternal recall, Bihar, India, perinatal, surveys, LQAS

SCHOLARONE™
Manuscripts

How well do mothers recall their own and their infant's perinatal events? a two district study using cross-sectional stratified random sampling in Bihar, India

Joseph J Valadez[1*], Baburam Devkota[1], Caroline Jeffery[1], Wilbur C. Hadden[2]

[1] Department of International Public Health, Liverpool School of Tropical Medicine, Liverpool, United Kingdom

[2] Department of Sociology, Center for Innovation, University of Maryland, USA

* Corresponding author: Professor Joseph J Valadez, Department of International Public Health, Liverpool School of Tropical Medicine, Pembroke Place, Liverpool, L3 5QA United Kingdom. Email: joseph.valadez@lstmed.ac.uk; Mobile: +44(0)754 8523717

Manuscript word length: 2255

For peer review only

Abstract

INTRODUCTION

Global monitoring of maternal, newborn and child health (MNCH) programmes use self-reported data subject to recall error which leads to incorrect decisions for improving health services and wasted resources. To minimize this risk, samples of mothers of infants aged 0-2 and 3-5 months are sometimes used. However, sampling stratification may be unnecessary, if a single sample of mothers of infants 0-5 months provides the same information.

METHODS

An annual MNCH household survey in two districts of Bihar, India (N=6million), collected data from independent samples of mothers of infants 0-5, 0-2 and 3-5 months. Analyses compare responses from the first two samples (n=950) to assess recall bias using 51 indicators with Mantel-Haenszel-Cochran statistics.

RESULTS

No meaningful differences are detected in 79.4% comparisons; while 12.7% display differences. The difference detected for exclusive breastfeeding is due to early commencement of complementary-feeding by older infants. Other differences may be due to seasonal effects: mothers of older infants 3-5 months were pregnant during

For peer review only

the dry season; mothers of children 0-2 months were pregnant during the monsoons, making transportation difficult.

CONCLUSIONS

The same district coverage estimates can be obtained by sampling mothers with infants 0-5 months as with two cohorts: 0-2 and 3-5 months suggesting that mothers of young infants recall their own perinatal events and those of their children. Needless stratification wastes resources, does not improve the quality of information, and increases the burden placed on data collectors and communities. This burden can increase non-sampling error.

STRENGTHS AND LIMITATIONS OF THIS STUDY

- Strength: The data were produced using stratified random sampling with no apparent design effect leading to an efficient use of information.
- Strength: Data were collected from female participants by female data collectors which is likely to have reduced non-sampling error.
- Strength: The study population is very large covering a large geographical area, reducing the likelihood that the results are pertinent only to a small group of mothers with infants; results may be generalizable.
- Strength: Both weighted and unweighted results are presented giving strength to the conclusions.
- Limitation: The study is confined to assessing data in the 0-2 month cohort vis a vis a 0-5 month cohort of infants; journal space limitations prevent expanding the

For peer review only - http://bmjopen.bmj.com/site/about/guidelines.xhtml

research to assess indicators from the 3-5 month cohort vis a vis the 0-5 month cohort.

Key Words:

Maternal recall, surveys, maternal health, MNCH, LQAS, Bihar, India

INTRODUCTION

The progress toward United Nations' Sustainable Development Goal (SDG) 5 is measured with 9 targets, including the Maternal Mortality Ratio (MMR), and neonatal and under 5-year mortality rates (CMR) [1 2]. In India, Bihar is one of the largest (N=110 million) and poorest states (53% of households are in the lowest wealth index quintile [3]) with high child and maternal mortality (CMR=54, MMR=208) [4] (see [5] for an evaluation of the health care system in Bihar), and is a priority for donor support for health systems strengthening.

To accelerate progress toward achieving SDG 5, state governments in India pursue programs of community-based care (see [6 7] for descriptions and assessments of this approach). Since 2011, the Bihar Ministry of Health has supported an Integrated Family Health Initiative to improve

For peer review only - http://bmjopen.bmj.com/site/about/guidelines.xhtml

the availability, quality and use of prenatal, perinatal and postnatal care for mothers and infants [8].

The usual way to monitor progress toward achieving an SDG is with household surveys. Perhaps the most commonly used surveys are cluster sample surveys such as the Demographic and Health Surveys and the Multiple Indicator Cluster Surveys [9 10]. An alternative design is Lot Quality Assurance Sampling (LQAS), which provides comparable data but is decentralized to the local organisation of health services and more useful for management and program planning [11]. Several states in India find it benefits their programs [12]. Surveys rely on the reports of mothers of infants and young children, but these reports are subject to several sources of potential error and bias through interviewees not knowing, forgetting, and having memory errors [13 14]. Nevertheless, studies have shown both that mothers can accurately report significant facts about the birth and care of their children many years after the event [15], but that even immediately after giving birth mothers may misreport details [16-18]. Studies of maternal recall of her child's vaccination status concluded that due to offsetting errors of maternal reports, the resulting data accurately measured vaccination rates [19]; the pattern of error revealed that mothers whose children are up-to-date or nearly so

For peer review only

For peer review only - http://bmjopen.bmj.com/site/about/guidelines.xhtml

tended to underestimate their child's vaccination status whilst mothers whose children have few vaccinations, overestimate their coverage.

To improve the validity of collected data, knowledge, practice and coverage surveys have used dedicated samples of mothers of infants 0-11 months of age or 0-5 months of age and children 6-11 months of age. In Bihar, local organisations departed from this convention of sampling two cohorts of children, by monitoring their program's progress by sampling five dedicated cohorts: mothers of children 0-2, 3-5, 6-8, 9-11 and 12-23 months old. Indicators focused on antenatal care, safe delivery practices, infant and young child feeding practices, immunisation, treatment seeking, and more. To avoid the possibility of maternal recall error, if such exists, each of the five cohorts was asked questions particularly relevant to a child's specific age group.

In countries, such as India, with high maternal and child mortality, regular monitoring of related health service coverage is critical to reducing these rates. However, the survey designs should be affordable and sustainable for the local health system; they should also produce precise, unbiased estimates [20]. In this study, we explore whether information is gained by sampling several

For peer review only - http://bmjopen.bmj.com/site/about/guidelines.xhtml

cohorts of children, with each having a short age range, or whether the sample can be reduced by 50% using few age cohorts each having a longer age range.

The research question we address is: “Do the health service delivery coverage estimates from a sample of mothers of infants aged 0-5 months differ from those obtained from a sample of mothers of infants ages 0-2 months?” A corollary to this question is: “Do mothers of infants 3-5 months of age display recall error relative to mothers of infants 0-2 months of age for antenatal, delivery or young infant health practices?” We compare district coverage estimates obtained from two independent samples of infants 0-2 months and 0-5 months of age. The implications of this study are important for health systems researchers needing results to appraise and improve their programmes.

METHODS

To answer this question, we collected information from a sample of mothers with infants 0-5 months old and a sample of mothers with 0-2 month old infants in two districts. This study took place within the context of a larger survey that also sampled children 3-5, 6-8, 9-11 and 12-23 months of age. However, we only use the two

For peer review only - http://bmjopen.bmj.com/site/about/guidelines.xhtml

aforementioned groups to assess recall and refer only to them for the remainder of this paper. In application, the household sampling survey design we used was a stratified random sampling. Within each district, the strata are administrative units of the health system, which in Bihar is called a *block*. Within each block the primary sampling unit is the Anganwadi Centre (Community Health Subcentre) Catchment Area (ACCA). From each ACCA one respondent is randomly selected from each age-group under study following the standard procedure [21]. There are 14 and 11 blocks in Gopalganj and Aurangabad (N=6 million), the two districts selected for this study, respectively. The sampling design used lot quality assurance sampling (LQAS) approaches. A commonly used random sample size of 19 individuals in each target groups was selected in each block using probability proportional to size [22 23].

Within each sampling location (typically a village) one person from each target group was randomly sampled using segmentation sampling [24 25]. The total sample sizes are: (a) Gopalganj: 19 x 14 blocks= 266 infants 0-2 months and 266 infants 0-5 months, and (b) Aurangabad: 19 x 11 blocks= 209 infants 0-2 months and 209 infants 0-5 months.

Using summary data from each of the two samples we analyse the data with Cochran-Mantel-Haenszel (CMH) [26] tests

For peer review only

for 51 dichotomous indicators (See Supplementary File Table S1) common to the two samples. With a sufficient number of respondents or a sufficient number of blocks, the CMH test is equivalent to conditional logistic regression [27: 114-115]. In this analysis both the number of respondents and the number of blocks only approach sufficiency. Consequently, the calculated statistics must be considered as approximations.

We calculate both unweighted and weighted estimates (See Supplementary File Table S2). The unweighted estimates permit the results from smaller blocks to have equal weight vis à vis larger ones. Since the research question concerns an analysis of which age cohort is most informative, the weighted estimates may not be as useful as the unweighted ones. However, the weighted estimates provide better point estimates at the district level. The effect of the weights on the Chi-square statistics is to increase the contribution of the larger blocks and decrease the contribution of the smaller blocks. Hence, we report both sets of results.

The CMH tests have a Chi-square distribution with 1 degree of freedom. This distribution puts the differences between the districts on a readily interpretable probability scale (See Supplementary File Table S2). To

For peer review only - http://bmjopen.bmj.com/site/about/guidelines.xhtml

determine meaningful differences in responses between the two age cohorts we used a probability of 0.05 as a cut-off value and consider differences with probabilities less than 0.05 to be possibly meaningful and those with larger probabilities to be likely due to sampling errors. With 102 comparisons (51 indicators weighted or unweighted) we must expect some to exceed this cut-off by chance alone. If all of the comparisons were independent, we might randomly find 5 differences, but many of the indicators measure related events (e.g., number of ANC visits and tetanus toxoid vaccinations) and the weighted and unweighted estimates are similar, so these indicators are not all independent and it is not possible to calculate an expected number of differences.

Patient and Public Involvement

This study does not involve patients. Also, the public were not involved in the design, conduct and reporting of the research. The public was engaged as interviewees. To ensure local engagement we coordinated with the Bihar Ministry of Health, local implementing non-governmental organizations and our donor. We also shared the results with them and offered further dissemination of results.

For peer review only - http://bmjopen.bmj.com/site/about/guidelines.xhtml

RESULTS

We find a high level of agreement between the two samples (Table 1). Out of 102 weighted and unweighted comparisons between the estimates from the 0-2 month and 0-5 month samples there is no probable difference in 81 (79.4%) in both the unweighted and weighted estimates. We detect that probable differences exist for 13 comparisons (12.7%). For the remaining eight comparisons the weighted and unweighted estimates disagree. The weighted estimates find seven differences that the unweighted estimates do not; the unweighted estimates find one difference that the weighted estimates do not find.

Table 1. Number of indicators by probability of a difference between the 0-2 months and the 0-5 months samples for weighted and unweighted samples

Unweighted	Weighted					
	Aurangabad		Gopalganj		Total	
	>= .05	< .05	>= .05	< .05	>= .05	< .05
>= .05	40	3	41	4	81	7
< .05	0	8	1	5	1	13

For different health service domains, the number of indicator comparisons varies from two (Exclusive Breastfeeding-EBF) to 24 concerning home visits by Front Line Workers' (FLW) support (Table 2). The two principal

For peer review only

FLW are Anganwadi workers and Accredited Social Health Activists (ASHA).

For peer review only

For peer review only - http://bmjopen.bmj.com/site/about/guidelines.xhtml

Table 2. Number of indicator comparisons by subject domain showing probable difference using weighted and unweighted estimates of 0-2 months and 0-5 months samples

Health Service Domain	Total comparisons	No Difference between 0-2 and 0-5 months results	Difference between 0-2 and 0-5 months results			
			Both	Unweighted only	Weighted only	Percent (%) indicators with different results
ANC & birth preparedness	22	21	0	0	1	5
Birth history & newborn care	8	6	1	0	1	25
Birth preparedness	18	13	3	0	2	28
Dry cord	2	2	0	0	0	0
FLW support	24	16	6	0	2	33
Maternal health	8	8	0	0	0	0
Neonatal health	18	15	1	1	1	17
Exclusive Breastfeeding	2	0	2	0	0	100
Totals	102	81	13	1	7	21.9

For peer review only - http://bmjopen.bmj.com/site/about/guidelines.xhtml

For peer review only

1

In the FLW support domain 33% of comparisons have probable differences. The birth preparedness and neonatal health domains each have 18 indicators; in these domains 28% and 17% show probable differences, respectively. Birth history and newborn care only have eight indicators and 25%, or two indicators, show difference. The differences between the two samples cluster around home visits from FLW and behaviours associated with birth preparedness, and newborn care. Details of these differences are listed in Table 3.

For two indicators, both the weighted and unweighted estimates display probable differences between the 0-2 and 0-5 months samples in both districts. For indicator #52, the proportion exclusively breastfeeding, the 0-5 months cohort has the lower estimate, and indicator #24, the proportion of mothers visited by an ASHA at least once during their last pregnancy, the 0-5 months sample gives the higher estimate, about 74%, compared to 63% in the 0-2 months sample.

For peer review only - http://bmjopen.bmj.com/site/about/guidelines.xhtml

Table 3. Indicators by health service domain exhibiting measurement differences between 0-2 months and 0-5 months samples

Health Service Domain and Indicator	Indicator No.	District	Weighted Coverage (%)		p-value		Estimate type
			0-2 months	0-5 months	Unweighted Estimate	Weighted Estimate	
**ANC & birth preparedness**							
Proportion of mothers of infants (0-2/0-5 months) registered during their last pregnancy	1	Aurangabad	85.2	77.6	0.0552	0.0365	Weighted
**Birth history & newborn care**							
Proportion of mothers of infants (0-2/0-5 months) whose last child was delivered at a public facility	38	Gopalganj	51.2	61.6	0.0363	0.0159	Both
Proportion of mothers of infants (0-2/0-5 months) whose last child was delivered at a health facility (private or public facility)	37	Gopalganj	78.9	85.5	0.0643	0.0459	Weighted
**Birth preparedness**							
Proportion of mothers of infants (0-2/0-5 months) who planned transportation to health facility in their last pregnancy (home & institutional delivery)	15	Gopalganj	45.7	56	0.0266	0.0158	Both
Proportion of mothers of infants (0-2/0-5 months) who identified persons to care for the baby immediately after birth (home + institutional delivery)	17	Gopalganj	51.8	62.6	0.0255	0.0103	Both
Proportion of mothers of infants (0-2/0-5 months) who planned for institutional delivery and	23	Aurangabad	62.5	47	0.0039	0.0052	Both

For peer review only - http://bmjopen.bmj.com/site/about/guidelines.xhtml

identified person to accompany her during the delivery							
Proportion of mothers who planned for institutional delivery of infants (0-2/0-5 months) who had a new blade & thread for their delivery	19	Aurangabad	23.5	14.5	0.062	0.0429	Weighted
Proportion of mothers who planned institutional delivery of infants (0-2/0-5 months) who arranged clean cloth for mothers and baby	21	Aurangabad	43.6	31.2	0.0546	0.0137	Weighted
**Front Line Workers support**							
Proportion of mothers of infants (0-2/0-5 months) who were visited by ASHA at least once during their last pregnancy	24	Aurangabad	62.2	75.2	0.0023	0.0042	Both
	24	Gopalganj	63.5	73	0.0284	0.0175	Both
Proportion of mothers of infants (0-2/0-5 months) visited at home by FLWs at least once during their last pregnancy	26	Aurangabad	63.5	76.7	0.0021	0.0032	Both
Proportion of mothers of infants (0-2/0-5 months) visited at home by ASHA within 24 hours of last delivery	31	Aurangabad	29.9	44.9	0.0009	0.0016	Both
Proportion of mothers of infants (0-2/0-5 months) visited at home by any FLW within 24 hours of last delivery	33	Aurangabad	32.2	46.7	0.0015	0.0026	Both
Proportion of mothers of infants (0-2/0-5 months) visited at home by any FLW within first week of last delivery	35	Aurangabad	44.5	59.3	0.0018	0.0026	Both
Proportion of mothers of infants (0-2/0-5 months) visited at home by any AWW within the first week of the last delivery	34	Gopalganj	14.4	8.9	0.0959	0.0471	Weighted
Proportion of mothers of infants (0-2/0-5 months) visited by	27	Gopalganj	52.6	61.1	0.0617	0.0449	Weighted

For peer review only

ASHAs at least once during their last pregnancy							
**Neonatal health**							
Proportion of infants aged (0-2/0-5 months) delivered at home continued with dry cord care	51	Aurangabad	78	45.4	0.0001	0.0006	Both
Proportion of infants aged (0-2/0-5 months) who were delivered at home continued with dry cord care	51	Gopalganj	63.7	41.1	0.0431	0.0627	Unweighted
Proportion of infants aged (0-2/0-5 months) weighed after birth (Public facility/Private facility/Home)	48	Gopalganj	70.7	78.2	0.0727	0.0464	Weighted
**Exclusive Breastfeeding**							
Proportion of infants (0-2/0-5 months) breast-fed in the past 24 hours (Exclusively Breast-fed)	52	Aurangabad	69.2	59.7	0.0229	0.0411	Both
	52	Gopalganj	82.1	68.4	0.0001	0.0003	Both

For peer review only

DISCUSSION

Statement of principal findings

There are no meaningful differences in coverage estimates for 79.4% (81 comparisons) of the indicator comparisons between the samples of mothers with infants 0-2 months old versus mothers of infants 0-5 months old; 12.7% (13 comparisons) display meaningful differences. The remaining 7.8% (eight comparisons) display discrepancies between the weighted and unweighted estimates.

Strengths and weaknesses of the study

The strengths of this study are that it compares estimates from two independent samples and that there are many estimates from diverse domains. The weaknesses of this study are that the data were collected in only 2 districts of 1 state in India and at a particular point in time.

Strengths and weaknesses in relation to other studies

Other studies of maternal recall bias have sought a "gold standard" to represent reality and to evaluate measures. Our study, of course, is interested in reality, but this study compares alternative measures needed to assess the Bihar health program. It also uses a complete

For peer review only

sample of the age grouping under study rather than just a sub-sample of a larger age grouping. Therefore, no statistical power is lost in this analysis.

Meaning of the study

The evidence indicates that the samples of the two age groups yield comparable results and that little more is learned by doubling the total sample by measuring independently 0-2 months and 3-5 months cohorts of children. These results also tend to dispel the hypothesis that maternal recall is problematic for mothers during the first 6 months following delivery. Our results are more consistent with conclusions presented in earlier research [15] as our results show no evidence of maternal recall error in this period in the 0-5 month cohort.

Indicator #52, EBF, displayed two comparisons measuring differences in both districts, which we can explain. The 0-5 month cohort had lower estimates. This finding is not surprising as fewer infants are expected to be exclusively breast fed in a sample ranging from 0 to 5 months than a sample ranging 0 to 2 months; mothers introduce complementary feeding and liquids as infants age despite this being a health risk. Hence, the EBF estimate is reduced as 3-5 month olds are introduced. Program

For peer review only - http://bmjopen.bmj.com/site/about/guidelines.xhtml

managers could understand better the early commencement of complementary feeding by assessing the proportion of children lost to EBF in each month cohort. Such an analysis can be done adequately with the 0-5 month dataset.

CONCLUSIONS

Overall, the answer to the research question, "Can one get the same district coverage estimates from a sample of mothers of infants aged 0-5 months as from a sample of mothers of infants ages 0-2 months?" is yes. This result can be paraphrased as: mothers do not display recall errors of their perinatal health care behaviour in a cohort of mothers with infants 0-5 months as compared with mothers with younger infants. Substantial resources and effort can be saved using a survey design, that avoids needless expenses to collect data that provides insubstantial amounts of information. It also reduces the burden places on data collectors and community participants. Fatigue to both groups can result in needless non-sampling error.

Unanswered questions and future research

Further investigation and consideration of the differences is warranted. The eight differences found in the FLW support indicators deserve more scrutiny. Seven show higher estimates for the 0-5 cohort and one has a

For peer review only - http://bmjopen.bmj.com/site/about/guidelines.xhtml

higher estimate for the 0-2 month cohort. The former seven differences may be due to excessive rainfall during the July-September (monthly average 318.95mm, range:195.99-395.8mm) versus the lesser rainfall during June-April (monthly average 25.52mm, range: 0.3-27.88mm) which in the last trimester may have reduced the access of ASHA in the 0-2 month cohort [28]. Indicators such as these may be particularly sensitive to rainfall and may explain why more mothers in the 0-5 month cohort displayed higher FLW visitation estimates since FLW were not impeded by the monsoon and the resulting muddy roads.

ACKNOWLEDGMENTS

We gratefully acknowledge the essential roles of Hemant Das, Alok Prahdan and Sanjay Biswa for their careful field work supporting the LQAS survey and the HMIS data retrieval. We thank Prof Imelda Bates, Prof Brian Faragher and Nancy Vollmer for their valuable feedback on an earlier version of this manuscript. This work was supported by the Bill and Melinda Gates Foundation (Investment ID OPP1142889).

ETHICS

The Ethical Committees of the authors' institutes approved the protocol, study instruments and consent procedures for the survey data collection.

For peer review only - http://bmjopen.bmj.com/site/about/guidelines.xhtml

COMPETING INTERESTS

The authors have read the statement on competing interests and declare that they have none.

AUTHORS' CONTRIBUTIONS

JJV, BD developed the research question and survey design; WH, CJ carried out the statistical analyses; JJV obtained the funding and donor support for the research; BD trained and managed the survey teams in Bihar; JJV, WH, CJ interpreted the data; CJ responsible for data curation; all authors wrote and reviewed the paper.

COMPETING INTERESTS

The authors have no competing interests.

FUNDING

This research was funded by the Bill and Melinda Gates Foundation Investment ID OPP1142889

DATA SHARING STATEMENT

Data are available upon reasonable request.

REFERENCES

1. UNSTATS. Official list of MDG indicators: United Nations Statistics Division, Department of Economic and Social Affairs; 2005 [Available from: https://unstats.un.org/unsd/mdg/Host.aspx?Content=Indicators/OfficialList.htm accessed 20 June 2018.
2. UN DESA. Sustainable Development Goals: Division for Sustainable Development Goals, United Nations Department of Economic and Social Affairs; 2015 [Available from: https://www.un.org/sustainabledevelopment/sustainable-development-goals/ accessed 1 August 2018.
3. International Institute for Population Sciences (IIPS) and ICF. National Family Health Survey (NFHS-4), 2015-16: India. Mumbai: IIPS 2017.
4. MOSPI. Millennium Development Goals India Country Report 2015. New Delhi: Social Statistics Division, Central Statistics Office, Ministry of Statistics and Programme Implementation 2015.
5. Karvande S, Sonawane D, Chavan S, et al. What does quality of care mean for maternal health providers from two vulnerable states of India? Case study of Bihar and Jharkhand. *Journal of Health, Population and Nutrition* 2016;35(6):1-10. doi: 10.1186/s41043-016-0043-3
6. Mohan P, Kishore B, Singh S, et al. Assessment of implementation of integrated management of neonatal and childhood illness in India. *Journal of Health, Population and Nutrition* 2011;29(6):639-38.

For peer review only - http://bmjopen.bmj.com/site/about/guidelines.xhtml

7. Neogi SB, Sharma J, Chauhan M, et al. Care of newborn in the community and at home. *Journal of Perinatology* 2016;36(Suppl 3):s13-7. doi: 10.1038/jp.2016.185
8. CARE Family Health. Integrated Family Health Initiative: Catalysing change for healthy communities, 2013.
9. Blanc AK, Warren CE, McCarthy KJ, et al. Assessing the validity of indicators of the quality of maternal and newborn health care in Kenya. *Journal of Global Health* 2016;6(1):1-11.
10. Eisele TP, Rhoda DA, Cutts FT, et al. Measuring coverage in MNCH: Total survey error and the interpretation of intervention coverage estimates from household surveys. *PLoS Medicine* 2013;10(5):e1001386. doi: 10.1371/journal.pmed.1001386
11. Anoke SC, Mwai P, Jeffery C, et al. Comparing two survey methods of measuring health‐related indicators: Lot Quality Assurance Sampling and Demographic Health Surveys. *Tropical Medicine and International Health* 2015;20(12):1756-70. doi: 10.1111/tmi.12605
12. Valadez JJ, Devkota B, Pradhan MM, et al. Improving malaria treatment and prevention in India by aiding district managers to manage their programmes with local information: a trial assessing the impact of Lot Quality Assurance Sampling on programme outcomes. *Trop Med Int Health* 2014 doi: 10.1111/tmi.12354
13. Tourangeau R. Remembering what happened: Memory errors and survey reports. In: Stone AA, Jobe JB, Bachrach CA, et al., eds. The Science of Self-report : Implications for research and practice. Mahwah, NJ: Lawrence Erlbaum 2000.
14. Bradburn NM. Temporal representation and event dating. In: Stone AA, Jobe JB, Bachrach CA, et al., eds. The Science of Self-report : Implications for research and practice. Mahwah, NJ: Lawrence Erlbaum 2000.
15. Tomeo CA, Rich-Edwards JW, Michels KB, et al. Reproducibility and validity of maternal recall of pregnancy-related events. *Epidemiology* 1999;10(6):774-7.
16. Elkadry E, Kenton K, White P, et al. Do mothers remember key events during labor? *American Journal of Obstetrics and Gynecology* 2003;189(1):195-200. doi: 10.1067/mob.2003.371
17. Li R, Scanlon KS, Serdula MK. The validity and reliability of maternal recall of breastfeeding practice. *Nutrition Reviews* 2005;63(4):103-10. doi: 10.1301/nr.2005.apr.103-110
18. Miles M, Ryman TK, Dietz V, et al. Validity of vaccination cards and parental recall to estimate vaccination coverage: A systematic review of the literature. *Vaccine* 2013;31:1560-8. doi: 10.1016/j.vaccine.2012.10.089
19. Valadez JJ, Weld LH. Maternal recall error of child vaccination status in a developing nation. *Am J Public Health* 1992;82(1):120-2. [published Online First: 1992/01/01]
20. Chan M, Kazatchkine M, Lob-Levyt J, et al. Meeting the demand for results and accountability: a call for action on health data from eight global health

For peer review only

agencies. *PLoS Med* 2010;7(1):e1000223. doi: 10.1371/journal.pmed.1000223 [published Online First: 2010/02/04]

21. Valadez JJ, Weiss W, Leburg C, et al. Assessing Community Health Programs: A Trainer's Guide. Using LQAS for Baseline Surveys and Regular Monitoring. 2nd ed. St Albans: Teaching-aids At Low Cost 2007.
22. Valadez JJ. Assessing Child survival Programs in Developing Countries: Testing Lot Quality Assurance Sampling. Boston: Harvard School of Pblic Health 1991.
23. Robertson SE, Valadez JJ. Global review of health care surveys using lot quality assurance sampling (LQAS), 1984–2004. *Soc Sci Med* 2006;63(6):1648-60. doi: 10.1016/j.socscimed.2006.04.011
24. Turner AG, Magnani RJ, Shuaib M. A not quite as quick but much cleaner alternative to the Expanded Programme on Immunization (EPI) Cluster Survey design. *International journal of epidemiology* 1996;25(4):198-203. doi: 10.1093/ije/25.1.198
25. Davis RH, Valadez JJ. Improving the collection of knowledge, attitude and practice data with community surveys: a comparison of two second-stage sampling methods. *Health Policy Planning* 2014;29(8):1054-60. doi: 10.1093/heapol/czt088
26. Mantel N. Chi-square tests with one degree of freedom: extensions of the Mantel-Haenszel procedure. *J Am Stat Assoc* 1963;58(303):690-700.
27. Agresti A. An Introduction to Categorical Data Analysis. 2nd ed. Hoboken, New Jersey: Wiley-Interscience; John Wiley & Sons 2007.
28. World Weather Online. Patna Monthly Climate Averages, Bihar, India 2018 [Available from: https://www.worldweatheronline.com/patna-weather-averages/bihar/in.aspx accessed August 15 2018.

For peer review only - http://bmjopen.bmj.com/site/about/guidelines.xhtml

Table S1. Subject domain and indicators labels

Domain[a]	Indicator	Indicator text
1	1	Proportion of mothers of infants (0-2/0-5) months who were registered during their last pregnancy
1	2	Proportion of mothers of infants (0-2/0-5) months whose last pregnancy was registered in the first three months of pregnancy
1	3	Proportion of mothers of infants (0-2/0-5) months attended at least one ANC visit during their last pregnancy
1	4	Proportion of mothers of infants (0-2/0-5) months attended 3 or more ANC visits during their last pregnancy
1	5	Proportion of mothers of infants (0-2/0-5) months attended 4 or more ANC visits during their last pregnancy
1	6	Proportion of mothers of infants (0-2/0-5) months who attended at least one ANC visit where BP was checked during her last pregnancy
1	7	Proportion of mothers of infants (0-2/0-5) months who attended at least one ANC where at least one sonography was performed during her last pregnancy
1	8	Proportion of mothers of infants (0-2/0-5) months who attended at least one ANC where at least one abdominal examination was performed during her last pregnancy
1	9	Proportion of mothers of infants (0-2/0-5) months who attended at least one ANC where at least one urine test was performed during her last pregnancy
1	10	Proportion of mothers of infants (0-2/0-5) months who attended at least one ANC where at least one blood test was performed during her last pregnancy
1	40	Proportion of mothers of infants (0-2/0-5) months who have used an ambulance to reach an institution for last delivery
2	11	Proportion of mothers of infants (0-2/0-5) months who were protected against tetanus in their last pregnancy (Neonatal TT)
2	12	Proportion of mothers of infants (0-2/0-5) months who received two or more doses of TT injection in their last pregnancy
2	13	Proportion of mothers of infants (0-2/0-5) months who received IFA for 100 days or more during their pregnancy
2	14	Proportion of mothers of infants (0-2/0-5) months consumed IFA for 100 days or more in their pregnancy
3	15	Proportion of mothers (home + institutional delivery) of infants (0-2/0-5) months who planned transportation to health facility in their last pregnancy
3	16	Proportion of mothers (home + institutional delivery) of infants (0-2/0-5) months who have identified anybody who would donate blood in the case of emergency in their last pregnancy

For peer review only

3	17	Proportion of mothers (home + institutional delivery) of infants (0-2/0-5) months who have identified persons who would take care of the baby immediately after birth
3	18	Proportion of mothers (home + institutional delivery) of infants (0-2/0-5) months who have arranged new blade & thread for their last delivery
3	19	Proportion of mothers who planned for institutional delivery of infants (0-2/0-5) months who arranged new blade & thread for their delivery
3	20	Proportion of mothers (home + institutional delivery) of infants (0-2/0-5) months who have arranged clean cloth for mothers and baby
3	21	Proportion of mothers who planned institutional delivery of infants (0-2/0-5) months who arranged clean cloth for mothers and baby
3	22	Proportion of mothers of infants (0-2/0-5) months who have saved money for the delivery
3	23	Proportion of mothers who planned for institutional delivery of infants (0-2/0-5) months identified person to accompany her during the delivery
4	24	Proportion of mothers of infants (0-2/0-5) months who were visited by ASHA at least once during their last pregnancy
4	25	Proportion of mothers of infants (0-2/0-5) months who were visited by AWW at least once during their last pregnancy
4	26	Proportion of mothers of infants (0-2/0-5) months who were visited by FLWs at least once during their last pregnancy
4	27	Proportion of mothers of infants (0-2/0-5) months who were visited by ASHAS at least once during their last pregnancy
4	28	Proportion of mothers of infants (0-2/0-5) months who were visited by AWW at least once during their last pregnancy
4	29	Proportion of mothers of infants (0-2/0-5) months who were visited by any FLW in the last trimester during their last pregnancy
4	30	Proportion of mothers of infants (0-2/0-5) months who were visited 2 or more times by any FLW in the last trimester during their last pregnancy
4	31	Proportion of mothers of infants (0-2/0-5) months who were visited home by ASHA within 24 hours of last delivery
4	32	Proportion of mothers of infants (0-2/0-5) months who were visited home by AWW within 24 hours of last delivery
4	33	Proportion of mothers of infants (0-2/0-5) months who were visited home by any FLW within 24 hours of last delivery
4	34	Proportion of mothers of infants (0-2/0-5) months who were visited home by any AWW within the first week of last delivery

For peer review only

4	35	Proportion of mothers of infants (0-2/0-5) months who were visited home by any FLW within first week of last delivery
5	37	Proportion of mothers of infants (0-2/0-5) months whose last child was delivered at a health facility (private or public facility)
5	38	Proportion of mothers of infants (0-2/0-5) months whose last child was delivered at public facility
5	39	Proportion of mothers (home delivery) of infants (0-2/0-5) months who had home delivery attended by skilled birth attendant (SBA)
5	39.5	Proportion of mothers (home + institutional delivery) of infants (0-2/0-5) months who had home delivery attended by skill birth attendant (SBA)
6	42	Proportion of infants (home + institutional delivery) aged (0-2/0-5) months with nothing applied to the umbilical cord after cutting and tying in their last delivery
7	43	Proportion of mothers (home + institutional delivery) of infants (0-2/0-5) months who have delivered baby practiced skin to skin care (STSC) immediately after birth
7	44	Proportion of mothers with institutional delivery of infants (0-2/0-5) months who continued skin to skin care (STSC) at home
7	45	Proportion of mothers with home delivery of infants (0-2/0-5) months who continued skin to skin care (STSC) later
7	46	Proportion of mothers of infants (0-2/0-5) months who were breastfed within one hour of birth
7	47	Proportion of infants aged (0-2/0-5) months who have received delayed bath (between 48 hours and before 7 days after birth) (Public facility/Private facility/Home)
7	48	Proportion of infants aged (0-2/0-5) months who were weighed after birth (Public facility/Private facility/Home)
7	49	Proportion of infants aged (0-2/0-5) months who were delivered at HF received dry cord care
7	50	Proportion of infants aged (0-2/0-5) months who were delivered at HF continued with dry cord care
7	51	Proportion of infants aged (0-2/0-5) months who were delivered at home continued with dry cord care
8	52	Proportion of infants (0-2/0-5) months who were breast-fed in the past 24 hours (Exclusively Breast-fed)

a. 1 ANC & birth preparedness, 2 Maternal health, 3 Birth preparedness, 4 Front Line Worker Support, 5 Birth history & newborn care, 6 Dry cord, 7 Neonatal health, 8 Exclusive Breastfeeding

For peer review only

BMJ Open

How well do mothers recall their own and their infant's perinatal events? a two-district study using cross-sectional stratified random sampling in Bihar, India

Journal:	*BMJ Open*
Manuscript ID	bmjopen-2019-031289.R1
Article Type:	Research
Date Submitted by the Author:	05-Aug-2019
Complete List of Authors:	Valadez, Joseph; Liverpool School of Tropical Medicine, International Public Health<br>Devkota, Baburam; Liverpool School of Tropical Medicine, International Public Health<br>Jeffery, Caroline; Liverpool School of Tropical Medicine<br>Hadden, Wilbur; University of Maryland at College Park, Sociology
**Primary Subject Heading**:	Global health
Secondary Subject Heading:	Epidemiology, Global health, Public health, Research methods
Keywords:	Public health < INFECTIOUS DISEASES, maternal recall, Bihar, India, perinatal, surveys, LQAS

SCHOLARONE™
Manuscripts

How well do mothers recall their own and their infant's perinatal events? a two-district study using cross-sectional stratified random sampling in Bihar, India

Joseph J Valadez[1*], Baburam Devkota[1], Caroline Jeffery[1], Wilbur C. Hadden[2]

[1] Department of International Public Health, Liverpool School of Tropical Medicine, Liverpool, United Kingdom

[2] Department of Sociology, Center for Innovation, University of Maryland, USA

* Corresponding author: Professor Joseph J Valadez, Department of International Public Health, Liverpool School of Tropical Medicine, Pembroke Place, Liverpool, L3 5QA United Kingdom. Email: joseph.valadez@lstmed.ac.uk; Mobile: +44(0)754 8523717

Manuscript word length: 2255

For peer review only

For peer review only - http://bmjopen.bmj.com/site/about/guidelines.xhtml

Abstract

INTRODUCTION

Global monitoring of maternal, newborn and child health (MNCH) programmes use self-reported data subject to recall error which leads to incorrect decisions for improving health services and wasted resources. To minimize this risk, samples of mothers of infants aged 0-2 and 3-5 months are sometimes used. We report a test of whether a single sample of mothers of infants 0-5 months provides the same information.

METHODS

An annual MNCH household survey in two districts of Bihar, India (N=6million), collected data from independent samples of mothers of infants 0-5, 0-2 and 3-5 months. Analyses compare responses from the first two samples (n=950) to assess recall bias using 51 indicators with Mantel-Haenszel-Cochran statistics.

RESULTS

No measurable differences are detected in 79.4% of comparisons while 20.6% display differences. A difference detected for exclusive breastfeeding is due to early commencement of complementary-feeding by older infants. There are measurable differences in 33% of the indicators on front line worker (FLW) support, 25% of the indicators

For peer review only

of birth preparedness, 28% for place of birth and the birth attendant, 15% for neonatal care, 5% for antenatal care, and 0% for maternal health. Differences in FLW visits and compliance with their advice may be due to seasonal effects: mothers of older infants 3-5 months were pregnant during the dry season; mothers of children 0-2 months were pregnant during the monsoons, making transportation difficult.

CONCLUSIONS

Useful coverage estimates can be obtained by sampling mothers with infants 0-5 months as with two cohorts: 0-2 and 3-5 months suggesting that mothers of young infants recall their own perinatal events and those of their children, but for some indicators, for example exclusive breast feeding, it may be necessary to adjust targets. Excessive stratification wastes resources, does not improve the quality of information, and increases the burden placed on data collectors and communities. This burden can increase non-sampling error.

STRENGTHS AND LIMITATIONS OF THIS STUDY

- Strength: The data were produced using stratified random sampling with no apparent design effect leading to an efficient use of information.
- Strength: Data were collected from female participants by female data collectors which is likely to have reduced non-sampling error.

For peer review only - http://bmjopen.bmj.com/site/about/guidelines.xhtml

- Strength: The study population is very large covering a large geographical area, reducing the likelihood that the results are pertinent only to a small group of mothers with infants; results may be generalizable.
- Strength: Both weighted and unweighted results are presented giving strength to the conclusions.
- Limitation: The study is confined to assessing data in the 0-2 month cohort vis a vis a 0-5 month cohort of infants; journal space limitations prevent expanding the research to assess indicators from the 3-5 month cohort vis a vis the 0-5 month cohort.

Key Words:

Maternal recall, surveys, maternal health, MNCH, LQAS, Bihar, India

INTRODUCTION

The progress toward United Nations' Sustainable Development Goal (SDG) 3 is measured with 9 targets, including the Maternal Mortality Ratio (MMR) and under 5 mortality rate (U5MR)[1 2]. In India, Bihar is one of the largest (population 110 million) and poorest (53% of households are in the lowest wealth index quintile of India[3]) states with high child and maternal mortality ratio (U5MR=54, MMR=208)[4] (see[5] for an evaluation of the health

For peer review only - http://bmjopen.bmj.com/site/about/guidelines.xhtml

care system in Bihar), and is a priority for donor support for health systems strengthening.

To accelerate progress toward achieving SDG 3, state governments in India pursue programs of community-based care (see[6 7] for descriptions and assessments of this approach). Since 2011, the Bihar Ministry of Health has supported an Integrated Family Health Initiative to improve the availability, quality and use of prenatal, perinatal and postnatal care for mothers and infants[8].

The usual way to monitor progress toward achieving these goals is with household surveys. Perhaps the most commonly used surveys are cluster sample surveys such as the Demographic and Health Surveys and the Multiple Indicator Cluster Surveys[9 10]. An alternative design is Lot Quality Assurance Sampling (LQAS), which provides comparable data but is decentralized to local health service organisations and more useful for management and program planning[11]. Several states in India find it benefits their programs[12]. Surveys rely on the reports of mothers of infants and young children, but these reports are subject to several sources of potential error and bias through interviewees not knowing, forgetting, and having memory errors[13 14]. Studies have shown both that mothers can accurately report significant facts about the birth and

For peer review only

For peer review only - http://bmjopen.bmj.com/site/about/guidelines.xhtml

care of their children many years after the event[15], but also that even immediately after giving birth mothers may misreport details[16-18]. Studies of maternal recall of her child's vaccination status concluded that due to offsetting errors of maternal reports, the resulting data accurately measured vaccination rates[19]; the pattern of error revealed that mothers whose children are up-to-date or nearly so tended to underestimate their child's vaccination status whilst mothers whose children have few vaccinations, overestimate their coverage.

To improve the validity of collected data, knowledge, practice and coverage surveys have used dedicated samples of mothers of infants 0-11 months of age or 0-5 months of age and children 6-11 months of age. In Bihar, local organisations departed from this convention of sampling two cohorts of children and have been monitoring their programs' progress by sampling five dedicated cohorts: mothers of children 0-2, 3-5, 6-8, 9-11 and 12-23 months old with indicators focused on antenatal care, safe delivery practices, infant and young child feeding practices, immunisation, treatment seeking, and more. To avoid the possibility of maternal recall error, each of the five cohorts was asked questions particularly relevant to a child's specific age group.

For peer review only

In countries such as India with a high maternal ratio and child mortality rate, regular monitoring of related health service coverage is critical to reducing mortality. However, the survey designs should be affordable and sustainable for local health systems; they should also produce precise, unbiased estimates[20]. In this study, we explore whether information is gained by sampling cohorts of children aged 0-2 and 3-5 months or whether the sample size can be reduced by 50% by creating one sample cohort aged 0-5 months.

The research question we address is: "Do the health service delivery coverage estimates from a sample of mothers of infants aged 0-5 months differ from those obtained from a sample of mothers of infants ages 0-2 months?" We compare district coverage estimates obtained from two independent samples of infants 0-2 months and 0-5 months of age. The implications of this study are important for health systems researchers needing results to appraise and improve their programmes.

METHODS

To answer the research question, we collected information from a sample of mothers with infants 0-5 months old and a sample of mothers with 0-2 month old

For peer review only

infants in two districts. This study took place within the context of a larger survey that also sampled children 3-5, 6-8, 9-11 and 12-23 months of age. However, we only use the two aforementioned groups to assess the measurement of the indicators and refer only to them for the remainder of this paper. We do not assess differences between the 3-5 month and 0-5 month samples as different indicators are measured in the two age group questionnaires with the exception of institutional delivery (indicators #37, #38, and exclusive breastfeeding). The household sampling design we used is a stratified random sample. Within each district, the strata are administrative units of the health system, which in Bihar is called a *block*. Within each block the primary sampling unit is the Anganwadi Centre (Community Health Subcentre) Catchment Area (ACCA). From each ACCA one respondent is randomly selected from each age-group under study following the standard procedure[21]. There are 14 and 11 blocks in Gopalganj and Aurangabad (N=6 million), the two districts selected for this study, respectively. The sampling design used lot quality assurance sampling (LQAS) approaches. A commonly used random sample size of 19 individuals in each target group was selected in each block using probability proportional to size[22 23].

For peer review only - http://bmjopen.bmj.com/site/about/guidelines.xhtml

Within each sampling location (typically a village) one person from each target group was randomly sampled using segmentation sampling[24 25]. The total sample sizes are: (a) Gopalganj: 19 x 14 blocks= 266 infants 0-2 months and 266 infants 0-5 months, and (b) Aurangabad: 19 x 11 blocks= 209 infants 0-2 months and 209 infants 0-5 months. The 0-5 month old sample is 60% 0-2 months old and 40% 3-5 months old.

Using summary data from each of the two samples we analyse the data with Cochran-Mantel-Haenszel (CMH)[26] tests for 51 dichotomous indicators (See Supplementary File Table S1) common to the two samples. The CMH tests theoretically have a Chi-square probability distribution with 1 degree of freedom. With a sufficient number of respondents or a sufficient number of blocks, the CMH test is equivalent to conditional logistic regression[27: 114-115]. In this analysis both the number of respondents and the number of blocks only approach sufficiency. Consequently, the calculated chi-squares and probabilities must be considered as approximations of their true values.

We calculate both unweighted and weighted estimates (See Supplementary File Table S2). The unweighted estimates permit the results from smaller blocks to have equal weight vis à vis larger ones. Since the research question concerns

For peer review only - http://bmjopen.bmj.com/site/about/guidelines.xhtml

an analysis of which age cohort is most informative, the weighted estimates may not be as useful as the unweighted ones. However, the weighted estimates provide better point estimates of the indicators at the district level. The effect of the weights on the Chi-square statistics is to increase the contribution of the larger blocks and decrease the contribution of the smaller blocks. Hence, we report both sets of results.

The Chi-square probability distribution puts the differences between the districts on a readily interpretable probability scale (See Supplementary File Table S2). To determine meaningful differences in responses between the two age cohorts we used a probability of 0.05 as a cut-off value and consider differences with probabilities less than 0.05 to be possibly meaningful and those with larger probabilities to be likely due to sampling errors. With 102 comparisons (51 indicators weighted or unweighted) we must expect some to exceed this cut-off by chance alone. If all of the comparisons were independent, we might randomly find 5 differences, but many of the indicators measure related events (e.g., number of ANC visits and tetanus toxoid vaccinations) and the weighted and unweighted estimates are similar, so these indicators are not all independent and it is not possible

For peer review only - http://bmjopen.bmj.com/site/about/guidelines.xhtml

to calculate an expected number of differences nor is it appropriate to interpret these probabilities as a measure of "statistical significance".

Patient and Public Involvement

This study does not involve patients. Also, the public was not involved in the design, conduct and reporting of the research. The public was engaged as interviewees. To ensure local engagement we coordinated with the Bihar Ministry of Health, local implementing non-governmental organizations and our donor. We also shared the results with them and offered further dissemination of results.

RESULTS

We find a high level of agreement between the two samples (Table 1). Out of 102 weighted and unweighted comparisons between the estimates from the 0-2 month and 0-5 month samples there is no probable difference in 81 (79.4%) in both the unweighted and weighted estimates. We detect that probable differences exist for 13 comparisons (12.7%). For the remaining eight comparisons the weighted and unweighted estimates disagree. The weighted estimates find seven differences that the unweighted estimates do not; the unweighted estimates find one difference that the weighted estimates do not find.

For peer review only - http://bmjopen.bmj.com/site/about/guidelines.xhtml

Table 1. Number of indicators by probability of a difference between the 0-2 months and the 0-5 months samples for weighted and unweighted samples

Unweighted	Weighted					
	Aurangabad		Gopalganj		Total	
	>= .05	< .05	>= .05	< .05	>= .05	< .05
>= .05	40	3	41	4	81	7
< .05	0	8	1	5	1	13

For different health service domains, the number of indicator comparisons varies from two (Exclusive Breastfeeding-EBF) to 24 concerning home visits by Front Line Worker (FLW) support (Table 2). The two principal FLW are Anganwadi workers and Accredited Social Health Activists (ASHA).

For peer review only

Table 2. Number of indicator comparisons by subject domain showing a measurable difference using weighted and unweighted estimates of 0-2 months and 0-5 months samples

Health Service Domain	Total comparisons	No measurable difference between 0-2 and 0-5 months results	Measurable difference between 0-2 and 0-5 months results			
			Both	Unweighted only	Weighted only	Percent (%) indicators with different results
Antenatal care	22	21	0	0	1	5
Place of birth & attendant	8	6	1	0	1	25
Birth preparedness	18	13	3	0	2	28
Front Line Worker support	24	16	6	0	2	33
Maternal health	8	8	0	0	0	0
Neonatal care	20	17	1	1	1	15
Exclusive Breastfeeding	2	0	2	0	0	100
Totals	102	81	13	1	7	21.9

For peer review only - http://bmjopen.bmj.com/site/about/guidelines.xhtml
For peer review only

In the FLW support domain 33% of comparisons have probable differences. The neonatal health domain has 20 comparisons and the birth preparedness domain has 18; in these domains 15% and 28% show probable differences, respectively. Place of birth and the birth attendant, and maternal health each have eight comparisons with 25%, or two comparisons, and 0 comparisons, respectively, showing a possible difference. The differences between the two samples cluster around home visits from FLW and behaviours associated with birth preparedness and neonatal care. Details of these differences are listed in Table 3.

For two indicators, both the weighted and unweighted estimates display probable differences between the 0-2 and 0-5 months samples in both districts. For indicator #52, the proportion exclusively breastfeeding, the 0-5 months cohort has the lower estimate, and indicator #24, the proportion of mothers visited by an ASHA at least once during their last pregnancy, the 0-5 months sample gives the higher estimate, about 74%, compared to 63% in the 0-2 months sample.

For peer review only - http://bmjopen.bmj.com/site/about/guidelines.xhtml

Table 3. Indicators by health service domain showing measurement differences between 0-2 months and 0-5 months samples

Health Service Domain and Indicator	Indicator No.	District	Weighted Coverage (%)		p-value		Estimate type
			0-2 months	0-5 months	Unweighted Estimate	Weighted Estimate	
**Antenatal care**							
Proportion of mothers of infants (0-2/0-5 months) registered during their last pregnancy	1	Aurangabad	85.2	77.6	0.0552	0.0365	Weighted
**Place of birth & birth attendant**							
Proportion of mothers of infants (0-2/0-5 months) whose last child was delivered at a public facility	38	Gopalganj	51.2	61.6	0.0363	0.0159	Both
Proportion of mothers of infants (0-2/0-5 months) whose last child was delivered at a health facility (private or public facility)	37	Gopalganj	78.9	85.5	0.0643	0.0459	Weighted
**Birth preparedness**							
Proportion of mothers of infants (0-2/0-5 months) who planned transportation to health facility in their last pregnancy (home & institutional delivery)	15	Gopalganj	45.7	56.0	0.0266	0.0158	Both
Proportion of mothers of infants (0-2/0-5 months) who identified persons to care for the baby immediately after birth (home + institutional delivery)	17	Gopalganj	51.8	62.6	0.0255	0.0103	Both
Proportion of mothers of infants (0-2/0-5 months) who planned for institutional delivery and	23	Aurangabad	62.5	47.0	0.0039	0.0052	Both

For peer review only - http://bmjopen.bmj.com/site/about/guidelines.xhtml

identified person to accompany her during the delivery							
Proportion of mothers who planned for institutional delivery of infants (0-2/0-5 months) who had a new blade & thread for their delivery	19	Aurangabad	23.5	14.5	0.062	0.0429	Weighted
Proportion of mothers who planned institutional delivery of infants (0-2/0-5 months) who arranged clean cloth for mothers and baby	21	Aurangabad	43.6	31.2	0.0546	0.0137	Weighted
**Front Line Worker support**							
Proportion of mothers of infants (0-2/0-5 months) who were visited by ASHA at least once during their last pregnancy	24	Aurangabad	62.2	75.2	0.0023	0.0042	Both
		Gopalganj	63.5	73.0	0.0284	0.0175	Both
Proportion of mothers of infants (0-2/0-5 months) visited at home by FLWs at least once during their last pregnancy	26	Aurangabad	63.5	76.7	0.0021	0.0032	Both
Proportion of mothers of infants (0-2/0-5 months) visited at home by ASHA within 24 hours of last delivery	31	Aurangabad	29.9	44.9	0.0009	0.0016	Both
Proportion of mothers of infants (0-2/0-5 months) visited at home by any FLW within 24 hours of last delivery	33	Aurangabad	32.2	46.7	0.0015	0.0026	Both
Proportion of mothers of infants (0-2/0-5 months) visited at home by any FLW within first week of last delivery	35	Aurangabad	44.5	59.3	0.0018	0.0026	Both
Proportion of mothers of infants (0-2/0-5 months) visited at home by any AWW within the first week of the last delivery	34	Gopalganj	14.4	8.9	0.0959	0.0471	Weighted
Proportion of mothers of infants (0-2/0-5 months) visited by	27	Gopalganj	52.6	61.1	0.0617	0.0449	Weighted

For peer review only - http://bmjopen.bmj.com/site/about/guidelines.xhtml

ASHAs at least once during their last trimester of pregnancy							
**Infant care**							
Proportion of infants aged (0-2/0-5 months) who were delivered at home continued with dry cord care	51	Aurangabad	78.0	45.4	0.0001	0.0006	Both
		Gopalganj	63.7	41.1	0.0431	0.0627	Unweighted
Proportion of infants aged (0-2/0-5 months) weighed after birth (Public facility/Private facility/Home)	48	Gopalganj	70.7	78.2	0.0727	0.0464	Weighted
**Exclusive Breastfeeding**							
Proportion of infants (0-2/0-5 months) breast-fed in the past 24 hours (Exclusively Breast-fed)	52	Aurangabad	69.2	59.7	0.0229	0.0411	Both
		Gopalganj	82.1	68.4	0.0001	0.0003	Both

For peer review only

DISCUSSION

Statement of principal findings

There are no measurable differences in coverage estimates for 79.4% (81 comparisons) of the indicator comparisons between the samples of mothers with infants 0-2 months old versus mothers of infants 0-5 months old; 12.7% (13 comparisons) display measurable differences. The remaining 7.8% (eight comparisons) display discrepancies between the weighted and unweighted estimates.

Strengths and weaknesses of the study

The strengths of this study are that it compares estimates from two independent samples and that there are many estimates from diverse domains. The weaknesses of this study are that the data were collected in only 2 districts of 1 state in India and at a particular point in time. Another weakness was the dataset did not permit comparison of the 0-2 month and 3-5 month age group, and the 3-5 month and 0-5 month age group for the same indicators.

Strengths and weaknesses in relation to other studies

Other studies of maternal recall bias have sought a "gold standard" to represent reality and to evaluate

For peer review only - http://bmjopen.bmj.com/site/about/guidelines.xhtml

measures. Our study, of course, is interested in reality, but this study compares alternative measures needed to assess the Bihar health program. It also uses a complete sample of the age grouping under study rather than just a sub-sample of a larger age grouping. Therefore, no statistical power is lost in this analysis.

Meaning of the study

The evidence indicates that samples of the broader 0-5 month age group yield comparable results and those of the narrower 0-2 month age group. It is not necessary to double the total sample by measuring independently 0-2 months and 3-5 months cohorts of children. These results also tend to dispel the hypothesis that maternal recall is problematic for mothers during the first 6 months following delivery. Our results are more consistent with conclusions presented in earlier research[15] and they support those organizations collecting data with 0-5 month cohorts.

Indicator #52, EBF, displayed two comparisons measuring decreases in both districts. This is not surprising as fewer infants are expected to be exclusively breast fed in a sample ranging from 0 to 5 months than a sample ranging 0 to 2 months; mothers introduce complementary feeding and liquids as infants age despite

For peer review only - http://bmjopen.bmj.com/site/about/guidelines.xhtml

this being a health risk. This difference could be accommodated by adjusting expectations and targets for the indicator.

Unanswered questions and future research

Further investigation and consideration of the differences is warranted. The eight differences found in the FLW support indicators deserve more scrutiny. Seven show higher estimates for the 0-5 cohort and one has a higher estimate for the 0-2 month cohort. The former seven differences may be due to excessive rainfall during the July-September (monthly average 318.95mm, range:195.99-395.8mm) versus the lesser rainfall during June-April (monthly average 25.52mm, range: 0.3-27.88mm) which in the last trimester may have reduced the access of ASHA in the 0-2 month cohort [28]. Indicators such as these may be particularly sensitive to rainfall and may explain why more mothers in the 0-5 month cohort displayed higher FLW visitation estimates since FLW were not impeded by the monsoon and the resulting muddy roads.

Differences in birth preparedness and institutional birth may be a consequence of the differences in rainfall or in FLW support with increases a response to the need for more careful planning when transportation is difficult and

For peer review only - http://bmjopen.bmj.com/site/about/guidelines.xhtml

decreases due to a reduction in the educational efforts of the FLW. Or, some of these differences may just be due to noise in the data.

CONCLUSIONS

Overall, the answer to the research question, "Can one get the same district coverage estimates from a sample of mothers of infants aged 0-5 months as from a sample of mothers of infants ages 0-2 months?" is yes. This result can be paraphrased as: mothers do not display increased recall errors of their perinatal health care behaviour in a cohort of mothers with infants 0-5 months as compared with mothers with younger infants. Substantial resources and effort can be saved using a survey design that avoids needless expenses to collect data that provides insubstantial amounts of information. It also reduces the burden on data collectors and community participants. Fatigue to both groups can result in needless non-sampling error.

ACKNOWLEDGMENTS

We gratefully acknowledge the essential roles of Hemant Das, Alok Prahdan and Sanjay Biswa for their careful field work supporting the LQAS survey and the HMIS data retrieval. We thank Prof Imelda Bates, Prof Brian Faragher and Nancy

For peer review only - http://bmjopen.bmj.com/site/about/guidelines.xhtml

Vollmer for their valuable feedback on an earlier version of this manuscript. This work was supported by the Bill and Melinda Gates Foundation (Investment ID OPP1142889).

ETHICS

The Ethical Committees of the authors' institutes approved the protocol, study instruments and consent procedures for the survey data collection.

COMPETING INTERESTS

The authors have read the statement on competing interests and declare that they have none.

AUTHORS' CONTRIBUTIONS

JJV, BD developed the research question and survey design; WH, CJ carried out the statistical analyses; JJV obtained the funding and donor support for the research; BD trained and managed the survey teams in Bihar; JJV, WH, CJ interpreted the data; CJ responsible for data curation; all authors wrote and reviewed the paper.

COMPETING INTERESTS

The authors have no competing interests.

FUNDING

This research was funded by the Bill and Melinda Gates Foundation Investment ID OPP1142889

DATA SHARING STATEMENT

Data are available upon reasonable request.

REFERENCES

1. UNSTATS. Official list of MDG indicators: United Nations Statistics Division, Department of Economic and Social Affairs; 2005 [Available from: https://unstats.un.org/unsd/mdg/Host.aspx?Content=Indicators/OfficialList.htm accessed 20 June 2018.

For peer review only - http://bmjopen.bmj.com/site/about/guidelines.xhtml

2. UN DESA. Sustainable Development Goals: Division for Sustainable Development Goals, United Nations Department of Economic and Social Affairs; 2015 [Available from: https://www.un.org/sustainabledevelopment/sustainable-development-goals/ accessed 1 August 2018.
3. International Institute for Population Sciences (IIPS) and ICF. National Family Health Survey (NFHS-4), 2015-16: India. Mumbai: IIPS 2017.
4. MOSPI. Millennium Development Goals India Country Report 2015. New Delhi: Social Statistics Division, Central Statistics Office, Ministry of Statistics and Programme Implementation 2015.
5. Karvande S, Sonawane D, Chavan S, et al. What does quality of care mean for maternal health providers from two vulnerable states of India? Case study of Bihar and Jharkhand. *Journal of Health, Population and Nutrition* 2016;35(6):1-10. doi: 10.1186/s41043-016-0043-3
6. Mohan P, Kishore B, Singh S, et al. Assessment of implementation of integrated management of neonatal and childhood illness in India. *Journal of Health, Population and Nutrition* 2011;29(6):639-38.
7. Neogi SB, Sharma J, Chauhan M, et al. Care of newborn in the community and at home. *Journal of Perinatology* 2016;36(Suppl 3):s13-7. doi: 10.1038/jp.2016.185
8. CARE Family Health. Integrated Family Health Initiative: Catalysing change for healthy communities, 2013.
9. Blanc AK, Warren CE, McCarthy KJ, et al. Assessing the validity of indicators of the quality of maternal and newborn health care in Kenya. *Journal of Global Health* 2016;6(1):1-11.
10. Eisele TP, Rhoda DA, Cutts FT, et al. Measuring coverage in MNCH: Total survey error and the interpretation of intervention coverage estimates from household surveys. *PLoS Medicine* 2013;10(5):e1001386. doi: 10.1371/journal.pmed.1001386
11. Anoke SC, Mwai P, Jeffery C, et al. Comparing two survey methods of measuring health - related indicators: Lot Quality Assurance Sampling and Demographic Health Surveys. *Tropical Medicine and International Health* 2015;20(12):1756-70. doi: 10.1111/tmi.12605
12. Valadez JJ, Devkota B, Pradhan MM, et al. Improving malaria treatment and prevention in India by aiding district managers to manage their programmes with local information: a trial assessing the impact of Lot Quality Assurance Sampling on programme outcomes. *Trop Med Int Health* 2014 doi: 10.1111/tmi.12354
13. Tourangeau R. Remembering what happened: Memory errors and survey reports. In: Stone AA, Jobe JB, Bachrach CA, et al., eds. The Science of Self-report : Implications for research and practice. Mahwah, NJ: Lawrence Erlbaum 2000.
14. Bradburn NM. Temporal representation and event dating. In: Stone AA, Jobe JB, Bachrach CA, et al., eds. The Science of Self-report : Implications for research and practice. Mahwah, NJ: Lawrence Erlbaum 2000.

For peer review only - http://bmjopen.bmj.com/site/about/guidelines.xhtml

15. Tomeo CA, Rich-Edwards JW, Michels KB, et al. Reproducibility and validity of maternal recall of pregnancy-related events. *Epidemiology* 1999;10(6):774-7.
16. Elkadry E, Kenton K, White P, et al. Do mothers remember key events during labor? *American Journal of Obstetrics and Gynecology* 2003;189(1):195-200. doi: 10.1067/mob.2003.371
17. Li R, Scanlon KS, Serdula MK. The validity and reliability of maternal recall of breastfeeding practice. *Nutrition Reviews* 2005;63(4):103-10. doi: 10.1301/nr.2005.apr.103-110
18. Miles M, Ryman TK, Dietz V, et al. Validity of vaccination cards and parental recall to estimate vaccination coverage: A systematic review of the literature. *Vaccine* 2013;31:1560-8. doi: 10.1016/j.vaccine.2012.10.089
19. Valadez JJ, Weld LH. Maternal recall error of child vaccination status in a developing nation. *Am J Public Health* 1992;82(1):120-2. [published Online First: 1992/01/01]
20. Chan M, Kazatchkine M, Lob-Levyt J, et al. Meeting the demand for results and accountability: a call for action on health data from eight global health agencies. *PLoS Med* 2010;7(1):e1000223. doi: 10.1371/journal.pmed.1000223 [published Online First: 2010/02/04]
21. Valadez JJ, Weiss W, Leburg C, et al. Assessing Community Health Programs: A Trainer's Guide. Using LQAS for Baseline Surveys and Regular Monitoring. 2nd ed. St Albans: Teaching-aids At Low Cost 2007.
22. Valadez JJ. Assessing Child survival Programs in Developing Countries: Testing Lot Quality Assurance Sampling. Boston: Harvard School of Pblic Health 1991.
23. Robertson SE, Valadez JJ. Global review of health care surveys using lot quality assurance sampling (LQAS), 1984–2004. *Soc Sci Med* 2006;63(6):1648-60. doi: 10.1016/j.socscimed.2006.04.011
24. Turner AG, Magnani RJ, Shuaib M. A not quite as quick but much cleaner alternative to the Expanded Programme on Immunization (EPI) Cluster Survey design. *International journal of epidemiology* 1996;25(4):198-203. doi: 10.1093/ije/25.1.198
25. Davis RH, Valadez JJ. Improving the collection of knowledge, attitude and practice data with community surveys: a comparison of two second-stage sampling methods. *Health Policy Planning* 2014;29(8):1054-60. doi: 10.1093/heapol/czt088
26. Mantel N. Chi-square tests with one degree of freedom: extensions of the Mantel-Haenszel procedure. *J Am Stat Assoc* 1963;58(303):690-700.
27. Agresti A. An Introduction to Categorical Data Analysis. 2nd ed. Hoboken, New Jersey: Wiley-Interscience; John Wiley & Sons 2007.
28. World Weather Online. Patna Monthly Climate Averages, Bihar, India 2018 [Available from: https://www.worldweatheronline.com/patna-weather-averages/bihar/in.aspx accessed August 15 2018.

For peer review only

Table S1. Subject domain and indicators labels

Domain[a]	Indicator	Indicator text
1	1	Proportion of mothers of infants (0-2/0-5) months who were registered during their last pregnancy
1	2	Proportion of mothers of infants (0-2/0-5) months whose last pregnancy was registered in the first three months of pregnancy
1	3	Proportion of mothers of infants (0-2/0-5) months attended at least one ANC visit during their last pregnancy
1	4	Proportion of mothers of infants (0-2/0-5) months attended 3 or more ANC visits during their last pregnancy
1	5	Proportion of mothers of infants (0-2/0-5) months attended 4 or more ANC visits during their last pregnancy
1	6	Proportion of mothers of infants (0-2/0-5) months who attended at least one ANC visit where BP was checked during her last pregnancy
1	7	Proportion of mothers of infants (0-2/0-5) months who attended at least one ANC where at least one sonography was performed during her last pregnancy
1	8	Proportion of mothers of infants (0-2/0-5) months who attended at least one ANC where at least one abdominal examination was performed during her last pregnancy
1	9	Proportion of mothers of infants (0-2/0-5) months who attended at least one ANC where at least one urine test was performed during her last pregnancy
1	10	Proportion of mothers of infants (0-2/0-5) months who attended at least one ANC where at least one blood test was performed during her last pregnancy
1	40	Proportion of mothers of infants (0-2/0-5) months who have used an ambulance to reach an institution for last delivery
2	11	Proportion of mothers of infants (0-2/0-5) months who were protected against tetanus in their last pregnancy (Neonatal TT)
2	12	Proportion of mothers of infants (0-2/0-5) months who received two or more doses of TT injection in their last pregnancy
2	13	Proportion of mothers of infants (0-2/0-5) months who received IFA for 100 days or more during their pregnancy
2	14	Proportion of mothers of infants (0-2/0-5) months consumed IFA for 100 days or more in their pregnancy
3	15	Proportion of mothers (home + institutional delivery) of infants (0-2/0-5) months who planned transportation to health facility in their last pregnancy
3	16	Proportion of mothers (home + institutional delivery) of infants (0-2/0-5) months who have identified anybody who would donate blood in the case of emergency in their last pregnancy

For peer review only

3	17	Proportion of mothers (home + institutional delivery) of infants (0-2/0-5) months who have identified persons who would take care of the baby immediately after birth
3	18	Proportion of mothers (home + institutional delivery) of infants (0-2/0-5) months who have arranged new blade & thread for their last delivery
3	19	Proportion of mothers who planned for institutional delivery of infants (0-2/0-5) months who arranged new blade & thread for their delivery
3	20	Proportion of mothers (home + institutional delivery) of infants (0-2/0-5) months who have arranged clean cloth for mothers and baby
3	21	Proportion of mothers who planned institutional delivery of infants (0-2/0-5) months who arranged clean cloth for mothers and baby
3	22	Proportion of mothers of infants (0-2/0-5) months who have saved money for the delivery
3	23	Proportion of mothers who planned for institutional delivery of infants (0-2/0-5) months identified person to accompany her during the delivery
4	24	Proportion of mothers of infants (0-2/0-5) months who were visited by ASHA at least once during their last pregnancy
4	25	Proportion of mothers of infants (0-2/0-5) months who were visited by AWW at least once during their last pregnancy
4	26	Proportion of mothers of infants (0-2/0-5) months who were visited by FLWs at least once during their last pregnancy
4	27	Proportion of mothers of infants (0-2/0-5 months) visited by ASHAs at least once during their last trimester of pregnancy
4	28	Proportion of mothers of infants (0-2/0-5) months who were visited by AWW at least once during their last pregnancy
4	29	Proportion of mothers of infants (0-2/0-5) months who were visited by any FLW in the last trimester during their last pregnancy
4	30	Proportion of mothers of infants (0-2/0-5) months who were visited 2 or more times by any FLW in the last trimester during their last pregnancy
4	31	Proportion of mothers of infants (0-2/0-5) months who were visited home by ASHA within 24 hours of last delivery
4	32	Proportion of mothers of infants (0-2/0-5) months who were visited home by AWW within 24 hours of last delivery
4	33	Proportion of mothers of infants (0-2/0-5) months who were visited home by any FLW within 24 hours of last delivery
4	34	Proportion of mothers of infants (0-2/0-5) months who were visited home by any AWW within the first week of last delivery

For peer review only

4	35	Proportion of mothers of infants (0-2/0-5) months who were visited home by any FLW within first week of last delivery
5	37	Proportion of mothers of infants (0-2/0-5) months whose last child was delivered at a health facility (private or public facility)
5	38	Proportion of mothers of infants (0-2/0-5) months whose last child was delivered at public facility
5	39	Proportion of mothers (home delivery) of infants (0-2/0-5) months who had home delivery attended by skilled birth attendant (SBA)
5	39.5	Proportion of mothers (home + institutional delivery) of infants (0-2/0-5) months who had home delivery attended by skill birth attendant (SBA)
6	42	Proportion of infants (home + institutional delivery) aged (0-2/0-5) months with nothing applied to the umbilical cord after cutting and tying in their last delivery
6	43	Proportion of mothers (home + institutional delivery) of infants (0-2/0-5) months who have delivered baby practiced skin to skin care (STSC) immediately after birth
6	44	Proportion of mothers with institutional delivery of infants (0-2/0-5) months who continued skin to skin care (STSC) at home
6	45	Proportion of mothers with home delivery of infants (0-2/0-5) months who continued skin to skin care (STSC) later
6	46	Proportion of mothers of infants (0-2/0-5) months who were breastfed within one hour of birth
6	47	Proportion of infants aged (0-2/0-5) months who have received delayed bath (between 48 hours and before 7 days after birth) (Public facility/Private facility/Home)
6	48	Proportion of infants aged (0-2/0-5) months who were weighed after birth (Public facility/Private facility/Home)
6	49	Proportion of infants aged (0-2/0-5) months who were delivered at HF received dry cord care
6	50	Proportion of infants aged (0-2/0-5) months who were delivered at HF continued with dry cord care
6	51	Proportion of infants aged (0-2/0-5) months who were delivered at home continued with dry cord care
7	52	Proportion of infants (0-2/0-5) months who were breast-fed in the past 24 hours (Exclusively Breast-fed)

a. 1 Antenatal care, 2 Maternal health, 3 Birth preparedness, 4 Front Line Worker Support, 6 Place of birth & attendant, 7 Exclusive Breastfeeding

For peer review only

Table S2. Indicators from mothers in Aurangabad and Gopalganj of infants aged 0-2 months compared to 0-5 months: Estimates, Cochran-Mantel-Haenszel Chi-squares, and probabilities: Bihar 2015										
			Unweighted				Weighted			
Domain[a]	Indicator[b]	District[c]	Percent 0-2	Percent 0-5	Chi-square	Probability	Percent 0-2	Percent 0-5	Chi-square	Probability
1	1	1	86.6	79.9	3.6775	0.0552	85.2	77.6	4.3758	0.0365
1	1	2	85.3	85.3	0.0000	1.0000	85.0	85.4	0.0187	0.8911
1	2	1	44	40.7	0.4996	0.4797	43.0	38.3	1.0147	0.3138
1	2	2	47.7	47.0	0.0304	0.8616	47.5	48.2	0.0212	0.8843
1	3	1	84.7	80.9	1.2398	0.2655	83.8	80.4	0.9592	0.3274
1	3	2	81.2	78.6	0.5985	0.4391	80.6	77.8	0.6424	0.4229
1	4	1	60.3	63.6	0.4931	0.4826	61.1	63.1	0.1792	0.6720
1	4	2	59.4	63.2	0.8252	0.3637	57.4	61.0	0.7211	0.3958
1	5	1	42.1	45.0	0.3491	0.5546	43.2	45.7	0.2561	0.6128
1	5	2	44.7	47.7	0.4866	0.4854	43.4	46.9	0.6302	0.4273
1	6	1	59.8	59.3	0.0107	0.9177	58.9	60.1	0.0644	0.7996
1	6	2	67.7	63.5	1.0487	0.3058	66.3	63.3	0.5176	0.4719
1	7	1	42.6	39.2	0.5043	0.4776	42.0	39.5	0.2824	0.5952
1	7	2	70.3	73.3	0.6025	0.4376	69.2	72.6	0.7537	0.3853
1	8	1	44.0	45.9	0.1685	0.6815	42.7	44.8	0.2028	0.6525
1	8	2	53.4	54.1	0.0322	0.8576	52.4	54.5	0.2438	0.6215
1	9	1	45.9	42.6	0.5004	0.4793	45.8	41.7	0.7523	0.3857
1	9	2	42.9	43.6	0.0321	0.8579	42.8	43.5	0.0231	0.8792
1	10	1	67.0	59.8	2.6555	0.1032	67.2	59.6	2.9924	0.0837
1	10	2	55.3	58.3	0.5191	0.4712	54.8	58.5	0.7674	0.3810
1	40	1	11.5	17.7	3.4797	0.0621	10.6	16.0	2.8329	0.0924
1	40	2	8.6	10.2	0.3686	0.5438	8.5	9.5	0.1374	0.7109
2	11	1	98.6	98.1	0.1451	0.7033	98.5	98.1	0.0844	0.7715
2	11	2	97.0	96.6	0.0614	0.8044	96.9	97.1	0.0131	0.9089
2	12	1	68.4	66.5	0.1763	0.6746	69.3	67.5	0.1626	0.6868
2	12	2	79.7	79.3	0.0116	0.9141	81.9	80.4	0.2143	0.6434
2	13	1	3.8	2.4	0.7384	0.3902	4.0	2.6	0.6021	0.4378
2	13	2	3.4	5.6	1.5745	0.2096	3.0	5.1	1.6016	0.2057

For peer review only

For peer review only - http://bmjopen.bmj.com/site/about/guidelines.xhtml

Table S2. Indicators from mothers in Aurangabad and Gopalganj of infants aged 0-2 months compared to 0-5 months: Estimates, Cochran-Mantel-Haenszel Chi-squares, and probabilities: Bihar 2015 - Continued										
			Unweighted				Weighted			
Domain[a]	Indicator[b]	District[c]	Percent 0-2	Percent 0-5	Chi-square	Probability	Percent 0-2	Percent 0-5	Chi-square	Probability
2	14	1	1.4	1.9	0.1474	0.7010	1.6	2.0	0.1025	0.7489
2	14	2	1.9	3.8	1.7161	0.1902	1.7	3.6	1.8535	0.1734
3	15	1	26.3	34.0	3.1510	0.0759	25.9	33.3	2.9594	0.0854
3	15	2	47.0	56.4	4.9192	0.0266	45.7	56.0	5.8262	0.0158
3	16	1	4.3	3.8	0.0639	0.8004	4.3	3.6	0.1410	0.7073
3	16	2	10.2	13.9	1.8263	0.1766	9.7	14.3	2.8157	0.0933
3	17	1	48.8	56.5	2.5852	0.1079	48.5	55.6	2.2155	0.1366
3	17	2	53.4	62.8	4.9914	0.0255	51.8	62.6	6.5790	0.0103
3	18	1	24.9	22.5	0.3601	0.5485	26.1	23.4	0.4531	0.5009
3	18	2	39.5	45.1	2.2194	0.1363	38.2	43.7	2.1362	0.1439
3	19	1	22.6	14.4	3.4821	0.0620	23.5	14.5	4.0983	0.0429
3	19	2	45.2	40.7	1.1508	0.2834	43.4	39.4	0.6771	0.4106
3	20	1	40.7	37.8	0.4565	0.4993	44.1	39.5	1.1927	0.2748
3	20	2	51.9	57.1	1.8538	0.1733	50.0	55.9	2.3539	0.1250
3	21	1	40.1	30.4	3.6953	0.0546	43.6	31.2	6.0749	0.0137
3	21	2	58.1	52.8	1.7277	0.1887	55.6	51.6	0.8220	0.3646
3	22	1	65.1	59.3	1.5561	0.2122	65.5	59.4	1.7643	0.1841
3	22	2	62.4	68.4	2.2445	0.1341	60.2	68.0	3.6903	0.0547
3	23	1	65.0	49.0	8.3301	0.0039	62.5	47.0	7.8078	0.0052
3	23	2	68.8	63.0	1.8993	0.1682	68.0	62.5	1.5725	0.2098
4	24	1	61.2	75.1	9.2638	0.0023	62.2	75.2	8.2048	0.0042
4	24	2	65.8	74.4	4.8055	0.0284	63.5	73.0	5.6444	0.0175
4	25	1	11.5	12.9	0.2096	0.6471	11.1	12.5	0.2064	0.6496
4	25	2	20.7	18.4	0.4319	0.5111	19.3	19.1	0.0019	0.9656
4	26	1	62.7	76.6	9.4840	0.0021	63.5	76.7	8.6936	0.0032
4	26	2	68.4	75.9	3.8007	0.0512	66.9	74.3	3.5169	0.0607
4	27	1	42.1	47.8	1.4400	0.2301	42.3	47.6	1.2194	0.2695
4	27	2	54.5	62.4	3.4903	0.0617	52.6	61.1	4.0230	0.0449

For peer review only

Table S2. Indicators from mothers in Aurangabad and Gopalganj of infants aged 0-2 months compared to 0-5 months: Estimates, Cochran-Mantel-Haenszel Chi-squares, and probabilities: Bihar 2015 - Continued										
			Unweighted				Weighted			
Domain[a]	Indicator[b]	District[c]	Percent 0-2	Percent 0-5	Chi-square	Probability	Percent 0-2	Percent 0-5	Chi-square	Probability
4	28	1	8.6	8.1	0.0323	0.8575	8.2	7.6	0.0561	0.8127
4	28	2	16.2	13.9	0.5345	0.4647	15.4	13.8	0.2732	0.6012
4	29	1	43.1	48.8	1.4361	0.2308	43.3	48.5	1.1846	0.2764
4	29	2	57.5	64.7	2.9183	0.0876	55.9	63.5	3.2352	0.0721
4	30	1	29.7	35.4	1.6374	0.2007	28.7	34.2	1.5497	0.2132
4	30	2	48.1	53	1.2686	0.2600	46.9	51.4	1.0908	0.2963
4	31	1	30.1	45.9	10.9581	0.0000	29.9	44.9	9.9713	0.0016
4	31	2	47.4	53.0	1.6849	0.1943	44.5	52.7	3.6136	0.0573
4	32	1	5.3	3.8	0.4948	0.4818	5.3	3.6	0.7401	0.3896
4	32	2	9.8	9.0	0.0897	0.7646	9.1	9.9	0.0974	0.7550
4	33	1	32.5	47.8	10.1305	0.0015	32.2	46.7	9.0939	0.0026
4	33	2	50.8	56.4	1.7133	0.1906	48.2	56.2	3.5264	0.0604
4	34	1	6.2	10.0	2.0808	0.1492	6.2	9.9	1.9617	0.1613
4	34	2	13.5	9.0	2.7721	0.0959	14.4	8.9	3.9431	0.0471
4	35	1	45.0	60.3	9.7398	0.0018	44.5	59.3	9.0930	0.0026
4	35	2	64.7	69.5	1.4491	0.2287	62.5	69.0	2.5201	0.1124
5	37	1	73.2	76.6	0.6496	0.4203	72.9	74.9	0.2182	0.6404
5	37	2	80.1	86.1	3.4220	0.0643	78.9	85.5	3.9858	0.0459
5	38	1	55.5	56.5	0.0389	0.8436	54.7	55.3	0.0117	0.9139
5	38	2	52.3	61.3	4.3834	0.0363	51.2	61.6	5.8090	0.0159
5	39	1	5.7	0	3.3034	0.0691	5.4	0	3.6637	0.0556
5	39	2	10.2	8.3	0.2393	0.6247	9.1	6.6	0.3451	0.5569
5	39.5	1	1.4	0	0.0003	0.0833	1.4	0	2.8978	0.0887
5	39.5	2	1.9	1.1	0.5139	0.4735	1.8	0.9	0.7629	0.3824
6	42	1	42.6	47.4	1.0165	0.3134	43.8	47.6	0.6553	0.4182
6	42	2	41.4	46.2	1.3134	0.2518	41.8	46.3	1.1228	0.2893
6	43	1	24.9	29.7	1.3262	0.2495	25.6	30.4	1.3008	0.2541
6	43	2	27.1	29.7	0.4968	0.4809	26.9	31.2	1.2586	0.2619

For peer review only

Table S2. Indicators from mothers in Aurangabad and Gopalganj of infants aged 0-2 months compared to 0-5 months: Estimates, Cochran-Mantel-Haenszel Chi-squares, and probabilities: Bihar 2015 - Continued

			Unweighted				Weighted			
Domain[a]	Indicator[b]	District[c]	Percent 0-2	Percent 0-5	Chi-square	Probability	Percent 0-2	Percent 0-5	Chi-square	Probability
6	44	1	9.8	12.5	1.2833	0.2573	12.2	13.0	0.3958	0.5293
6	44	2	21.1	20.1	0.0586	0.8087	21.6	18.7	0.5178	0.4718
6	45	1	7.5	10.2	0.6321	0.4266	7.8	11.1	0.8502	0.3565
6	45	2	8.2	5.6	0.4009	0.5266	5.2	8.3	0.1670	0.6828
6	46	1	55.5	58.9	0.4904	0.4837	56.6	59.2	0.2960	0.5864
6	46	2	62.8	68.0	1.6764	0.1954	61.7	66.3	1.2736	0.2591
6	47	1	34.0	32.1	0.1768	0.6741	35.9	32.8	0.4369	0.5086
6	47	2	67.3	66.5	0.0344	0.8528	66.6	68.3	0.1745	0.6762
6	48	1	65.6	67.5	0.1782	0.6729	64.9	66.6	0.1312	0.7172
6	48	2	71.1	77.8	3.2208	0.0727	70.7	78.2	3.9664	0.0464
6	49	1	62.7	59.4	0.1537	0.6950	64.5	61.2	0.1691	0.6809
6	49	2	68.1	67.2	0.0166	0.8974	67.3	66.8	0.0011	0.9735
6	50	1	49.0	55.0	1.2527	0.2630	48.4	54.7	1.3188	0.2508
6	50	2	56.8	55.9	0.0964	0.7562	56.1	55.5	0.0615	0.8042
6	51	1	81.1	44.9	14.9736	0.0000	78.0	45.4	11.7395	0.0000
6	51	2	63.3	38.9	4.0901	0.0431	63.7	41.1	3.4654	0.0627
7	52	1	70.3	59.8	5.1727	0.0229	69.2	59.7	4.1723	0.0411
7	52	2	82.3	67.3	16.2014	0.0000	82.1	68.4	13.3711	0.0000

a . 1 Antenatal care, 2 Maternal health, 3 Birth preparedness, 4 FLW Support, 5 Place of birth & attendant, 6 Neonatal Health, 7 Exclusive breastfeeding
b . For text see Table S1
c. 1 Aurangabad, 2 Gopalganj

For peer review only

How well do mothers recall their own and their infant's perinatal events? a two-district study using cross-sectional stratified random sampling in Bihar, India

Journal:	*BMJ Open*
Manuscript ID	bmjopen-2019-031289.R2
Article Type:	Research
Date Submitted by the Author:	13-Oct-2019
Complete List of Authors:	Valadez, Joseph; Liverpool School of Tropical Medicine, International Public Health<br>Devkota, Baburam; Liverpool School of Tropical Medicine, International Public Health<br>Jeffery, Caroline; Liverpool School of Tropical Medicine<br>Hadden, Wilbur; University of Maryland at College Park, Sociology
**Primary Subject Heading**:	Global health
Secondary Subject Heading:	Epidemiology, Global health, Public health, Research methods
Keywords:	Public health < INFECTIOUS DISEASES, maternal recall, Bihar, India, perinatal, surveys, LQAS

How well do mothers recall their own and their infant's perinatal events? a two-district study using cross-sectional stratified random sampling in Bihar, India

Joseph J Valadez[1*], Baburam Devkota[1], Caroline Jeffery[1], Wilbur C. Hadden[2]

[1] Department of International Public Health, Liverpool School of Tropical Medicine, Liverpool, United Kingdom

[2] Department of Sociology, Center for Innovation, University of Maryland, USA

* Corresponding author: Professor Joseph J Valadez, Department of International Public Health, Liverpool School of Tropical Medicine, Pembroke Place, Liverpool, L3 5QA United Kingdom. Email: joseph.valadez@lstmed.ac.uk; Mobile: +44(0)754 8523717

Manuscript word length: 2255

For peer review only

Abstract

Objective: Global monitoring of maternal, newborn and child health (MNCH) programmes use self-reported data subject to recall error which may lead to incorrect decisions for improving health services and wasted resources. To minimize this risk, samples of mothers of infants aged 0-2 and 3-5 months are sometimes used. We test whether a single sample of mothers of infants 0-5 months provides the same information.

Design: An annual MNCH household survey in two districts of Bihar, India (N=6million).

Participants: Independent samples (n=475 each) of mothers of infants 0-5, 0-2 and 3-5 months.

Outcome measures: Main analyses compare responses from the 0-5 and 0-2 month samples with Mantel-Haenszel-Cochran statistics using 51 indicators.

Results: No measurable differences detected in 79.4% of comparisons; 20.6% display differences for the main comparison. Sub-analyses produced similar results. A difference detected for exclusive breastfeeding is due to early commencement of complementary-feeding by older infants. Measurable differences in a third of the indicators on front line worker (FLW) support, about a quarter of indicators of birth preparedness and place of

For peer review only

birth and attendant, a sixth or less of indicators on neonatal and antenatal care. **Conclusions:** Differences in FLW visits and compliance with their advice may be due to seasonal effects: mothers of older infants 3-5 months were pregnant during the dry season; mothers of infants 0-2 months were pregnant during the monsoons, making transportation difficult. Useful coverage estimates can be obtained by sampling mothers with infants 0-5 months as with two samples suggesting that mothers of young infants recall their own perinatal events and those of their children. For some indicators (e.g.,exclusive breast feeding) it may be necessary to adjust targets. Excessive stratification wastes resources, does not improve the quality of information, and increases the burden placed on data collectors and communities, which can increase non-sampling error.

STRENGTHS AND LIMITATIONS OF THIS STUDY

- Strength: The data were produced using stratified random sampling with no apparent design effect leading to an efficient use of information.
- Strength: Data were collected from female participants by female data collectors which is likely to have reduced non-sampling error.
- Strength: The study population is very large covering a large geographical area, reducing the likelihood that the results are pertinent only to a small group of mothers with infants; results may be generalizable.

For peer review only - http://bmjopen.bmj.com/site/about/guidelines.xhtml

- Strength: Both weighted and unweighted results are presented giving strength to the conclusions.
- Limitation: The study primarily assesses data in the 0-2 month sample vis a vis a 0-5 month sample of infants; a comparison between the 3-5 month sample vis a vis the 0-5 month sample would have been informative; however, insufficient overlap of variables in the 0-5 month sample and 3-5 month sample renders this sub-analysis as less informative. Our study compares a 0-2 month subsample with a 3-5 month subsample as an alternative.

Key Words:

Maternal recall, surveys, maternal health, MNCH, LQAS, Bihar, India

INTRODUCTION

The progress toward United Nations' Sustainable Development Goal (SDG) 3 is measured with 9 targets, including the Maternal Mortality Ratio (MMR) and under 5 mortality rate (U5MR)(1, 2). In India, Bihar is one of the largest (population 110 million) and poorest (53% of households are in the lowest wealth index quintile of India(3)) states with high child and maternal mortality (U5MR=54, MMR=208)(4) (see(5) for an evaluation of the health care system in Bihar), and is a priority for donor support for health systems strengthening.

For peer review only - http://bmjopen.bmj.com/site/about/guidelines.xhtml

To accelerate progress toward achieving SDG 3, state governments in India pursue programs of community-based care (see(6, 7) for descriptions and assessments of this approach). Since 2011, the Bihar Ministry of Health has supported an Integrated Family Health Initiative to improve the availability, quality and use of prenatal, perinatal and postnatal care for mothers and infants(8).

The usual way to monitor progress toward achieving these goals is with household surveys. Perhaps the most commonly used surveys are cluster sample surveys such as the Demographic and Health Surveys and the Multiple Indicator Cluster Surveys(9, 10). An alternative design is Lot Quality Assurance Sampling (LQAS), which provides comparable data but is decentralized to local health services organisations and more useful for management and program planning(11). Several states in India find it benefits their programs(12). Surveys rely on the reports of mothers of infants and young children, but these reports are subject to several sources of potential error and bias through interviewees not knowing, forgetting, and having memory errors(13, 14). Studies have shown both that mothers can accurately report significant facts about the birth and care of their children many years after the event(15), but also that even immediately after giving birth mothers may

For peer review only

misreport details(16-18). Studies of maternal recall of her child's vaccination status concluded that due to offsetting errors of maternal reports, the resulting data accurately measured vaccination rates(19); the pattern of error revealed that mothers whose children are up-to-date or nearly so tended to underestimate their child's vaccination status whilst mothers whose children have few vaccinations, overestimate their coverage.

To improve the validity of collected data, knowledge, practice and coverage surveys have used samples of mothers of infants 0-11 months of age or 0-5 months of age and children 6-11 months of age. In Bihar, local organisations departed from this convention of sampling among these three cohorts of children under one year of age and have been monitoring their programs' progress by sampling five dedicated cohorts: mothers of children 0-2, 3-5, 6-8, 9-11 and 12-23 months old with indicators focused on antenatal care, safe delivery practices, infant and young child feeding practices, immunisation, treatment seeking, and more. To avoid the possibility of maternal recall error, each of the five cohorts was asked questions particularly relevant to a child's specific age group.

In countries such as India with high maternal and child mortality rates, regular monitoring of related health

For peer review only - http://bmjopen.bmj.com/site/about/guidelines.xhtml

service coverage is critical to reducing these rates. However, the survey designs should be affordable and sustainable for local health systems; they should also produce precise, unbiased estimates(20). In this study, we explore whether information is gained by sampling cohorts of children aged 0-2 and 3-5 months or whether the sample size can be reduced by 50% by creating one sample cohort aged 0-5 months.

The research question we address is: "Do the health service delivery coverage estimates from a sample of mothers of infants aged 0-5 months differ from those obtained from a sample of mothers of infants ages 0-2 months?" A corollary to this question is: "Do mothers of infants 3-5 months of age display recall error relative to mothers of infants 0-2 months of age for antenatal, delivery or young infant health practices?" We compare district coverage estimates obtained from two independent samples of infants 0-2 months and 0-5 months of age. The implications of this study are important for health systems researchers needing results to appraise and improve their programmes.

METHODS

To answer this question, we collected information from a sample of mothers with infants 0-5 months old and a sample

For peer review only

of mothers with 0-2 month old infants in two districts. This study took place within the context of a larger survey that also sampled children 3-5, 6-8, 9-11 and 12-23 months of age. These four latter samples used questionnaires with variables that either did not overlap at all with questionnaire used to interview the 0-5 month and 0-2 month samples. Due to this constraint, in this study, we only use the two aforementioned groups to assess the measurement of the indicators and refer only to them for the remainder of this paper. The household sampling design we used is a stratified random sample(21). Within each district, the strata are administrative units of the health system, which in Bihar is called a *block*. Within each block the primary sampling unit is the Anganwadi Centre (Community Health Subcentre) Catchment Area (ACCA); 19 ACCA are selected from each block with probability proportional to size. From each ACCA one respondent is randomly selected from each age-group under study using segmentation sampling(22, 23). The sample size 19 for each block is chosen to maximize the probability of correctly classifying a block with reference to performance targets on health related indicators (95% reliability) while balancing the probability (10% margin of error) of incorrectly classifying a block and thereby failing to recognize either the accomplishments of local health care delivery systems or the local population's health care needs(22). For this purpose, principles of Lot Quality Assurance Sampling were used along with established probability tables (24-26).

For peer review only - http://bmjopen.bmj.com/site/about/guidelines.xhtml

There are 14 and 11 blocks in Gopalganj and Aurangabad (N=6 million), the two districts selected for this study, respectively. The total sample sizes are: (a) Gopalganj: 19 x 14 blocks= 266 infants 0-2 months and 266 infants 0-5 months, and (b) Aurangabad: 19 x 11 blocks= 209 infants 0-2 months and 209 infants 0-5 months. The 0-5 month old sample is 60% 0-2 months old and 40% 3-5 months old.

Using summary data from each of the two samples we analyse the data with Cochran-Mantel-Haenszel (CMH)(27) tests for 51 dichotomous indicators (See Supplementary File Table S1) common to the two samples. The CMH tests theoretically have a Chi-square probability distribution with 1 degree of freedom. With a sufficient number of respondents or a sufficient number of blocks, the CMH test is equivalent to conditional logistic regression(28: 114-115). In this analysis both the number of respondents and the number of blocks only approach sufficiency. Consequently, the calculated chi-squares and probabilities must be considered as approximations of their true values.

We calculate both unweighted and weighted estimates. The unweighted estimates permit the results from smaller blocks to have equal weight vis à vis larger ones. Since the research question concerns an analysis of which age cohort is most informative, the weighted estimates may not be as

For peer review only - http://bmjopen.bmj.com/site/about/guidelines.xhtml

useful as the unweighted ones. However, the weighted estimates provide better point estimates of the indicators at the district level. The effect of the weights on the Chi-square statistics is to increase the contribution of the larger blocks and decrease the contribution of the smaller blocks. Hence, we report both sets of results (See Supplementary File Tables S2-S3).

The Chi-square probability distribution puts the differences between the districts on a probability scale (See Supplementary File Table S2). To determine meaningful differences in responses between the two age cohorts we used a probability of 0.05 as a cut-off value and consider differences with probabilities less than 0.05 to be possibly meaningful and those with larger probabilities to be likely due to sampling errors. With 102 comparisons (51 indicators weighted or unweighted) we must expect some to exceed this cut-off by chance alone. If all of the comparisons were independent, we might randomly find 5 differences, but many of the indicators measure related events (e.g., number of ANC visits and tetanus toxoid vaccinations) and the weighted and unweighted estimates are similar, so these indicators are not all independent and it is not possible to calculate an expected number of

For peer review only - http://bmjopen.bmj.com/site/about/guidelines.xhtml

differences nor is it appropriate to interpret these probabilities as measures of “statistical significance”.

Patient and Public Involvement

This study does not involve patients. Also, the public was not involved in the design, conduct and reporting of the research. The public was engaged as interviewees. To ensure local engagement we coordinated with the Bihar Ministry of Health, local implementing non-governmental organizations and our donor. We also shared the results with them and offered further dissemination of results.

RESULTS

We find a high level of agreement between the two samples (Table 1). Out of 102 weighted and unweighted comparisons between the estimates from the 0-2 month and 0-5 month samples there is no probable difference in 81 (79.4%) in both the unweighted and weighted estimates. We detect that probable differences exist for 13 comparisons (12.7%). For the remaining eight comparisons the weighted and unweighted estimates disagree. The weighted estimates find seven differences that the unweighted estimates do not; the unweighted estimates find one difference that the weighted estimates do not find.

For peer review only - http://bmjopen.bmj.com/site/about/guidelines.xhtml

Table 1. Number of indicators by probability of a difference between the 0-2 months and the 0-5 months samples for weighted and unweighted samples

Unweighted	Weighted					
	Aurangabad		Gopalganj		Total	
	>= .05	< .05	>= .05	< .05	>= .05	< .05
>= .05	40	3	41	4	81	7
< .05	0	8	1	5	1	13

For different health service domains, the number of indicator comparisons varies from two (Exclusive Breastfeeding-EBF) to 24 concerning home visits by Front Line Worker (FLW) support (Table 2). The two principal FLW are Anganwadi workers and Accredited Social Health Activists (ASHA).

For peer review only

Table 2. Number of indicator comparisons by subject domain showing a measurable difference using weighted and unweighted estimates of 0-2 months and 0-5 months samples

Health Service Domain	Total comparisons	No measurable difference between 0-2 and 0-5 months results	Measurable difference between 0-2 and 0-5 months results			
			Both	Unweighted only	Weighted only	Percent (%) indicators with different results
Antenatal care	22	21	0	0	1	5
Place of birth & attendant	8	6	1	0	1	25
Birth preparedness	18	13	3	0	2	28
Front Line Worker support	24	16	6	0	2	33
Maternal health	8	8	0	0	0	0
Neonatal care	20	17	1	1	1	15
Exclusive Breastfeeding	2	0	2	0	0	100
Totals	102	81	13	1	7	21.9

For peer review only - http://bmjopen.bmj.com/site/about/guidelines.xhtml

In the FLW support domain 33% of comparisons have probable differences. The neonatal health domain has 20 comparisons and the birth preparedness domain has 18; in these domains 15% and 28% show probable differences, respectively. Place of birth and attendant and maternal health each have eight comparisons with 25%, or two comparisons, and 0 comparisons, respectively, showing a possible difference. The differences between the two samples cluster around home visits from FLW and behaviours associated with birth preparedness and neonatal care. Details of these differences are listed in Table 3.

For two indicators, both the weighted and unweighted estimates display probable differences between the 0-2 and 0-5 months samples in both districts. For indicator #52, the proportion exclusively breastfeeding, the 0-5 months cohort has the lower estimate, and indicator #24, the proportion of mothers visited by an ASHA at least once during their last pregnancy, the 0-5 months sample gives the higher estimate, about 74%, compared to 63% in the 0-2 months sample (See Supplementary Tables S2-3).

Additional analyses comparing subsamples of mothers of infants 0-2 months and 3-5 months from the 0-5 months

For peer review only

sample, the sample of mothers of infants 0-2 months and the subsample of infants 0-2 month, and the sample of mothers of infants 3-5 months and the subsample of infants 3-5 months produced similar results (See Supplementary Text and Tables S4a-b, Tables S5a-b and Table S6).

For peer review only

Table 3. Indicators by health service domain showing measurement differences between 0-2 months and 0-5 months samples

Health Service Domain and Indicator	Indicator No.	District	Weighted Coverage (%)		p-value		Estimate type
			0-2 months	0-5 months	Unweighted Estimate	Weighted Estimate	
**Antenatal care**							
Proportion of mothers of infants (0-2/0-5 months) registered during their last pregnancy	1	Aurangabad	85.2	77.6	0.0552	0.0365	Weighted
**Place of birth & attendant**							
Proportion of mothers of infants (0-2/0-5 months) whose last child was delivered at a public facility	38	Gopalganj	51.2	61.6	0.0363	0.0159	Both
Proportion of mothers of infants (0-2/0-5 months) whose last child was delivered at a health facility (private or public facility)	37	Gopalganj	78.9	85.5	0.0643	0.0459	Weighted
**Birth preparedness**							
Proportion of mothers of infants (0-2/0-5 months) who planned transportation to health facility in their last pregnancy (home & institutional delivery)	15	Gopalganj	45.7	56.0	0.0266	0.0158	Both
Proportion of mothers of infants (0-2/0-5 months) who identified persons to care for the baby immediately after birth (home + institutional delivery)	17	Gopalganj	51.8	62.6	0.0255	0.0103	Both
Proportion of mothers of infants (0-2/0-5 months) who planned for institutional delivery and	23	Aurangabad	62.5	47.0	0.0039	0.0052	Both

For peer review only - http://bmjopen.bmj.com/site/about/guidelines.xhtml

For peer review only

identified person to accompany her during the delivery							
Proportion of mothers who planned for institutional delivery of infants (0-2/0-5 months) who had a new blade & thread for their delivery	19	Aurangabad	23.5	14.5	0.062	0.0429	Weighted
Proportion of mothers who planned institutional delivery of infants (0-2/0-5 months) who arranged clean cloth for mothers and baby	21	Aurangabad	43.6	31.2	0.0546	0.0137	Weighted
**Front Line Worker support**							
Proportion of mothers of infants (0-2/0-5 months) who were visited by ASHA at least once during their last pregnancy	24	Aurangabad	62.2	75.2	0.0023	0.0042	Both
		Gopalganj	63.5	73.0	0.0284	0.0175	Both
Proportion of mothers of infants (0-2/0-5 months) visited at home by FLWs at least once during their last pregnancy	26	Aurangabad	63.5	76.7	0.0021	0.0032	Both
Proportion of mothers of infants (0-2/0-5 months) visited at home by ASHA within 24 hours of last delivery	31	Aurangabad	29.9	44.9	0.0009	0.0016	Both
Proportion of mothers of infants (0-2/0-5 months) visited at home by any FLW within 24 hours of last delivery	33	Aurangabad	32.2	46.7	0.0015	0.0026	Both
Proportion of mothers of infants (0-2/0-5 months) visited at home by any FLW within first week of last delivery	35	Aurangabad	44.5	59.3	0.0018	0.0026	Both
Proportion of mothers of infants (0-2/0-5 months) visited at home by any AWW within the first week of the last delivery	34	Gopalganj	14.4	8.9	0.0959	0.0471	Weighted
Proportion of mothers of infants (0-2/0-5 months) visited by	27	Gopalganj	52.6	61.1	0.0617	0.0449	Weighted

For peer review only - http://bmjopen.bmj.com/site/about/guidelines.xhtml

ASHAs at least once during their last trimester of pregnancy							
**Infant care**							
Proportion of infants aged (0-2/0-5 months) who were delivered at home continued with dry cord care	51	Aurangabad	78.0	45.4	0.0001	0.0006	Both
		Gopalganj	63.7	41.1	0.0431	0.0627	Unweighted
Proportion of infants aged (0-2/0-5 months) weighed after birth (Public facility/Private facility/Home)	48	Gopalganj	70.7	78.2	0.0727	0.0464	Weighted
**Exclusive Breastfeeding**							
Proportion of infants (0-2/0-5 months) breast-fed in the past 24 hours (Exclusively Breast-fed)	52	Aurangabad	69.2	59.7	0.0229	0.0411	Both
		Gopalganj	82.1	68.4	0.0001	0.0003	Both

For peer review only - http://bmjopen.bmj.com/site/about/guidelines.xhtml

DISCUSSION

Statement of principal findings

There are no measurable differences in coverage estimates for 79.4% (81 comparisons) of the indicator comparisons between the samples of mothers with infants 0-2 months old versus mothers of infants 0-5 months old; 12.7% (13 comparisons) display measurable differences. The remaining 7.8% (eight comparisons) display discrepancies between the weighted and unweighted estimates.

Strengths and weaknesses of the study

The strengths of this study are that it compares estimates from two independent samples and that there are many estimates from diverse domains. The weaknesses of this study are that the data were collected in only 2 districts of 1 state in India and in different months of a single year, and that indicators from the sample of mothers of 3-5 month old infants comparable to those of the 0-2 month old infants, using the same questionnaire, were not collected. Supplemental analyses comparing 0-2 and 3-5 month subsamples of the 0-5 sample did not uncover evidence of bias due to the combination of these two age groups.

For peer review only - http://bmjopen.bmj.com/site/about/guidelines.xhtml

Strengths and weaknesses in relation to other studies

Other studies of maternal recall bias have sought a "gold standard" to represent reality and to evaluate measures. Our study, of course, is interested in reality, but this study compares alternative measures needed to assess the Bihar health program. It also uses a complete sample of the age grouping under study rather than just a sub-sample of a larger age grouping. A weakness of this approach is that the analysis does not result in a formal statistical test; our conclusion is based on the weight of the evidence.

Meaning of the study

The evidence indicates that samples of the broader group yield comparable results to those of the narrower age group. It is not necessary to double the total sample by measuring independently 0-2 months and 3-5 months cohorts of children. These results also tend to dispel the hypothesis that maternal recall is problematic for mothers during the first 6 months following delivery. Our results are more consistent with conclusions presented in earlier research(15) and they support those organizations collecting data with 0-5 month cohorts.

For peer review only - http://bmjopen.bmj.com/site/about/guidelines.xhtml

Indicator #52, EBF, displayed two comparisons measuring decreases in both districts. This is not surprising as fewer infants are expected to be exclusively breast fed in a sample ranging from 0 to 5 months than a sample ranging 0 to 2 months; mothers introduce complementary feeding and liquids as infants age despite this being a health risk. This difference could be accommodated by adjusting expectations and targets for the indicator.

Unanswered questions and future research

Further investigation and consideration of the differences is warranted. The eight differences found in the FLW support indicators deserve more scrutiny. Seven show higher estimates for the 0-5 cohort and one has a higher estimate for the 0-2 month cohort. The former seven differences may be due to excessive rainfall during the July-September (monthly average 318.95mm, range:195.99-395.8mm) versus the lesser rainfall during June-April (monthly average 25.52mm, range: 0.3-27.88mm) which in the last trimester may have reduced the access of ASHA in the 0-2 month cohort (29). Indicators such as these may be particularly sensitive to rainfall and may explain why more mothers in the 0-5 month cohort displayed higher FLW visitation estimates since FLW

For peer review only

were not impeded by the monsoon and the resulting muddy roads.

Differences in birth preparedness and institutional birth may be a consequence of the differences in rainfall or in FLW support with increases a response to the need for more careful planning when transportation is difficult and decreases due to a reduction in the educational efforts of the FLW. Or, some of these differences may just be due to noise in the data.

CONCLUSIONS

Overall, the answer to the research question, "Can one get the same district coverage estimates from a sample of mothers of infants aged 0-5 months as from a sample of mothers of infants ages 0-2 months?" is yes. This result can be paraphrased as: mothers do not display increased recall errors of their perinatal health care behaviour in a cohort of mothers with infants 0-5 months as compared with mothers with younger infants. Substantial resources and effort can be saved using a survey design that avoids needless expenses to collect data that provides insubstantial amounts of information. It also reduces the burden on data collectors and community participants.

For peer review only - http://bmjopen.bmj.com/site/about/guidelines.xhtml

Fatigue to both groups can result in needless non-sampling error.

For peer review only

ACKNOWLEDGMENTS

We gratefully acknowledge the essential roles of Hemant Das, Alok Prahdan and Sanjay Biswa for their careful field work supporting the LQAS survey and the HMIS data retrieval. We thank Prof Imelda Bates, Prof Brian Faragher and Nancy Vollmer for their valuable feedback on an earlier version of this manuscript. This work was supported by the Bill and Melinda Gates Foundation (Investment ID OPP1142889).

ETHICS

The Ethical Committees of the authors' institutes approved the protocol, study instruments and consent procedures for the survey data collection.

COMPETING INTERESTS

The authors have read the statement on competing interests and declare that they have none.

AUTHORS' CONTRIBUTIONS

JJV, BD developed the research question and survey design; WH, CJ carried out the statistical analyses; JJV obtained the funding and donor support for the research; BD trained and managed the survey teams in Bihar; JJV, WH, CJ interpreted the data; CJ responsible for data curation; all authors wrote and reviewed the paper.

COMPETING INTERESTS

The authors have no competing interests.

FUNDING

This research was funded by the Bill and Melinda Gates Foundation Investment ID OPP1142889

For peer review only - http://bmjopen.bmj.com/site/about/guidelines.xhtml

DATA SHARING STATEMENT

Data are available upon reasonable request.

REFERENCES

1. UNSTATS. Official list of MDG indicators: United Nations Statistics Division, Department of Economic and Social Affairs; 2005 [Available from: https://unstats.un.org/unsd/mdg/Host.aspx?Content=Indicators/OfficialList.htm.

2. UN DESA. Sustainable Development Goals: Division for Sustainable Development Goals, United Nations Department of Economic and Social Affairs; 2015 [Available from: https://www.un.org/sustainabledevelopment/sustainable-development-goals/.

3. International Institute for Population Sciences (IIPS) and ICF. National Family Health Survey (NFHS-4), 2015-16: India. Mumbai: IIPS; 2017.

4. MOSPI. Millennium Development Goals India Country Report 2015. New Delhi: Social Statistics Division, Central Statistics Office, Ministry of Statistics and Programme Implementation; 2015.

5. Karvande S, Sonawane D, Chavan S, Mistry N. What does quality of care mean for maternal health providers from two vulnerable states of India? Case study of Bihar and Jharkhand. Journal of Health, Population and Nutrition. 2016;35(6):1-10.

6. Mohan P, Kishore B, Singh S, Bahl R, Puri A, Kumar R. Assessment of implementation of integrated management of neonatal and childhood illness in India. Journal of Health, Population and Nutrition. 2011;29(6):639-38.

7. Neogi SB, Sharma J, Chauhan M, Khanna R, Chokshi M, Srivastava, et al. Care of newborn in the community and at home. Journal of Perinatology. 2016;36(Suppl 3):s13-7.

8. CARE Family Health. Integrated Family Health Initiative: Catalysing change for healthy communities. 2013.

9. Blanc AK, Warren CE, McCarthy KJ, Kimani J, Ndwiga C, RamaRao S. Assessing the validity of indicators of the quality of maternal and newborn health care in Kenya. Journal of Global Health. 2016;6(1):1-11.

10. Eisele TP, Rhoda DA, Cutts FT, Keating J, Ren R, Barros AJD, et al. Measuring coverage in MNCH: Total survey error and the interpretation of intervention coverage estimates from household surveys. PLoS Medicine. 2013;10(5):e1001386.

11. Anoke SC, Mwai P, Jeffery C, Valadez JJ, Pagano M. Comparing two survey methods of measuring health-related indicators: Lot Quality Assurance Sampling and Demographic Health Surveys. Tropical Medicine and International Health. 2015;20(12):1756-70.

12. Valadez JJ, Devkota B, Pradhan MM, Meherda P, Sonal GS, Dhariwal A, et al. Improving malaria treatment and prevention in India by aiding district managers to manage their programmes with local information: a trial assessing the impact of Lot Quality Assurance Sampling on programme outcomes. Trop Med Int Health. 2014.

13. Tourangeau R. Remembering what happened: Memory errors and survey reports. In: Stone AA, Jobe JB, Bachrach CA, Cain VS, Kurtzman HS, editors. The Science of

For peer review only

Self-report : Implications for research and practice. Mahwah, NJ: Lawrence Erlbaum; 2000.
14. Bradburn NM. Temporal representation and event dating. In: Stone AA, Jobe JB, Bachrach CA, Cain VS, Kurtzman HS, editors. The Science of Self-report : Implications for research and practice. Mahwah, NJ: Lawrence Erlbaum; 2000.
15. Tomeo CA, Rich-Edwards JW, Michels KB, Berkey CS, Hunter DJ, Frazier AL, et al. Reproducibility and validity of maternal recall of pregnancy-related events. Epidemiology. 1999;10(6):774-7.
16. Elkadry E, Kenton K, White P, Creech S, Brubaker L. Do mothers remember key events during labor? American Journal of Obstetrics and Gynecology. 2003;189(1):195-200.
17. Li R, Scanlon KS, Serdula MK. The validity and reliability of maternal recall of breastfeeding practice. Nutrition Reviews. 2005;63(4):103-10.
18. Miles M, Ryman TK, Dietz V, Zell E, Luman ET. Validity of vaccination cards and parental recall to estimate vaccination coverage: A systematic review of the literature. Vaccine. 2013;31:1560-8.
19. Valadez JJ, Weld LH. Maternal recall error of child vaccination status in a developing nation. Am J Public Health. 1992;82(1):120-2.
20. Chan M, Kazatchkine M, Lob-Levyt J, Obaid T, Schweizer J, Sidibe M, et al. Meeting the demand for results and accountability: a call for action on health data from eight global health agencies. PLoS Med. 2010;7(1):e1000223.
21. Valadez JJ, Weiss W, Leburg C, Davis R. Assessing Community Health Programs: A Trainer's Guide. Using LQAS for Baseline Surveys and Regular Monitoring. 2nd ed. St Albans: Teaching-aids At Low Cost; 2007.
22. Turner AG, Magnani RJ, Shuaib M. A not quite as quick but much cleaner alternative to the Expanded Programme on Immunization (EPI) Cluster Survey design. International Journal of Epidemiology. 1996;25(4):198-203.
23. Davis RH, Valadez JJ. Improving the collection of knowledge, attitude and practice data with community surveys: a comparison of two second-stage sampling methods. Health Policy Planning. 2014;29(8):1054-60.
24. Dodge HF, Romig HG. Sampling inspection tables: single and double sampling. 2nd ed. New York: John Wiley & Sons; 1959.
25. Robertson SE, Joseph J V. Global review of health care surveys using lot quality assurance sampling (LQAS), 1984–2004. Social Science & Medicine. 2006;63:1648-60.
26. Valadez JJ. Assessing child survival programmes in developing countries: testing lot quality assurance sampling. Boston: Harvard University Press; 1991.
27. Mantel N. Chi-square tests with one degree of freedom: extensions of the Mantel-Haenszel procedure. J Am Stat Assoc. 1963;58(303):690-700.
28. Agresti A. An Introduction to Categorical Data Analysis. 2nd ed. Hoboken, New Jersey: Wiley-Interscience; John Wiley & Sons; 2007.
29. World Weather Online. Patna Monthly Climate Averages, Bihar, India 2018 [Available from: https://www.worldweatheronline.com/patna-weather-averages/bihar/in.aspx.

For peer review only

Supplementary Text for Tables S4a through S6

We make four comparisons to inform an evaluation of these data. The first compares the 0-5 month sample to the 0-2 month sample and is presented in Table 2 in the main text and Tables S2-3. In a second comparison, indicators estimated for the 0-2 month old **subsample** are compared to the 3-5 month old **subsample** to assess the internal consistency of estimates for the entire 0-5 sample (Table 4a-b). In the third comparison, the 0-2 **subsample** of the 0-5 sample is compared to the 0-2 month **sample** to assess the sampling variability for this age group (Tables S5a-b). Finally, in the fourth comparison, the 3-5 **subsample** of the 0-5 sample is compared to the 3-5 month **sample** (Table S6). This last comparison is limited because the 3-5 month sample collected limited data; there are only 3 indicators common to the two samples.

Table S4a has the complete results for the second comparison with point estimates for the two **subsamples** 0-2 and 3-5 of the 0-5 sample, differences and estimated confidence intervals for the estimates and differences of 104 comparisons. Fourteen indicators for which the estimated confidence interval of the difference does not include zero are listed in Table S4b. The table has 2 panels. In the top panel are 6 indicators for which a difference is also reported in Table S2. Table S2 compares the 0-2 month sample and the 0-5 month sample. For each of these indicators the reported difference is in the same direction. Two of these indicators are indicator 52, exclusive infant breast feeding, in the 2 provinces. These differences are not surprising as it is common for mothers to introduce supplemental foods as infants age. The authors speculate in their paper that the timing of the monsoon may have reduced some of the indicators for the younger infants. Of the remaining four indicators of the top panel, three of the indicator differences are negative, lower for the younger infants and might plausibly be related to a monsoon. In the bottom panel of Table S4b are 8 indicators where zero is not in the confidence interval of the difference between subsamples, which suggests difference, but the results between two full-samples in Tables S2 and S3 suggest there is no meaningful difference, except that one of them, indicator 37 in Gopalganj; it does show a difference in Table S2. Of these 8, the difference between samples is in the same direction five times and in a different direction three times. For the 5 indicators where the differences are in the same direction, the absolute value of the differences between 0-2 subsample and the 3-5 subsample are larger than the absolute values of the differences between the 0-2 month sample and the 3-5 subsample. For these 5 indicators the 0-2 month **sample** is more like the 3-5 **subsample** than the 0-2 **subsample**. For the 3 indicators where the differences are in the different directions the 0-2 **subsample** closely resembles the 0-2 month **sample**; two of the estimates are different by less than 1 percent and the third by 2.4 percent.

A third comparison is in Tables S5a-b. In Table S5a the 0-2 **subsample** is compared to the 0-2 month **sample**. The expectation here would be that there are no differences because these two samples are designed to represent the same population. However, this expectation is not met; there are 10 differences where the confidence interval for the difference does not contain 0. These differences are listed in Table S5b. Nine of these 10 indicators are also among the differences in Table S3 and Table 3 in the paper. For 3 of these 9 differences the 3-5 subsample is closer to the 0-2 month sample than it is the 0-2 subsample (indicators 15, 17-2, 51); and for 3 indicators the 3-5 subsample is further from the 0-2 month sample than the 0-2 subsample (indicators 31, 33, 35). For 3 indicators the 0-2 and 3-5 subsample indicator values are nearly equal (indicators 23, 24, 26). For the final indicator in this list (indicator 17-1, the indicator for which zero is in the confidence interval of the difference between the 0-2

For peer review only

month sample and the 0-5 sample in Table S3) the difference between the indicator values for the 0-2 and 3-5 subsamples and the 0-2 month sample are about equal in magnitude but have opposite signs.

Results from the fourth comparison, comparing the 3-5 **subsample** of the 0-5 **sample** to the 3-5 month sample are limited, as noted above. There are only 3 indicators in the 2 districts – six comparisons. Zero is within the confidence interval of 5 of the differences (Table S6).

We note in the paper that in making this number of comparisons one must expect that some will be large enough to be considered meaningful by chance alone. In the above analysis there are 3 comparisons of 51 indicators in 2 provinces producing 16, 14 and 10 differences and a fourth comparison with 1 difference in 6. In their paper the authors find that some of these differences are readily understood and others may be interpreted effects of monsoon rains. Post hoc interpretation is risky here; many of these differences may be noise in the data. There is evidence in the third comparison to support this position with 3 differences moving the 0-5 **sample** closer to the 0-2 month **sample**, 3 moving it away, and 4 not moving it one way or the other. Furthermore, 9 of the 10 differences in the third comparison are also differences in the first comparison, suggesting that about half the differences between the 0-2 month sample and the 0-5 sample may be due to differences between the 0-2 month **sample** and the 0-2 **subsample** of the 0-5 sample.

The second comparison, comparing the 0-2 subsample to the 3-5 subsample, provides evidence both undermining and supporting the conclusion that a 0-5 sample will provide the same answers as two samples 0-2 and 3-5. On the one hand 5 of the fourteen differences in the third comparison are also in the first, the comparison of the 0-5 sample to the 0-2 sample, suggesting that the inclusion of 3-5 month olds in the 0-5 sample might contribute to the differences. On the other hand, in 5 out of the 8 indicators that are not different between the 0-2 and 0-5 samples, the 3-5 subsample more closely matches the 0-2 month sample than does the 0-2 subsample of the 0-5 sample. That this is true in general and not only for these extreme differences is suggested by the mean absolute differences between samples which are 5.2 for the differences between the 0-2 month **sample** and 3-5 month **subsample** and 6.6 for the differences between the 0-2 month **subsample** and the 3-5 month **subsample**.

Finally, the overall result of all these comparisons is the same as that of the comparison presented in the main text of the paper: in each comparison 85% or more of the indicators show no differences between the samples. The most consistent evidence of differencCae is between the 0-2 **subsample** of the 0-5 sample and the 0-2 month **sample**.

For peer review only

Table S1. Subject domain and indicators labels

Domain[a]	Indicator	Indicator text
1	1	Proportion of mothers of infants (0-2/0-5) months who were registered during their last pregnancy
1	2	Proportion of mothers of infants (0-2/0-5) months whose last pregnancy was registered in the first three months of pregnancy
1	3	Proportion of mothers of infants (0-2/0-5) months attended at least one ANC visit during their last pregnancy
1	4	Proportion of mothers of infants (0-2/0-5) months attended 3 or more ANC visits during their last pregnancy
1	5	Proportion of mothers of infants (0-2/0-5) months attended 4 or more ANC visits during their last pregnancy
1	6	Proportion of mothers of infants (0-2/0-5) months who attended at least one ANC visit where BP was checked during her last pregnancy
1	7	Proportion of mothers of infants (0-2/0-5) months who attended at least one ANC where at least one sonography was performed during her last pregnancy
1	8	Proportion of mothers of infants (0-2/0-5) months who attended at least one ANC where at least one abdominal examination was performed during her last pregnancy
1	9	Proportion of mothers of infants (0-2/0-5) months who attended at least one ANC where at least one urine test was performed during her last pregnancy
1	10	Proportion of mothers of infants (0-2/0-5) months who attended at least one ANC where at least one blood test was performed during her last pregnancy
1	40	Proportion of mothers of infants (0-2/0-5) months who have used an ambulance to reach an institution for last delivery
2	11	Proportion of mothers of infants (0-2/0-5) months who were protected against tetanus in their last pregnancy (Neonatal TT)
2	12	Proportion of mothers of infants (0-2/0-5) months who received two or more doses of TT injection in their last pregnancy
2	13	Proportion of mothers of infants (0-2/0-5) months who received IFA for 100 days or more during their pregnancy
2	14	Proportion of mothers of infants (0-2/0-5) months consumed IFA for 100 days or more in their pregnancy
3	15	Proportion of mothers (home + institutional delivery) of infants (0-2/0-5) months who planned transportation to health facility in their last pregnancy
3	16	Proportion of mothers (home + institutional delivery) of infants (0-2/0-5) months who have identified anybody who would donate blood in the case of emergency in their last pregnancy

For peer review only

3	17	Proportion of mothers (home + institutional delivery) of infants (0-2/0-5) months who have identified persons who would take care of the baby immediately after birth
3	18	Proportion of mothers (home + institutional delivery) of infants (0-2/0-5) months who have arranged new blade & thread for their last delivery
3	19	Proportion of mothers who planned for institutional delivery of infants (0-2/0-5) months who arranged new blade & thread for their delivery
3	20	Proportion of mothers (home + institutional delivery) of infants (0-2/0-5) months who have arranged clean cloth for mothers and baby
3	21	Proportion of mothers who planned institutional delivery of infants (0-2/0-5) months who arranged clean cloth for mothers and baby
3	22	Proportion of mothers of infants (0-2/0-5) months who have saved money for the delivery
3	23	Proportion of mothers who planned for institutional delivery of infants (0-2/0-5) months identified person to accompany her during the delivery
4	24	Proportion of mothers of infants (0-2/0-5) months who were visited by ASHA at least once during their last pregnancy
4	25	Proportion of mothers of infants (0-2/0-5) months who were visited by AWW at least once during their last pregnancy
4	26	Proportion of mothers of infants (0-2/0-5) months who were visited by FLWs at least once during their last pregnancy
4	27	Proportion of mothers of infants (0-2/0-5 months) visited by ASHAs at least once during their last trimester of pregnancy
4	28	Proportion of mothers of infants (0-2/0-5) months who were visited by AWW at least once during their last pregnancy
4	29	Proportion of mothers of infants (0-2/0-5) months who were visited by any FLW in the last trimester during their last pregnancy
4	30	Proportion of mothers of infants (0-2/0-5) months who were visited 2 or more times by any FLW in the last trimester during their last pregnancy
4	31	Proportion of mothers of infants (0-2/0-5) months who were visited home by ASHA within 24 hours of last delivery
4	32	Proportion of mothers of infants (0-2/0-5) months who were visited home by AWW within 24 hours of last delivery
4	33	Proportion of mothers of infants (0-2/0-5) months who were visited home by any FLW within 24 hours of last delivery
4	34	Proportion of mothers of infants (0-2/0-5) months who were visited home by any AWW within the first week of last delivery

For peer review only

4	35	Proportion of mothers of infants (0-2/0-5) months who were visited home by any FLW within first week of last delivery
5	37	Proportion of mothers of infants (0-2/0-5) months whose last child was delivered at a health facility (private or public facility)
5	38	Proportion of mothers of infants (0-2/0-5) months whose last child was delivered at public facility
5	39	Proportion of mothers (home delivery) of infants (0-2/0-5) months who had home delivery attended by skilled birth attendant (SBA)
5	39.5	Proportion of mothers (home + institutional delivery) of infants (0-2/0-5) months who had home delivery attended by skill birth attendant (SBA)
6	42	Proportion of infants (home + institutional delivery) aged (0-2/0-5) months with nothing applied to the umbilical cord after cutting and tying in their last delivery
6	43	Proportion of mothers (home + institutional delivery) of infants (0-2/0-5) months who have delivered baby practiced skin to skin care (STSC) immediately after birth
6	44	Proportion of mothers with institutional delivery of infants (0-2/0-5) months who continued skin to skin care (STSC) at home
6	45	Proportion of mothers with home delivery of infants (0-2/0-5) months who continued skin to skin care (STSC) later
6	46	Proportion of mothers of infants (0-2/0-5) months who were breastfed within one hour of birth
6	47	Proportion of infants aged (0-2/0-5) months who have received delayed bath (between 48 hours and before 7 days after birth) (Public facility/Private facility/Home)
6	48	Proportion of infants aged (0-2/0-5) months who were weighed after birth (Public facility/Private facility/Home)
6	49	Proportion of infants aged (0-2/0-5) months who were delivered at HF received dry cord care
6	50	Proportion of infants aged (0-2/0-5) months who were delivered at HF continued with dry cord care
6	51	Proportion of infants aged (0-2/0-5) months who were delivered at home continued with dry cord care
7	52	Proportion of infants (0-2/0-5) months who were breast-fed in the past 24 hours (Exclusively Breast-fed)

a. 1 Antenatal care, 2 Maternal health, 3 Birth preparedness, 4 Front Line Worker Support, 6 Place of birth & attendant, 7 Exclusive Breastfeeding

For peer review only

Table S2. Indicators from two samples in Aurangabad and Gopalganj of mothers of infants aged 0-2 months compared to 0-5 months: Estimates, Cochran-Mantel-Haenszel Chi-squares, and probabilities: Bihar 2015

			Unweighted				Weighted			
Domain[a]	Indicator[b]	District[c]	Percent 0-2	Percent 0-5	Chi-square	Probability	Percent 0-2	Percent 0-5	Chi-square	Probability
1	1	1	86.6	79.9	3.6775	0.0552	85.2	77.6	4.3758	0.0365
1	1	2	85.3	85.3	0.0000	1.0000	85.0	85.4	0.0187	0.8911
1	2	1	44	40.7	0.4996	0.4797	43.0	38.3	1.0147	0.3138
1	2	2	47.7	47.0	0.0304	0.8616	47.5	48.2	0.0212	0.8843
1	3	1	84.7	80.9	1.2398	0.2655	83.8	80.4	0.9592	0.3274
1	3	2	81.2	78.6	0.5985	0.4391	80.6	77.8	0.6424	0.4229
1	4	1	60.3	63.6	0.4931	0.4826	61.1	63.1	0.1792	0.6720
1	4	2	59.4	63.2	0.8252	0.3637	57.4	61.0	0.7211	0.3958
1	5	1	42.1	45.0	0.3491	0.5546	43.2	45.7	0.2561	0.6128
1	5	2	44.7	47.7	0.4866	0.4854	43.4	46.9	0.6302	0.4273
1	6	1	59.8	59.3	0.0107	0.9177	58.9	60.1	0.0644	0.7996
1	6	2	67.7	63.5	1.0487	0.3058	66.3	63.3	0.5176	0.4719
1	7	1	42.6	39.2	0.5043	0.4776	42.0	39.5	0.2824	0.5952
1	7	2	70.3	73.3	0.6025	0.4376	69.2	72.6	0.7537	0.3853
1	8	1	44.0	45.9	0.1685	0.6815	42.7	44.8	0.2028	0.6525
1	8	2	53.4	54.1	0.0322	0.8576	52.4	54.5	0.2438	0.6215
1	9	1	45.9	42.6	0.5004	0.4793	45.8	41.7	0.7523	0.3857
1	9	2	42.9	43.6	0.0321	0.8579	42.8	43.5	0.0231	0.8792
1	10	1	67.0	59.8	2.6555	0.1032	67.2	59.6	2.9924	0.0837
1	10	2	55.3	58.3	0.5191	0.4712	54.8	58.5	0.7674	0.3810
1	40	1	11.5	17.7	3.4797	0.0621	10.6	16.0	2.8329	0.0924
1	40	2	8.6	10.2	0.3686	0.5438	8.5	9.5	0.1374	0.7109
2	11	1	98.6	98.1	0.1451	0.7033	98.5	98.1	0.0844	0.7715
2	11	2	97.0	96.6	0.0614	0.8044	96.9	97.1	0.0131	0.9089
2	12	1	68.4	66.5	0.1763	0.6746	69.3	67.5	0.1626	0.6868
2	12	2	79.7	79.3	0.0116	0.9141	81.9	80.4	0.2143	0.6434
2	13	1	3.8	2.4	0.7384	0.3902	4.0	2.6	0.6021	0.4378
2	13	2	3.4	5.6	1.5745	0.2096	3.0	5.1	1.6016	0.2057

For peer review only

Table S2. Indicators from mothers two samples in Aurangabad and Gopalganj of mothers of infants aged 0-2 months compared to 0-5 months: Estimates, Cochran-Mantel-Haenszel Chi-squares, and probabilities: Bihar 2015 - Continued										
			Unweighted				Weighted			
Domain[a]	Indicator[b]	District[c]	Percent 0-2	Percent 0-5	Chi-square	Probability	Percent 0-2	Percent 0-5	Chi-square	Probability
2	14	1	1.4	1.9	0.1474	0.7010	1.6	2.0	0.1025	0.7489
2	14	2	1.9	3.8	1.7161	0.1902	1.7	3.6	1.8535	0.1734
3	15	1	26.3	34.0	3.1510	0.0759	25.9	33.3	2.9594	0.0854
3	15	2	47.0	56.4	4.9192	0.0266	45.7	56.0	5.8262	0.0158
3	16	1	4.3	3.8	0.0639	0.8004	4.3	3.6	0.1410	0.7073
3	16	2	10.2	13.9	1.8263	0.1766	9.7	14.3	2.8157	0.0933
3	17	1	48.8	56.5	2.5852	0.1079	48.5	55.6	2.2155	0.1366
3	17	2	53.4	62.8	4.9914	0.0255	51.8	62.6	6.5790	0.0103
3	18	1	24.9	22.5	0.3601	0.5485	26.1	23.4	0.4531	0.5009
3	18	2	39.5	45.1	2.2194	0.1363	38.2	43.7	2.1362	0.1439
3	19	1	22.6	14.4	3.4821	0.0620	23.5	14.5	4.0983	0.0429
3	19	2	45.2	40.7	1.1508	0.2834	43.4	39.4	0.6771	0.4106
3	20	1	40.7	37.8	0.4565	0.4993	44.1	39.5	1.1927	0.2748
3	20	2	51.9	57.1	1.8538	0.1733	50.0	55.9	2.3539	0.1250
3	21	1	40.1	30.4	3.6953	0.0546	43.6	31.2	6.0749	0.0137
3	21	2	58.1	52.8	1.7277	0.1887	55.6	51.6	0.8220	0.3646
3	22	1	65.1	59.3	1.5561	0.2122	65.5	59.4	1.7643	0.1841
3	22	2	62.4	68.4	2.2445	0.1341	60.2	68.0	3.6903	0.0547
3	23	1	65.0	49.0	8.3301	0.0039	62.5	47.0	7.8078	0.0052
3	23	2	68.8	63.0	1.8993	0.1682	68.0	62.5	1.5725	0.2098
4	24	1	61.2	75.1	9.2638	0.0023	62.2	75.2	8.2048	0.0042
4	24	2	65.8	74.4	4.8055	0.0284	63.5	73.0	5.6444	0.0175
4	25	1	11.5	12.9	0.2096	0.6471	11.1	12.5	0.2064	0.6496
4	25	2	20.7	18.4	0.4319	0.5111	19.3	19.1	0.0019	0.9656
4	26	1	62.7	76.6	9.4840	0.0021	63.5	76.7	8.6936	0.0032
4	26	2	68.4	75.9	3.8007	0.0512	66.9	74.3	3.5169	0.0607
4	27	1	42.1	47.8	1.4400	0.2301	42.3	47.6	1.2194	0.2695
4	27	2	54.5	62.4	3.4903	0.0617	52.6	61.1	4.0230	0.0449

For peer review only

Table S2. Indicators from mothers two samples in Aurangabad and Gopalganj of mothers of infants aged 0-2 months compared to 0-5 months: Estimates, Cochran-Mantel-Haenszel Chi-squares, and probabilities: Bihar 2015 - Continued										
			Unweighted				Weighted			
Domain[a]	Indicator[b]	District[c]	Percent 0-2	Percent 0-5	Chi-square	Probability	Percent 0-2	Percent 0-5	Chi-square	Probability
4	28	1	8.6	8.1	0.0323	0.8575	8.2	7.6	0.0561	0.8127
4	28	2	16.2	13.9	0.5345	0.4647	15.4	13.8	0.2732	0.6012
4	29	1	43.1	48.8	1.4361	0.2308	43.3	48.5	1.1846	0.2764
4	29	2	57.5	64.7	2.9183	0.0876	55.9	63.5	3.2352	0.0721
4	30	1	29.7	35.4	1.6374	0.2007	28.7	34.2	1.5497	0.2132
4	30	2	48.1	53	1.2686	0.2600	46.9	51.4	1.0908	0.2963
4	31	1	30.1	45.9	10.9581	0.0000	29.9	44.9	9.9713	0.0016
4	31	2	47.4	53.0	1.6849	0.1943	44.5	52.7	3.6136	0.0573
4	32	1	5.3	3.8	0.4948	0.4818	5.3	3.6	0.7401	0.3896
4	32	2	9.8	9.0	0.0897	0.7646	9.1	9.9	0.0974	0.7550
4	33	1	32.5	47.8	10.1305	0.0015	32.2	46.7	9.0939	0.0026
4	33	2	50.8	56.4	1.7133	0.1906	48.2	56.2	3.5264	0.0604
4	34	1	6.2	10.0	2.0808	0.1492	6.2	9.9	1.9617	0.1613
4	34	2	13.5	9.0	2.7721	0.0959	14.4	8.9	3.9431	0.0471
4	35	1	45.0	60.3	9.7398	0.0018	44.5	59.3	9.0930	0.0026
4	35	2	64.7	69.5	1.4491	0.2287	62.5	69.0	2.5201	0.1124
5	37	1	73.2	76.6	0.6496	0.4203	72.9	74.9	0.2182	0.6404
5	37	2	80.1	86.1	3.4220	0.0643	78.9	85.5	3.9858	0.0459
5	38	1	55.5	56.5	0.0389	0.8436	54.7	55.3	0.0117	0.9139
5	38	2	52.3	61.3	4.3834	0.0363	51.2	61.6	5.8090	0.0159
5	39	1	5.7	0	3.3034	0.0691	5.4	0	3.6637	0.0556
5	39	2	10.2	8.3	0.2393	0.6247	9.1	6.6	0.3451	0.5569
5	39.5	1	1.4	0	0.0003	0.0833	1.4	0	2.8978	0.0887
5	39.5	2	1.9	1.1	0.5139	0.4735	1.8	0.9	0.7629	0.3824
6	42	1	42.6	47.4	1.0165	0.3134	43.8	47.6	0.6553	0.4182
6	42	2	41.4	46.2	1.3134	0.2518	41.8	46.3	1.1228	0.2893
6	43	1	24.9	29.7	1.3262	0.2495	25.6	30.4	1.3008	0.2541
6	43	2	27.1	29.7	0.4968	0.4809	26.9	31.2	1.2586	0.2619

For peer review only

Table S2. Indicators from mothers two samples in Aurangabad and Gopalganj of mothers of infants aged 0-2 months compared to 0-5 months: Estimates, Cochran-Mantel-Haenszel Chi-squares, and probabilities: Bihar 2015 - Continued

			Unweighted				Weighted			
Domain[a]	Indicator[b]	District[c]	Percent 0-2	Percent 0-5	Chi-square	Probability	Percent 0-2	Percent 0-5	Chi-square	Probability
6	44	1	9.8	12.5	1.2833	0.2573	12.2	13.0	0.3958	0.5293
6	44	2	21.1	20.1	0.0586	0.8087	21.6	18.7	0.5178	0.4718
6	45	1	7.5	10.2	0.6321	0.4266	7.8	11.1	0.8502	0.3565
6	45	2	8.2	5.6	0.4009	0.5266	5.2	8.3	0.1670	0.6828
6	46	1	55.5	58.9	0.4904	0.4837	56.6	59.2	0.2960	0.5864
6	46	2	62.8	68.0	1.6764	0.1954	61.7	66.3	1.2736	0.2591
6	47	1	34.0	32.1	0.1768	0.6741	35.9	32.8	0.4369	0.5086
6	47	2	67.3	66.5	0.0344	0.8528	66.6	68.3	0.1745	0.6762
6	48	1	65.6	67.5	0.1782	0.6729	64.9	66.6	0.1312	0.7172
6	48	2	71.1	77.8	3.2208	0.0727	70.7	78.2	3.9664	0.0464
6	49	1	62.7	59.4	0.1537	0.6950	64.5	61.2	0.1691	0.6809
6	49	2	68.1	67.2	0.0166	0.8974	67.3	66.8	0.0011	0.9735
6	50	1	49.0	55.0	1.2527	0.2630	48.4	54.7	1.3188	0.2508
6	50	2	56.8	55.9	0.0964	0.7562	56.1	55.5	0.0615	0.8042
6	51	1	81.1	44.9	14.9736	0.0000	78.0	45.4	11.7395	0.0000
6	51	2	63.3	38.9	4.0901	0.0431	63.7	41.1	3.4654	0.0627
7	52	1	70.3	59.8	5.1727	0.0229	69.2	59.7	4.1723	0.0411
7	52	2	82.3	67.3	16.2014	0.0000	82.1	68.4	13.3711	0.0000

a . 1 Antenatal care, 2 Maternal health, 3 Birth preparedness, 4 FLW Support, 5 Place of birth & attendant, 6 Neonatal Health, 7 Exclusive breastfeeding

b . For text see Table S1

c. 1 Aurangabad, 2 Gopalganj

For peer review only

Table S3. Weighted indicators and confidence intervals from two samples in two districts of Bihar

Indicator[a]	District[b]	0-2 Month Sample			0-5 Month Sample			Difference		
		Point estimate	Confidence interval[c]		Point estimate	Confidence interval[c]		Point estimate	Confidence interval	
			Lower	Upper		Lower	Upper		Lower	Upper
1	1	85.2	80.2	90.2	77.6	72.6	82.6	7.6	0.1	15.2
1	2	85.0	80.5	89.6	85.4	80.9	90.0	-0.4	-6.7	5.9
2	1	43.0	36.3	49.8	38.3	31.5	45.0	4.7	-4.5	14.0
2	2	47.5	41.5	53.6	48.2	42.1	54.2	-0.6	-9.5	8.2
3	1	83.8	78.8	88.8	80.4	75.4	85.4	3.4	-3.6	10.4
3	2	80.6	75.7	85.5	77.8	72.9	82.7	2.8	-4.5	10.0
4	1	61.1	54.4	67.8	63.1	56.4	69.9	-2.0	-11.4	7.3
4	2	57.4	51.2	63.6	61.0	54.8	67.2	-3.5	-12.3	5.2
5	1	43.2	36.4	50.1	45.7	38.9	52.6	-2.5	-12.2	7.2
5	2	43.4	37.1	49.8	46.9	40.5	53.2	-3.4	-12.4	5.6
6	1	58.9	52.3	65.6	60.1	53.5	66.7	-1.2	-10.4	8.0
6	2	66.3	60.2	72.4	63.3	57.2	69.4	2.9	-5.7	11.6
7	1	42.0	35.3	48.8	39.5	32.7	46.3	2.5	-7.0	12.1
7	2	69.2	63.2	75.2	72.6	66.6	78.6	-3.4	-11.7	4.9
8	1	42.7	36.1	49.3	44.8	38.2	51.4	-2.1	-11.4	7.2
8	2	52.4	46.2	58.7	54.5	48.2	60.8	-2.1	-10.9	6.7
9	1	45.8	39.0	52.6	41.7	34.9	48.4	4.1	-5.4	13.6
9	2	42.8	36.6	49.1	43.5	37.3	49.7	-0.6	-9.5	8.2
10	1	67.2	60.9	73.6	59.6	53.2	65.9	7.6	-1.2	16.5
10	2	54.8	48.5	61.1	58.5	52.2	64.8	-3.7	-12.5	5.2
11	1	98.5	96.7	100.2	98.1	96.4	99.9	0.4	-2.2	2.9
11	2	96.9	94.8	99.1	97.1	94.9	99.3	-0.2	-3.0	2.7
12	1	69.3	63.0	75.7	67.5	61.2	73.9	1.8	-7.2	10.8
12	2	81.9	77.3	86.6	80.4	75.7	85.0	1.6	-5.2	8.4
13	1	4.0	1.2	6.7	2.6	-0.1	5.4	1.3	-2.2	4.9
13	2	3.0	1.0	5.0	5.1	3.1	7.1	-2.2	-5.5	1.2
14	1	1.6	-0.2	3.4	2.0	0.2	3.8	-0.4	-3.1	2.3
14	2	1.7	0.2	3.2	3.6	2.1	5.1	-1.9	-4.7	0.9
15	1	25.9	20.1	31.6	33.3	27.5	39.0	-7.4	-15.9	1.1
15	2	45.7	39.6	51.8	56.0	49.9	62.1	-10.3	-19.0	-1.5
16	1	4.3	1.5	7.0	3.6	0.8	6.3	0.7	-3.0	4.4
16	2	9.7	6.1	13.3	14.3	10.8	17.9	-4.7	-10.5	1.1
17	1	48.5	41.8	55.2	55.6	48.9	62.3	-7.1	-16.5	2.4
17	2	51.8	45.6	58.0	62.6	56.4	68.8	-10.8	-19.6	-2.1
18	1	26.1	20.2	32.1	23.4	17.5	29.4	2.7	-5.5	10.9
18	2	38.2	33.0	43.3	43.7	38.5	48.8	-5.5	-13.2	2.2
19	1	23.5	16.4	30.7	14.5	7.3	21.7	9.1	0.4	17.7
19	2	43.4	37.3	49.5	39.4	33.3	45.5	4.0	-4.6	12.6
20	1	44.1	38.3	50.0	39.5	33.7	45.4	4.6	-3.7	12.9
20	2	50.0	44.7	55.4	55.9	50.5	61.3	-5.9	-13.7	2.0
21	1	43.6	36.7	50.5	31.2	24.3	38.1	12.4	3.3	21.5
21	2	55.6	49.5	61.7	51.6	45.5	57.7	4.0	-4.6	12.6
22	1	65.5	59.0	72.0	59.4	52.9	65.9	6.1	-3.0	15.2
22	2	60.2	54.0	66.3	68.0	61.8	74.2	-7.8	-16.4	0.7
23	1	62.5	54.9	70.2	47.0	39.3	54.7	15.5	5.4	25.7
23	2	68.0	61.2	74.8	62.5	55.7	69.3	5.5	-3.8	14.8

For peer review only

24	1	62.2	55.5	68.9	75.2	68.5	81.9	-13.0	-22.0	-4.1
24	2	63.5	57.4	69.7	73.0	66.9	79.2	-9.5	-17.9	-1.1
25	1	11.1	7.0	15.1	12.5	8.4	16.5	-1.4	-7.5	4.7
25	2	19.3	14.4	24.2	19.1	14.3	24.0	0.1	-6.9	7.2
26	1	63.5	56.8	70.1	76.7	70.1	83.4	-13.2	-22.1	-4.4
26	2	66.9	60.9	73.0	74.3	68.3	80.3	-7.4	-15.7	1.0
27	1	42.3	35.6	49.1	47.6	40.8	54.4	-5.3	-14.8	4.3
27	2	52.6	46.3	58.9	61.1	54.8	67.4	-8.5	-17.2	0.3
28	1	8.2	4.6	11.8	7.6	4.0	11.2	0.6	-4.4	5.7
28	2	15.4	11.0	19.8	13.8	9.4	18.3	1.6	-4.7	7.8
29	1	43.3	36.5	50.1	48.5	41.7	55.3	-5.2	-14.8	4.3
29	2	55.9	49.7	62.2	63.5	57.2	69.8	-7.5	-16.3	1.2
30	1	28.7	22.6	34.7	34.2	28.1	40.2	-5.5	-14.3	3.2
30	2	46.9	40.5	53.3	51.4	45.0	57.8	-4.5	-13.5	4.5
31	1	29.9	23.5	36.3	44.9	38.5	51.3	-15.0	-24.4	-5.6
31	2	44.5	38.2	50.8	52.7	46.5	59.0	-8.2	-17.2	0.7
32	1	5.3	2.2	8.5	3.6	0.4	6.7	1.7	-2.3	5.8
32	2	9.1	5.7	12.6	9.9	6.4	13.4	-0.8	-6.0	4.4
33	1	32.2	25.8	38.7	46.7	40.2	53.2	-14.5	-23.9	-5.0
33	2	48.2	41.9	54.5	56.2	49.9	62.5	-8.1	-16.9	0.8
34	1	6.2	2.8	9.6	9.9	6.5	13.3	-3.7	-8.9	1.5
34	2	14.4	9.8	19.0	8.9	4.4	13.5	5.5	-0.3	11.3
35	1	44.5	37.7	51.4	59.3	52.5	66.2	-14.8	-24.4	-5.1
35	2	62.5	56.4	68.7	69.0	62.9	75.2	-6.5	-15.1	2.1
37	1	72.9	66.7	79.1	74.9	68.7	81.0	-2.0	-10.5	6.5
37	2	78.9	73.5	84.2	85.5	80.2	90.8	-6.6	-13.7	0.4
38	1	54.7	47.8	61.7	55.3	48.4	62.2	-0.5	-10.3	9.2
38	2	51.2	44.7	57.6	61.6	55.2	68.0	-10.4	-19.4	-1.5
39	1	5.4	-0.9	11.8	0.0	-6.4	6.4	5.4	-0.9	11.8
39	2	9.1	1.3	16.9	6.6	-1.2	14.4	2.5	-9.1	14.1
39.5	1	1.4	-0.2	3.0	0.0	-1.6	1.6	1.4	-0.2	3.0
39.5	2	1.8	0.2	3.4	0.9	-0.6	2.5	0.9	-1.0	2.8
40	1	10.6	6.5	14.7	16.0	12.0	20.1	-5.4	-11.7	0.8
40	2	8.5	4.9	12.2	9.5	5.8	13.1	-0.9	-5.9	4.1
42	1	43.8	36.9	50.6	47.6	40.7	54.5	-3.8	-13.3	5.6
42	2	41.8	35.6	48.1	46.3	40.1	52.6	-4.5	-13.4	4.4
43	1	25.6	19.8	31.5	30.4	24.6	36.2	-4.8	-13.3	3.7
43	2	26.9	21.5	32.4	31.2	25.7	36.6	-4.2	-12.0	3.5
44	1	12.2	7.3	17.1	13.0	8.1	17.9	-0.8	-7.7	6.1
44	2	21.6	16.3	26.8	18.7	13.4	24.0	2.8	-4.4	10.1
45	1	7.8	0.4	15.2	11.1	3.7	18.5	-3.3	-16.2	9.6
45	2	5.2	0.3	10.1	8.3	3.4	13.2	-3.1	-17.4	11.2
46	1	56.6	49.9	63.2	59.2	52.5	65.8	-2.6	-12.2	7.0
46	2	61.7	55.5	67.9	66.3	60.1	72.6	-4.6	-13.3	4.1
47	1	35.9	29.3	42.5	32.8	26.2	39.5	3.0	-6.2	12.3
47	2	66.6	60.6	72.7	68.3	62.3	74.4	-1.7	-10.1	6.7
48	1	64.9	58.5	71.4	66.6	60.1	73.0	-1.6	-10.8	7.5
48	2	70.7	64.9	76.5	78.2	72.4	84.0	-7.5	-15.2	0.2
49	1	64.5	57.1	71.8	61.2	53.9	68.5	3.3	-6.7	13.3
49	2	67.3	60.6	73.9	66.8	60.1	73.4	0.5	-8.9	9.9
50	1	48.4	40.3	56.4	54.7	46.7	62.8	-6.4	-17.4	4.7
50	2	56.1	49.0	63.2	55.5	48.4	62.6	0.7	-9.2	10.5

For peer review only

51	1	78.0	65.9	90.2	45.4	33.3	57.6	32.6	13.0	52.2
51	2	63.7	48.8	78.5	41.1	26.2	56.0	22.5	-2.2	47.2
52	1	69.2	62.7	75.6	59.7	53.3	66.1	9.5	0.2	18.8
52	2	82.1	77.1	87.0	68.4	63.5	73.4	13.6	6.0	21.2

a. For text see Table S1
b. 1 Aurangabad, 2 Gopalganj
c. Estimated with Stata command svy

For peer review only

Table S4a: Point estimates and confidence intervals of indicators for subsamples of 0-2 and 3-5 month old infants in two districts: Bihar, India, 2015

Indicator[a]	District[b]	0-2 month subsample			3-5 month subsample			Difference		
		Point estimate	Confidence interval[c]		Point estimate	Confidence interval[c]		Point estimate	Confidence interval	
			Lower	Upper		Lower	Upper		Lower	Upper
1	1	79.2	71.9	86.5	75.2	66.0	84.4	4.0	-7.7	15.7
1	2	80.8	74.4	87.2	92.3	87.5	97.2	-11.5	-19.5	-3.5
2	1	36.1	27.8	44.4	41.5	31.7	51.3	-5.4	-18.2	7.4
2	2	46.5	38.1	54.8	50.6	40.3	61.0	-4.2	-17.5	9.1
3	1	81.0	75.0	87.1	79.5	71.5	87.5	1.5	-8.5	11.6
3	2	79.9	73.4	86.5	74.7	65.8	83.7	5.2	-5.9	16.3
4	1	61.8	53.1	70.4	65.2	55.0	75.3	-3.4	-16.7	10.0
4	2	62.7	54.7	70.7	58.4	48.4	68.3	4.4	-8.4	17.1
5	1	45.0	36.2	53.9	46.8	35.5	58.0	-1.7	-16.0	12.6
5	2	49.1	40.8	57.4	43.5	33.3	53.7	5.6	-7.6	18.7
6	1	60.3	51.9	68.7	59.8	49.6	70.0	0.5	-12.7	13.7
6	2	68.5	60.8	76.1	55.8	45.7	65.8	12.7	0.0	25.4
7	1	40.3	31.8	48.8	38.4	27.4	49.3	1.9	-11.9	15.8
7	2	74.5	67.2	81.9	69.8	60.3	79.3	4.7	-7.3	16.8
8	1	49.3	41.2	57.3	38.2	27.9	48.5	11.1	-2.0	24.2
8	2	60.0	52.0	67.9	46.4	36.7	56.2	13.6	1.0	26.1
9	1	39.8	31.3	48.4	44.4	33.9	54.8	-4.5	-18.0	9.0
9	2	44.7	36.6	52.8	41.7	31.5	51.9	3.0	-10.0	16.0
10	1	61.9	54.4	69.4	56.1	45.3	66.9	5.7	-7.4	18.9
10	2	63.9	56.1	71.7	50.5	40.3	60.6	13.5	0.7	26.2
11	1	98.4	96.3	100.6	97.6	94.3	100.9	0.8	-3.1	4.8
11	2	96.7	94.1	99.3	97.7	95.1	100.3	-1.0	-4.7	2.7
12	1	65.0	56.4	73.6	71.3	61.8	80.7	-6.3	-19.1	6.5
12	2	77.8	71.1	84.6	84.1	76.7	91.4	-6.2	-16.2	3.8
13	1	3.3	0.1	6.5	1.6	-1.5	4.8	1.7	-2.8	6.2
13	2	6.7	2.6	10.9	2.8	0.0	5.6	3.9	-1.1	8.9
14	1	2.2	-0.4	4.8	1.6	-1.5	4.8	0.6	-3.5	4.7
14	2	4.5	1.0	8.0	2.3	-0.4	4.9	2.2	-2.2	6.6
15	1	35.2	26.8	43.6	30.4	20.9	39.9	4.8	-7.8	17.5
15	2	56.8	48.7	64.9	54.8	44.3	65.3	2.0	-11.3	15.2
16	1	5.3	1.4	9.3	0.9	-0.8	2.6	4.5	0.2	8.7
16	2	16.6	10.2	23.0	11.0	4.8	17.1	5.7	-3.2	14.5
17	1	59.6	51.4	67.9	49.5	38.7	60.3	10.1	-3.5	23.7
17	2	64.4	56.5	72.4	59.9	49.8	70.0	4.5	-8.3	17.4
18	1	24.2	16.8	31.6	22.2	13.9	30.5	2.0	-9.1	13.1
18	2	45.9	38.1	53.8	40.3	32.0	48.6	5.7	-5.7	17.1
19	1	17.9	11.2	24.5	9.2	2.4	16.1	8.6	-0.9	18.2
19	2	40.5	32.4	48.6	37.7	29.0	46.4	2.8	-9.0	14.6
20	1	42.5	34.8	50.2	35.1	26.0	44.2	7.4	-4.5	19.4
20	2	59.4	51.7	67.0	50.7	42.3	59.1	8.6	-2.7	20.0
21	1	37.0	29.2	44.8	22.1	13.1	31.2	14.9	2.9	26.8
21	2	54.2	46.1	62.3	47.7	38.9	56.6	6.5	-5.6	18.5
22	1	62.5	54.0	70.9	54.7	44.8	64.6	7.8	-5.2	20.8
22	2	69.7	62.3	77.1	65.5	55.4	75.5	4.2	-8.2	16.7
23	1	47.5	39.1	55.9	46.1	35.0	57.3	1.4	-12.5	15.3

For peer review only

23	2	64.9	56.9	73.0	58.9	48.4	69.5	6.0	-7.3	19.3
24	1	75.1	67.4	82.9	75.3	66.0	84.7	-0.2	-12.4	11.9
24	2	67.6	59.7	75.5	81.0	72.7	89.3	-13.4	-24.8	-1.9
25	1	12.1	6.1	18.0	13.1	5.9	20.2	-1.0	-10.3	8.3
25	2	16.7	10.3	23.1	22.7	13.8	31.6	-6.0	-16.9	4.9
26	1	76.2	68.6	83.8	77.5	68.5	86.6	-1.3	-13.1	10.5
26	2	69.4	61.6	77.2	81.6	73.3	89.8	-12.2	-23.6	-0.8
27	1	50.4	41.5	59.3	43.4	33.0	53.9	6.9	-6.8	20.7
27	2	57.2	48.9	65.4	66.9	57.7	76.0	-9.7	-22.0	2.7
28	1	8.6	3.7	13.6	6.0	1.3	10.6	2.7	-4.1	9.5
28	2	9.8	5.1	14.5	19.7	11.4	28.1	-9.9	-19.5	-0.3
29	1	50.4	41.5	59.3	45.6	35.4	55.9	4.8	-8.8	18.4
29	2	59.2	51.1	67.4	69.7	60.5	79.0	-10.5	-22.9	1.8
30	1	32.7	24.5	41.0	36.3	25.9	46.7	-3.6	-16.9	9.7
30	2	49.2	41.0	57.4	54.7	44.3	65.1	-5.5	-18.8	7.7
31	1	42.0	33.0	51.1	49.2	39.0	59.5	-7.2	-20.9	6.5
31	2	48.6	40.3	56.8	58.9	48.8	69.0	-10.3	-23.3	2.7
32	1	3.9	0.4	7.3	3.2	-0.4	6.8	0.7	-4.3	5.7
32	2	9.4	4.2	14.5	10.7	4.7	16.8	-1.4	-9.3	6.5
33	1	43.6	34.5	52.6	51.4	41.1	61.7	-7.8	-21.6	5.9
33	2	52.9	44.8	61.0	61.1	51.1	71.2	-8.2	-21.1	4.7
34	1	7.0	2.5	11.5	14.2	7.0	21.4	-7.2	-15.7	1.2
34	2	8.6	3.8	13.4	9.5	4.0	15.0	-0.9	-8.2	6.4
35	1	57.2	48.2	66.2	62.5	52.7	72.4	-5.3	-18.7	8.0
35	2	63.2	55.1	71.4	77.6	68.6	86.6	-14.3	-26.5	-2.2
37	1	76.6	69.1	84.1	72.3	62.7	81.8	4.3	-7.8	16.4
37	2	81.6	74.9	88.3	91.3	85.8	96.8	-9.7	-18.4	-1.0
38	1	59.0	49.9	68.0	49.7	39.3	60.2	9.2	-4.6	23.1
38	2	56.1	47.8	64.4	69.7	61.2	78.2	-13.6	-25.5	-1.7
39	1	0.0	0.0	0.0	0.0	0.0	0.0	0.0	0.0	0.0
39	2	5.8	-3.1	14.7	9.6	-40.1	59.3	-3.8	-54.3	46.7
39.5	1	0.0	0.0	0.0	0.0	0.0	0.0	0.0	0.0	0.0
39.5	2	1.1	-0.4	2.6	0.7	-0.7	2.1	0.3	-1.7	2.4
40	1	17.6	10.9	24.4	13.6	7.5	19.8	4.0	-5.2	13.2
40	2	8.0	3.9	12.1	11.6	5.6	17.7	-3.7	-11.0	3.6
42	1	52.6	44.3	61.0	40.1	29.6	50.6	12.5	-0.9	25.9
42	2	47.5	39.4	55.6	44.6	34.4	54.8	2.9	-10.2	16.0
43	1	35.6	27.4	43.8	22.6	13.6	31.5	13.1	0.9	25.2
43	2	35.5	28.0	43.0	24.8	16.4	33.1	10.8	-0.5	22.0
44	1	10.1	4.2	16.0	17.6	9.5	25.7	-7.5	-17.6	2.5
44	2	20.6	14.1	27.0	16.2	8.7	23.8	4.3	-5.6	14.3
45	1	9.0	-4.7	22.7	13.7	-0.7	28.1	-4.7	-24.6	15.2
45	2	6.6	-9.9	23.2	14.4	14.4	14.4	-7.7	-24.3	8.8
46	1	63.4	54.6	72.2	52.8	41.5	64.0	10.7	-3.6	24.9
46	2	68.9	61.1	76.7	62.5	52.6	72.4	6.4	-6.2	19.0
47	1	33.0	24.7	41.4	32.5	22.2	42.9	0.5	-12.8	13.8
47	2	70.4	62.8	77.9	65.3	55.9	74.8	5.1	-7.1	17.2
48	1	68.8	60.6	77.0	63.2	52.5	73.8	5.7	-7.8	19.1
48	2	76.2	69.3	83.0	81.3	73.9	88.6	-5.1	-15.2	5.0
49	1	66.5	57.9	75.1	52.7	41.0	64.3	13.8	-0.6	28.3
49	2	68.6	59.9	77.3	64.4	53.8	75.0	4.2	-9.5	17.9
50	1	51.7	42.1	61.4	59.5	47.6	71.5	-7.8	-23.1	7.6

For peer review only

50	2	55.2	46.3	64.1	55.8	45.1	66.6	-0.7	-14.6	13.3
51	1	33.2	14.6	51.7	61.0	36.7	85.4	-27.9	-58.5	2.7
51	2	40.6	17.5	63.7	43.0	-6.7	92.7	-2.4	-57.2	52.4
52	1	73.0	65.1	80.9	39.7	28.6	50.7	33.3	19.8	46.9
52	2	83.8	78.2	89.3	45.8	35.6	55.9	38.0	26.4	49.5

a. For text see Table S1
b. 1 Aurangabad, 2 Gopalganj
c. Estimated with Stata command svy

For peer review only

Table S4b: Indicators in Table S4a where zero is not in the estimated confidence interval of difference between 0-2 and 3-5 month subsamples							
	Indicator	District[a]	Point estimate		Difference	Confidence interval[b]	
			0-2 month	3-5 month		Lower	Upper
			In Table S4a and in Table S2				
21	Proportion of mothers who planned institutional delivery of infants (0-2/0-5) months who arranged clean cloth for mothers and baby	1	37.0	22.1	14.9	2.9	26.8
24	Proportion of mothers of infants (0-2/0-5) months who were visited by ASHA at least once during their last pregnancy	2	67.6	81.0	-13.4	-24.8	-1.9
37	Proportion of mothers of infants (0-2/0-5) months whose last child was delivered at a health facility (private or public facility)	2	81.6	91.3	-9.7	-18.4	-1.0
38	Proportion of mothers of infants (0-2/0-5) months whose last child was delivered at public facility	2	56.1	69.7	-13.6	-25.5	-1.7
52	Proportion of infants (0-2/0-5) months who were breast-fed in the past	1	73.0	39.7	33.3	19.8	46.9
52	24 hours (Exclusively Breast-fed)	2	83.8	45.8	38.0	26.4	49.5
			In Table S4a but not in Table S2				
1	Proportion of mothers of infants (0-2/0-5) months who were registered during their last pregnancy	2	80.8	92.3	-11.5	-19.5	-3.5
8	Proportion of mothers of infants (0-2/0-5) months who attended at least one ANC where at least one abdominal examination was performed during her last pregnancy	2	60.0	46.4	13.6	1.0	26.1
10	Proportion of mothers of infants (0-2/0-5) months who attended at least one ANC where at least one blood test was performed during her last pregnancy	2	63.9	50.5	13.5	0.7	26.2
16	Proportion of mothers (home + institutional delivery) of infants (0-2/0-5) months who have identified anybody who would donate blood in the case of emergency in their last pregnancy	1	5.3	0.9	4.5	0.2	8.7
26	Proportion of mothers of infants (0-2/0-5) months who were visited by FLWs at least once during their last pregnancy	2	69.4	81.6	-12.2	-23.6	-0.8
28	Proportion of mothers of infants (0-2/0-5) months who were visited by AWW in the last trimester during their last pregnancy	2	9.8	19.7	-9.9	-19.5	-0.3
35	Proportion of mothers of infants (0-2/0-5) months who were visited home by any FLW within first week of last delivery	2	63.2	77.6	-14.3	-26.5	-2.2

For peer review only

43	Proportion of mothers (home + institutional delivery) of infants (0-2/0-5) months who have delivered baby practiced skin to skin care (STSC) immediately after birth	1	35.6	22.6	13.1	0.9	25.2

a. 1 = Aurangabad; 2 = Gopalganj

b. Calculated from standard errors for point estimates estimated with Stata command svy

For peer review only

Table S5a: Point estimates and confidence intervals of indicators for a sample 0-2 month old infants and a subsample of 0-2 month old infants from a sample of 0-5 month old infants in two districts: Bihar, India, 2015

Indicator[a]	District[b]	0-2 month subsample			0-2 month sample			Difference		
		Point estimate	Confidence interval[c]		Point estimate	Confidence interval[c]		Point estimate	Confidence interval	
			Lower	Upper		Lower	Upper		Lower	Upper
1	1	79.2	71.9	86.5	85.2	80.2	90.2	-6.0	-14.8	2.8
1	2	80.8	74.4	87.2	85.0	80.5	89.6	-4.2	-12.1	3.6
2	1	36.1	27.8	44.4	43.0	36.3	49.8	-6.9	-17.6	3.8
2	2	46.5	38.1	54.8	47.5	41.5	53.6	-1.1	-11.3	9.2
3	1	81.0	75.0	87.1	83.8	78.8	88.8	-2.8	-10.7	5.1
3	2	79.9	73.4	86.5	80.6	75.7	85.5	-0.7	-8.8	7.5
4	1	61.8	53.1	70.4	61.1	54.4	67.8	0.7	-10.3	11.6
4	2	62.7	54.7	70.7	57.4	51.2	63.6	5.3	-4.8	15.4
5	1	45.0	36.2	53.9	43.2	36.4	50.1	1.8	-9.4	13.0
5	2	49.1	40.8	57.4	43.4	37.1	49.8	5.7	-4.8	16.2
6	1	60.3	51.9	68.7	58.9	52.3	65.6	1.4	-9.3	12.1
6	2	68.5	60.8	76.1	66.3	60.2	72.4	2.2	-7.6	12.0
7	1	40.3	31.8	48.8	42.0	35.3	48.8	-1.7	-12.6	9.2
7	2	74.5	67.2	81.9	69.2	63.2	75.2	5.3	-4.2	14.8
8	1	49.3	41.2	57.3	42.7	36.1	49.3	6.5	-3.9	16.9
8	2	60.0	52.0	67.9	52.4	46.2	58.7	7.6	-2.6	17.7
9	1	39.8	31.3	48.4	45.8	39.0	52.6	-5.9	-16.8	5.0
9	2	44.7	36.6	52.8	42.8	36.6	49.1	1.8	-8.3	12.0
10	1	61.9	54.4	69.4	67.2	60.9	73.6	-5.3	-15.2	4.5
10	2	63.9	56.1	71.7	54.8	48.5	61.1	9.1	-0.9	19.1
11	1	98.4	96.3	100.6	98.5	96.7	100.2	-0.0	-2.8	2.8
11	2	96.7	94.1	99.3	96.9	94.8	99.1	-0.2	-3.7	3.2
12	1	65.0	56.4	73.6	69.3	63.0	75.7	-4.3	-15.0	6.4
12	2	77.8	71.1	84.6	81.9	77.3	86.6	-4.1	-12.3	4.1
13	1	3.3	0.1	6.5	4.0	1.2	6.7	-0.6	-4.9	3.6
13	2	6.7	2.6	10.9	3.0	1.0	5.0	3.7	-0.9	8.3
14	1	2.2	-0.4	4.8	1.6	-0.2	3.4	0.7	-2.5	3.8
14	2	4.5	1.0	8.0	1.7	0.2	3.2	2.8	-1.1	6.7
15	1	35.2	26.8	43.6	25.9	20.1	31.6	9.3	-0.8	19.5
15	2	56.8	48.7	64.9	45.7	39.6	51.8	11.1	0.9	21.2
16	1	5.3	1.4	9.3	4.3	1.5	7.0	1.1	-3.7	5.9
16	2	16.6	10.2	23.0	9.7	6.1	13.3	7.0	-0.4	14.3
17	1	59.6	51.4	67.9	48.5	41.8	55.2	11.1	0.5	21.7
17	2	64.4	56.5	72.4	51.8	45.6	58.0	12.7	2.6	22.7
18	1	24.2	16.8	31.6	26.1	20.2	32.1	-1.9	-11.4	7.6
18	2	45.9	38.1	53.8	38.2	33.0	43.3	7.8	-1.6	17.1
19	1	17.9	11.2	24.5	23.5	16.4	30.7	-5.7	-15.5	4.1
19	2	40.5	32.4	48.6	43.4	37.3	49.5	-2.9	-13.0	7.3
20	1	42.5	34.8	50.2	44.1	38.3	50.0	-1.6	-11.3	8.0
20	2	59.4	51.7	67.0	50.0	44.7	55.4	9.3	0.0	18.7
21	1	37.0	29.2	44.8	43.6	36.7	50.5	-6.6	-17.0	3.8
21	2	54.2	46.1	62.3	55.6	49.5	61.7	-1.4	-11.6	8.7
22	1	62.5	54.0	70.9	65.5	59.0	72.0	-3.0	-13.7	7.7
22	2	69.7	62.3	77.1	60.2	54.0	66.3	9.5	-0.1	19.2

For peer review only

23	1	47.5	39.1	55.9	62.5	54.9	70.2	-15.0	-26.4	-3.6
23	2	64.9	56.9	73.0	68.0	61.2	74.8	-3.1	-13.7	7.4
24	1	75.1	67.4	82.9	62.2	55.5	68.9	12.9	2.7	23.2
24	2	67.6	59.7	75.5	63.5	57.4	69.7	4.1	-5.9	14.1
25	1	12.1	6.1	18.0	11.1	7.0	15.1	1.0	-6.2	8.2
25	2	16.7	10.3	23.1	19.3	14.4	24.2	-2.6	-10.6	5.5
26	1	76.2	68.6	83.8	63.5	56.8	70.1	12.7	2.6	22.8
26	2	69.4	61.6	77.2	66.9	60.9	73.0	2.4	-7.4	12.3
27	1	50.4	41.5	59.3	42.3	35.6	49.1	8.1	-3.1	19.3
27	2	57.2	48.9	65.4	52.6	46.3	58.9	4.6	-5.8	15.0
28	1	8.6	3.7	13.6	8.2	4.6	11.8	0.5	-5.7	6.6
28	2	9.8	5.1	14.5	15.4	11.0	19.8	-5.6	-12.0	0.8
29	1	50.4	41.5	59.3	43.3	36.5	50.1	7.1	-4.1	18.3
29	2	59.2	51.1	67.4	55.9	49.7	62.2	3.3	-7.0	13.6
30	1	32.7	24.5	41.0	28.7	22.6	34.7	4.1	-6.1	14.3
30	2	49.2	41.0	57.4	46.9	40.5	53.3	2.3	-8.1	12.7
31	1	42.0	33.0	51.1	29.9	23.5	36.3	12.1	1.1	23.2
31	2	48.6	40.3	56.8	44.5	38.2	50.8	4.1	-6.3	14.5
32	1	3.9	0.4	7.3	5.3	2.2	8.5	-1.5	-6.2	3.2
32	2	9.4	4.2	14.5	9.1	5.7	12.6	0.2	-6.0	6.4
33	1	43.6	34.5	52.6	32.2	25.8	38.7	11.3	0.2	22.5
33	2	52.9	44.8	61.0	48.2	41.9	54.5	4.7	-5.5	15.0
34	1	7.0	2.5	11.5	6.2	2.8	9.6	0.8	-4.8	6.4
34	2	8.6	3.8	13.4	14.4	9.8	19.0	-5.8	-12.5	0.8
35	1	57.2	48.2	66.2	44.5	37.7	51.4	12.7	1.3	24.0
35	2	63.2	55.1	71.4	62.5	56.4	68.7	0.7	-9.5	10.9
37	1	76.6	69.1	84.1	72.9	66.7	79.1	3.7	-6.0	13.4
37	2	81.6	74.9	88.3	78.9	73.5	84.2	2.7	-5.8	11.3
38	1	59.0	49.9	68.0	54.7	47.8	61.7	4.2	-7.2	15.6
38	2	56.1	47.8	64.4	51.2	44.7	57.6	4.9	-5.6	15.4
39	1	0.0	0.0	0.0	5.4	-0.9	11.8	-5.4	-11.8	0.9
39	2	5.8	-3.1	14.7	9.1	1.3	16.9	-3.3	-15.1	8.5
40	1	0.0	0.0	0.0	1.4	-0.2	3.0	-1.4	-3.0	0.2
40	2	1.1	-0.4	2.6	1.8	0.2	3.4	-0.7	-2.9	1.4
40	1	17.6	10.9	24.4	10.6	6.5	14.7	7.0	-0.9	14.9
40	2	8.0	3.9	12.1	8.5	4.9	12.2	-0.6	-6.0	4.9
42	1	52.6	44.3	61.0	43.8	36.9	50.6	8.9	-2.0	19.7
42	2	47.5	39.4	55.6	41.8	35.6	48.1	5.7	-4.6	15.9
43	1	35.6	27.4	43.8	25.6	19.8	31.5	10.0	-0.1	20.1
43	2	35.5	28.0	43.0	26.9	21.5	32.4	8.6	-0.7	17.8
44	1	10.1	4.2	16.0	12.2	7.3	17.1	-2.1	-9.8	5.6
44	2	20.6	14.1	27.0	21.6	16.3	26.8	-1.0	-9.3	7.3
45	1	9.0	-4.7	22.7	7.8	0.4	15.2	1.2	-14.3	16.8
45	2	6.6	-9.9	23.2	5.2	0.3	10.1	1.4	-15.8	18.7
46	1	63.4	54.6	72.2	56.6	49.9	63.2	6.9	-4.2	17.9
46	2	68.9	61.1	76.7	61.7	55.5	67.9	7.2	-2.8	17.2
47	1	33.0	24.7	41.4	35.9	29.3	42.5	-2.8	-13.5	7.8
47	2	70.4	62.8	77.9	66.6	60.6	72.7	3.7	-6.0	13.4
48	1	68.8	60.6	77.0	64.9	58.5	71.4	3.9	-6.5	14.3
48	2	76.2	69.3	83.0	70.7	64.9	76.5	5.4	-3.6	14.4
49	1	66.5	57.9	75.1	64.5	57.1	71.8	2.1	-9.2	13.3
49	2	68.6	59.9	77.3	67.3	60.6	73.9	1.3	-9.7	12.3

For peer review only

50	1	51.7	42.1	61.4	48.4	40.3	56.4	3.4	-9.2	15.9
50	2	55.2	46.3	64.1	56.1	49.0	63.2	-0.9	-12.3	10.4
51	1	33.2	14.6	51.7	78.0	65.9	90.2	-44.9	-67.0	-22.7
51	2	40.6	17.5	63.7	63.7	48.8	78.5	-23.0	-50.5	4.5
52	1	73.0	65.1	80.9	69.2	62.7	75.6	3.8	-6.3	14.0
52	2	83.8	78.2	89.3	82.1	77.1	87.0	1.7	-5.7	9.1

a. For text see Table S1
b. 1 Aurangabad, 2 Gopalganj
c. Estimated with Stata command svy

For peer review only

Table S5b: Point estimates of weighted indicators and differences between a sample of mothers of 0-2 month old infants and a subsample of mothers of 0-2 month old infants from a sample of mothers of 0-5 month old infants in two districts of Bihar, India

	Indicator	District[a]	Point estimates		Difference		
					Estimate	Confidence interval[b]	
			Subsample	Sample		Lower	Upper
15	Proportion of mothers (home + institutional delivery) of infants (0-2/0-5) months who planned transportation to health facility in their last pregnancy	2	56.8	45.7	11.1	0.9	21.2
17	Proportion of mothers (home + institutional delivery) of infants (0-2/0-5) months who have identified persons who would take care of the baby immediately after birth	1	59.6	48.5	11.1	0.5	21.7
		2	64.4	51.8	12.7	2.6	22.7
23	Proportion of mothers who planned for institutional delivery of infants (0-2/0-5) months identified person to accompany her during the delivery	1	47.5	62.5	-15.0	-26.4	-3.6
24	Proportion of mothers of infants (0-2/0-5) months who were visited by ASHA at least once during their last pregnancy	1	75.1	62.2	12.9	2.7	23.2
26	Proportion of mothers of infants (0-2/0-5) months who were visited by FLWs at least once during their last pregnancy	1	76.2	63.5	12.7	2.6	22.8
31	Proportion of mothers of infants (0-2/0-5) months who were visited home by ASHA within 24 hours of last delivery	1	42.0	29.9	12.1	1.1	23.2
33	Proportion of mothers of infants (0-2/0-5) months who were visited home by any FLW within 24 hours of last delivery	1	43.6	32.2	11.3	0.2	22.5
35	Proportion of mothers of infants (0-2/0-5) months who were visited home by any FLW within first week of last delivery	1	57.2	44.5	12.7	1.3	24.0
51	Proportion of infants aged (0-2/0-5) months who were delivered at home continued with dry cord care	1	33.2	78.0	-44.9	-67.0	-22.7

a. 1 = Aurangabad; 2 = Gopalganj
b. Calculated from standard errors for point estimates estimated with Stata command svy

For peer review only

Table S6: Point estimates and confidence intervals of indicators for a sample of 3-5 month infants and subsample of 3-5 month infants in two districts: Bihar, India, 2015

Indicator[a]	District[b]	3-5 month subsample			3-5 month sample			Difference		
		Point estimate	Confidence interval[c]		Point estimate	Confidence interval[c]		Point estimate	Confidence interval	
			Lower	Upper		Lower	Upper		Lower	Upper
37	1	72.3	62.7	81.8	70.4	64.2	76.5	1.9	13.3	-9.5
37	2	91.3	85.8	96.8	86.2	81.8	90.6	5.1	12.2	-2.0
38	1	49.7	39.3	60.2	45.8	38.9	52.6	4.0	16.4	-8.5
38	2	69.7	61.2	78.2	64.4	58.3	70.5	5.4	15.8	-5.1
52	1	39.7	28.6	50.7	65.3	58.8	71.8	-25.6	-12.8	-38.4
52	2	45.8	35.6	55.9	55.2	49.1	61.2	-9.4	2.4	-21.2

a. For text see Table S1
b. 1 Aurangabad, 2 Gopalganj
c. Estimated with Stata command svy

For peer review only

BMJ Open

How well do mothers recall their own and their infants' perinatal events? A two-district study using cross-sectional stratified random sampling in Bihar, India

Journal:	*BMJ Open*
Manuscript ID	bmjopen-2019-031289.R3
Article Type:	Research
Date Submitted by the Author:	18-Nov-2019
Complete List of Authors:	Valadez, Joseph; Liverpool School of Tropical Medicine, International Public Health<br>Devkota, Baburam; Liverpool School of Tropical Medicine, International Public Health<br>Jeffery, Caroline; Liverpool School of Tropical Medicine<br>Hadden, Wilbur; University of Maryland at College Park, Sociology
**Primary Subject Heading**:	Global health
Secondary Subject Heading:	Epidemiology, Global health, Public health, Research methods
Keywords:	Public health < INFECTIOUS DISEASES, maternal recall, Bihar, India, perinatal, surveys, LQAS

SCHOLARONE™
Manuscripts

How well do mothers recall their own and their infants' perinatal events? a two-district study using cross-sectional stratified random sampling in Bihar, India

Joseph J Valadez[1*], Baburam Devkota[1], Caroline Jeffery[1], Wilbur C. Hadden[2]

[1] Department of International Public Health, Liverpool School of Tropical Medicine, Liverpool, United Kingdom

[2] Department of Sociology, Center for Innovation, University of Maryland, USA

* Corresponding author: Professor Joseph J Valadez, Department of International Public Health, Liverpool School of Tropical Medicine, Pembroke Place, Liverpool, L3 5QA United Kingdom. Email: joseph.valadez@lstmed.ac.uk; Mobile: +44(0)754 8523717

Manuscript word length: 2255

For peer review only

Abstract

Objective: Global monitoring of maternal, newborn and child health (MNCH) programmes use self-reported data subject to recall error which may lead to incorrect decisions for improving health services and wasted resources. To minimize this risk, samples of mothers of infants aged 0-2 and 3-5 months are sometimes used. We test whether a single sample of mothers of infants 0-5 months provides the same information.

Design: An annual MNCH household survey in 2-districts of Bihar, India (N=6million).

Participants: Independent samples (n=475 each) of mothers of infants 0-5, 0-2 and 3-5 months.

Outcome measures: Main analyses compare responses from the 0-5 and 0-2 month samples with Mantel-Haenszel-Cochran statistics using 51 indicators in 2-districts.

Results: No measurable differences detected in 79.4% (81/102) comparisons; 20.6% (21/102) display differences for the main comparison. Sub-analyses produce similar results. A difference detected for exclusive breastfeeding is due to premature complementary-feeding by older infants. Measurable differences were detected in 33% (8/24) of the indicators on Front Line Worker (FLW) support, 26.9% (7/26) of indicators of birth preparedness and place of birth and

For peer review only

attendant, and 9.5% (4/42) of the indicators on neonatal and antenatal care.

Conclusions: Differences in FLW visits and compliance with their advice may be due to seasonal effects: mothers of older infants 3-5 months were pregnant during the dry season; mothers of infants 0-2 months were pregnant during the monsoons, making transportation difficult. Useful coverage estimates can be obtained by sampling mothers with infants 0-5 months as with two samples suggesting that mothers of young infants recall their own perinatal events and those of their children. For some indicators (e.g., exclusive breast feeding) it may be necessary to adjust targets. Excessive stratification wastes resources, does not improve the quality of information, and increases the burden placed on data collectors and communities, which can increase non-sampling error.

STRENGTHS AND LIMITATIONS OF THIS STUDY

- Strength: The data were produced using stratified random sampling with no apparent design effect leading to an efficient use of information.
- Strength: Data were collected from female participants by female data collectors which is likely to have reduced non-sampling error.

For peer review only

- Strength: The large study population covers a large geographical area, reducing the likelihood that the results are pertinent only to a small group of mothers with infants, and may be generalizable.
- Strength: Both weighted and unweighted results are presented giving strength to the conclusions.
- Limitation: Due to insufficient overlap of variables in the 0-5 month sample and the 3-5 month sample, comparison between the 3-5 month sample vis a vis the 0-5 month sample was not possible.

Key Words:

Maternal recall, surveys, maternal health, MNCH, LQAS, Bihar, India

INTRODUCTION

The progress toward United Nations' Sustainable Development Goal (SDG) 3 is measured with 9 targets, including the Maternal Mortality Ratio (MMR) and the under 5 mortality rate (U5MR) (1, 2). In India, Bihar is one of the largest (population 110 million) and poorest (53% of households are in the lowest wealth index quintile of India (3)) states with high child and maternal mortality (U5MR=54, MMR=208)(4), and is a priority for donor support for health systems strengthening (see (5) for an evaluation of the health care system in Bihar).

For peer review only - http://bmjopen.bmj.com/site/about/guidelines.xhtml

To accelerate progress toward achieving SDG 3, state governments in India pursue programmes of community-based care (see (6, 7) for descriptions and assessments of this approach). Since 2011, the Bihar Ministry of Health has supported an Integrated Family Health Initiative to improve the availability, quality and use of prenatal, perinatal and postnatal care for mothers and infants (8).

The usual way to monitor progress toward achieving these goals is with household surveys. Perhaps the most commonly used surveys are cluster sample surveys such as the Demographic and Health Surveys and the Multiple Indicator Cluster Surveys (9, 10). An alternative design is Lot Quality Assurance Sampling (LQAS), which provides comparable data but is decentralized to local health services organisations and more useful for management and programme planning(11). Several states in India find it benefits their programs (12). Surveys rely on the reports of mothers of infants and young children, but these reports are subject to several sources of potential error and bias through interviewees not knowing, forgetting, and having memory errors (13, 14). Studies have shown both that mothers can accurately report significant facts about the birth and care of their children many years after the event (15), but also that even immediately after giving birth

For peer review only - http://bmjopen.bmj.com/site/about/guidelines.xhtml

mothers may misreport details (16-18). Studies of mothers recall of their children's vaccination status concluded that due to offsetting errors of maternal reports, the resulting data accurately measured vaccination rates (19); the pattern of error revealed that mothers whose children are up-to-date or nearly so tended to underestimate their child's vaccination status whilst mothers whose children have few vaccinations, overestimate their coverage.

To improve the validity of collected data, knowledge, practice and coverage surveys have used samples of mothers of infants 0-11 months of age or 0-5 months of age and children 6-11 months of age. In Bihar, local organisations departed from this convention of sampling among these three cohorts of children under one year of age and have been monitoring their programs' progress by sampling five dedicated cohorts: mothers of children 0-2, 3-5, 6-8, 9-11 and 12-23 months old with indicators focused on antenatal care, safe delivery practices, infant and young child feeding practices, immunisation, treatment seeking, and more. To avoid the possibility of maternal recall error, each of the five cohorts was asked questions particularly relevant to a child's specific age group.

In countries such as India with high maternal and child mortality rates, regular monitoring of related health

For peer review only - http://bmjopen.bmj.com/site/about/guidelines.xhtml

service coverage is critical to reducing these rates. However, survey designs should be affordable and sustainable for local health systems; they should also produce precise, unbiased estimates (20). In this study, we explore whether information is gained by sampling cohorts of children aged 0-2 and 3-5 months or whether sample sizes can be reduced by 50% by creating one sample cohort aged 0-5 months.

The research question we address is: “Do the health service delivery coverage estimates from a sample of mothers of infants aged 0-5 months differ from those obtained from a sample of mothers of infants ages 0-2 months?” A corollary to this question is: “Do mothers of infants 3-5 months of age display more recall error relative to mothers of infants 0-2 months of age for antenatal, delivery or young infant health practices?” We compare district coverage estimates obtained from two independent samples of infants 0-2 months and 0-5 months of age. The implications of this study are important for health systems researchers needing results to appraise and improve their programmes.

METHODS

To answer this question, we collected information from a sample of mothers with infants 0-5 months old and a sample

For peer review only

of mothers with infants 0-2 month old in two districts. This study took place within the context of a larger survey that also sampled children 3-5, 6-8, 9-11 and 12-23 months of age. These four latter samples used questionnaires with variables that either did not overlap at all or overlapped on very few indicators with the questionnaires used to interview the 0-5 month and 0-2 month samples. Due to this constraint, in this study, we only use the two aforementioned groups to assess the measurement of the indicators and refer only to them for the remainder of this paper. The household sampling design we used is a stratified random sample (21). Within each district, the strata are administrative units of the health system, which in Bihar is called a *block*. Within each block the primary sampling unit is the Anganwadi Centre (Community Health Subcentre) Catchment Area (ACCA); 19 ACCA are selected from each block with probability proportional to size. From each ACCA one respondent is randomly selected from each age-group under study using segmentation sampling (22, 23). The sample size 19 for each block is chosen to maximize the probability of correctly classifying a block with reference to performance targets on health related indicators (95% reliability) while balancing the probability (10% margin of error) of incorrectly classifying a block and thereby failing to recognize either the accomplishments of local health care delivery systems or

the local population's health care needs (22). For this purpose, principles of Lot Quality Assurance Sampling were used along with established probability tables (24-26).

There are 14 and 11 blocks in Gopalganj and Aurangabad (N=6 million), the two districts selected for this study, respectively. The total sample sizes are: (a) Gopalganj: 19 x 14 blocks= 266 infants 0-2 months and 266 infants 0-5 months, and (b) Aurangabad: 19 x 11 blocks= 209 infants 0-2 months and 209 infants 0-5 months. The 0-5 month old sample is 60% 0-2 months old and 40% 3-5 months old.

Using summary data from each of the two samples we analyse the data with Cochran-Mantel-Haenszel (CMH) (27) tests for 51 dichotomous indicators (See Supplementary File Table S1) common to the two samples. The CMH tests theoretically have a Chi-square probability distribution with 1 degree of freedom. With a sufficient number of respondents or a sufficient number of blocks the CMH test is equivalent to a conditional logistic regression (28: 114-115). In this analysis both the number of respondents and the number of blocks only approach sufficiency. Consequently, the calculated chi-squares and probabilities must be considered as approximations of their true values.

We calculate both unweighted and weighted estimates. The unweighted estimates permit the results from smaller blocks to have equal weight vis à vis larger ones. Since the

For peer review only - http://bmjopen.bmj.com/site/about/guidelines.xhtml

research question concerns an analysis of which age cohort is most informative, the weighted estimates may not be as useful as the unweighted ones. However, the weighted estimates provide better point estimates of the indicators at the district level. The effect of the weights on the Chi-square statistics is to increase the contribution of the larger blocks and decrease the contribution of the smaller blocks. Hence, we report both sets of results (See Supplementary File Tables S2-S3).

The Chi-square probability distribution puts the differences between the districts on a probability scale (See Supplementary File Table S2). To determine meaningful differences in responses between the two age cohorts we used a probability of 0.05 as a cut-off value and consider differences with probabilities less than 0.05 to be possibly meaningful and those with larger probabilities to be likely due to sampling errors. With 102 comparisons (51 indicators weighted or unweighted) we must expect some to exceed this cut-off by chance alone. If all of the comparisons were independent, we might randomly find 5 differences, but many of the indicators measure related events (e.g., number of ANC visits and tetanus toxoid vaccinations) and the weighted and unweighted estimates are similar, so these indicators are not all independent and it

For peer review only

is not possible to calculate an expected number of differences nor is it appropriate to interpret these probabilities as measures of “statistical significance”.

Patient and Public Involvement

This study does not involve patients. Also, the public was not involved in the design, conduct and reporting of the research. The public was engaged as interviewees. To ensure local engagement we coordinated with the Bihar Ministry of Health, local implementing non-governmental organizations and our donor. We also shared the results with them and offered further dissemination of results.

RESULTS

We find a high level of agreement between the two samples (Table 1). Out of 102 weighted and unweighted comparisons between the estimates from the 0-2 month and 0-5 month samples there is no probable difference in 81 (79.4%) in both the unweighted and weighted estimates. We detect that probable differences exist for 13 comparisons (12.7%). For the remaining eight comparisons the weighted and unweighted estimates disagree. The weighted estimates find seven differences that the unweighted estimates do not; the unweighted estimates find one difference that the weighted estimates do not find.

For peer review only - http://bmjopen.bmj.com/site/about/guidelines.xhtml

Table 1. Number of indicators by probability of a difference between the 0-2 months and the 0-5 months samples for weighted and unweighted samples

Unweighted	Weighted					
	Aurangabad		Gopalganj		Total	
	>= .05	< .05	>= .05	< .05	>= .05	< .05
>= .05	40	3	41	4	81	7
< .05	0	8	1	5	1	13

For different health service domains, the number of indicator comparisons varies from two (Exclusive Breastfeeding-EBF) to 24 concerning home visits by Front Line Worker (FLW) support (Table 2). The two principal FLW are Anganwadi workers and Accredited Social Health Activists (ASHA).

For peer review only

For peer review only - http://bmjopen.bmj.com/site/about/guidelines.xhtml

Table 2. Number of indicator comparisons by subject domain showing a measurable difference using weighted and unweighted estimates of 0-2 months and 0-5 months samples

Health Service Domain	Total comparisons	No measurable difference between 0-2 and 0-5 months results	Measurable difference between 0-2 and 0-5 months results			
			Both	Unweighted only	Weighted only	Percent (%) indicators with different results
Antenatal care	22	21	0	0	1	5
Place of birth & attendant	8	6	1	0	1	25
Birth preparedness	18	13	3	0	2	28
Front Line Worker support	24	16	6	0	2	33
Maternal health	8	8	0	0	0	0
Neonatal care	20	17	1	1	1	15
Exclusive Breastfeeding	2	0	2	0	0	100
Totals	102	81	13	1	7	21.9

For peer review only - http://bmjopen.bmj.com/site/about/guidelines.xhtml

In the FLW support domain 33% of comparisons have probable differences. The neonatal health domain has 20 comparisons and the birth preparedness domain has 18; in these domains 15% and 28% show probable differences, respectively. Place of birth and attendant, and maternal health each have eight comparisons with 25%, or two comparisons, and 0 comparisons, respectively, showing a possible difference. The differences between the two samples cluster around home visits from FLW and behaviours associated with birth preparedness and neonatal care. Details of these differences are listed in Table 3.

For two indicators, both the weighted and unweighted estimates display probable differences between the 0-2 and 0-5 months samples in both districts. For indicator #52, the proportion exclusively breastfeeding, the 0-5 months cohort has the lower estimate, and indicator #24, the proportion of mothers visited by an ASHA at least once during their last pregnancy, the 0-5 months sample gives the higher estimate, about 74%, compared to 63% in the 0-2 months sample (See Supplementary Tables S2-3).

Additional analyses comparing subsamples of mothers of infants 0-2 months and 3-5 months from the 0-5 months

For peer review only - http://bmjopen.bmj.com/site/about/guidelines.xhtml

sample, the sample of mothers of infants 0-2 months and the subsample of infants 0-2 month, and the sample of mothers of infants 3-5 months and the subsample of infants 3-5 months produced similar results (See Supplementary Text and Tables S4a-b, Tables S5a-b and Table S6).

For peer review only

Table 3. Indicators by health service domain showing measurement differences between 0-2 months and 0-5 months samples							
Health Service Domain and Indicator	Indicator No.	District	Weighted Coverage (%)		p-value		Estimate type
			0-2 months	0-5 months	Unweighted Estimate	Weighted Estimate	
**Antenatal care**							
Proportion of mothers of infants (0-2/0-5 months) registered during their last pregnancy	1	Aurangabad	85.2	77.6	0.0552	0.0365	Weighted
**Place of birth & attendant**							
Proportion of mothers of infants (0-2/0-5 months) whose last child was delivered at a public facility	38	Gopalganj	51.2	61.6	0.0363	0.0159	Both
Proportion of mothers of infants (0-2/0-5 months) whose last child was delivered at a health facility (private or public facility)	37	Gopalganj	78.9	85.5	0.0643	0.0459	Weighted
**Birth preparedness**							
Proportion of mothers of infants (0-2/0-5 months) who planned transportation to health facility in their last pregnancy (home & institutional delivery)	15	Gopalganj	45.7	56.0	0.0266	0.0158	Both
Proportion of mothers of infants (0-2/0-5 months) who identified persons to care for the baby immediately after birth (home + institutional delivery)	17	Gopalganj	51.8	62.6	0.0255	0.0103	Both
Proportion of mothers of infants (0-2/0-5 months) who planned for institutional delivery and	23	Aurangabad	62.5	47.0	0.0039	0.0052	Both

For peer review only - http://bmjopen.bmj.com/site/about/guidelines.xhtml

identified person to accompany her during the delivery							
Proportion of mothers who planned for institutional delivery of infants (0-2/0-5 months) who had a new blade & thread for their delivery	19	Aurangabad	23.5	14.5	0.062	0.0429	Weighted
Proportion of mothers who planned institutional delivery of infants (0-2/0-5 months) who arranged clean cloth for mothers and baby	21	Aurangabad	43.6	31.2	0.0546	0.0137	Weighted
**Front Line Worker support**							
Proportion of mothers of infants (0-2/0-5 months) who were visited by ASHA at least once during their last pregnancy	24	Aurangabad	62.2	75.2	0.0023	0.0042	Both
		Gopalganj	63.5	73.0	0.0284	0.0175	Both
Proportion of mothers of infants (0-2/0-5 months) visited at home by FLWs at least once during their last pregnancy	26	Aurangabad	63.5	76.7	0.0021	0.0032	Both
Proportion of mothers of infants (0-2/0-5 months) visited at home by ASHA within 24 hours of last delivery	31	Aurangabad	29.9	44.9	0.0009	0.0016	Both
Proportion of mothers of infants (0-2/0-5 months) visited at home by any FLW within 24 hours of last delivery	33	Aurangabad	32.2	46.7	0.0015	0.0026	Both
Proportion of mothers of infants (0-2/0-5 months) visited at home by any FLW within first week of last delivery	35	Aurangabad	44.5	59.3	0.0018	0.0026	Both
Proportion of mothers of infants (0-2/0-5 months) visited at home by any AWW within the first week of the last delivery	34	Gopalganj	14.4	8.9	0.0959	0.0471	Weighted
Proportion of mothers of infants (0-2/0-5 months) visited by	27	Gopalganj	52.6	61.1	0.0617	0.0449	Weighted

For peer review only - http://bmjopen.bmj.com/site/about/guidelines.xhtml

For peer review only

ASHAs at least once during their last trimester of pregnancy							
**Infant care**							
Proportion of infants aged (0-2/0-5 months) who were delivered at home continued with dry cord care	51	Aurangabad	78.0	45.4	0.0001	0.0006	Both
		Gopalganj	63.7	41.1	0.0431	0.0627	Unweighted
Proportion of infants aged (0-2/0-5 months) weighed after birth (Public facility/Private facility/Home)	48	Gopalganj	70.7	78.2	0.0727	0.0464	Weighted
**Exclusive Breastfeeding**							
Proportion of infants (0-2/0-5 months) breast-fed in the past 24 hours (Exclusively Breast-Fed)	52	Aurangabad	69.2	59.7	0.0229	0.0411	Both
		Gopalganj	82.1	68.4	0.0001	0.0003	Both

For peer review only

DISCUSSION

Statement of principal findings

There are no measurable differences in coverage estimates for 79.4% (81 comparisons) of the indicator comparisons between the samples of mothers with infants 0-2 months old versus mothers of infants 0-5 months old; 12.7% (13 comparisons) display measurable differences. The remaining 7.8% (eight comparisons) display discrepancies between the weighted and unweighted estimates.

Strengths and weaknesses of the study

The strengths of this study are that it compares estimates from two independent samples and that there are many estimates from diverse domains. The weaknesses of this study are that the data were collected in only 2 districts of 1 state in India and in different months of a single year, and that indicators from the sample of mothers of 3-5 month old infants comparable to those of the 0-2 month old infants, using the same questionnaire, were not collected. Supplemental analyses comparing 0-2 and 3-5 month subsamples of the 0-5 sample did not uncover evidence of bias due to the combination of these two age groups.

For peer review only - http://bmjopen.bmj.com/site/about/guidelines.xhtml

Strengths and weaknesses in relation to other studies

Other studies of maternal recall bias have sought a "gold standard" to represent reality and to evaluate measures. Our study, of course, is interested in reality, but this study compares alternative measures needed to assess the Bihar health program. It also uses a complete sample of the age grouping under study rather than just a sub-sample of a larger age grouping. A weakness of this approach is that the analysis does not result in a formal statistical test; our conclusion is based on the weight of the evidence.

Meaning of the study

The evidence indicates that samples of the broader group yield comparable results to those of the narrower age group. It is not necessary to double the total sample by measuring independently 0-2 months and 3-5 months cohorts of children. These results also tend to dispel the hypothesis that maternal recall is problematic for mothers during the first 6 months following delivery. Our results are more consistent with conclusions presented in earlier research (15) and they support those organizations collecting data with 0-5 month cohorts.

For peer review only - http://bmjopen.bmj.com/site/about/guidelines.xhtml

Indicator #52, EBF, displayed two comparisons measuring decreases in both districts. This is not surprising as fewer infants are expected to be exclusively breast fed in a sample ranging from 0 to 5 months than a sample ranging 0 to 2 months; mothers introduce complementary feeding and liquids as infants age despite this being a health risk. This difference could be accommodated by adjusting expectations and targets for the indicator.

Unanswered questions and future research

Further investigation and consideration of the differences is warranted. The eight differences found in the FLW support indicators deserve more scrutiny. Seven show higher estimates for the 0-5 cohort and one has a higher estimate for the 0-2 month cohort. The former seven differences may be due to excessive rainfall during the July-September (monthly average 318.95mm, range:195.99-395.8mm) versus the lesser rainfall during June-April (monthly average 25.52mm, range: 0.3-27.88mm) which in the last trimester may have reduced the access of ASHA in the 0-2 month cohort (29). Indicators such as these may be particularly sensitive to rainfall and may explain why more mothers in the 0-5 month cohort displayed higher FLW visitation estimates since FLW

For peer review only - http://bmjopen.bmj.com/site/about/guidelines.xhtml

were not impeded by the monsoon and the resulting muddy roads.

Differences in birth preparedness and institutional birth may be a consequence of differences in rainfall or in FLW support; the results signal a need for more careful planning when transportation is difficult and decreases the effectiveness of FLW by reducing their access to women. Or, some of these differences may just be due to noise in the data.

CONCLUSIONS

Overall, the answer to the research question, "Can one get the same district coverage estimates from a sample of mothers of infants aged 0-5 months as from a sample of mothers of infants ages 0-2 months?" is yes. This result can be paraphrased as: mothers do not display increased recall errors of their perinatal health care behaviour in a cohort of mothers with infants 0-5 months as compared with mothers with younger infants. Substantial resources and effort can be saved using a survey design that avoids needless expenses to collect data that provides insubstantial amounts of information. It also reduces the burden on data collectors and community participants.

For peer review only - http://bmjopen.bmj.com/site/about/guidelines.xhtml

Fatigue to both groups can result in needless non-sampling error.

For peer review only

For peer review only - http://bmjopen.bmj.com/site/about/guidelines.xhtml

ACKNOWLEDGMENTS

We gratefully acknowledge the essential roles of Hemant Das, Alok Prahdan and Sanjay Biswa for their careful field work supporting the LQAS survey and the HMIS data retrieval. We thank Prof Imelda Bates, Prof Brian Faragher and Nancy Vollmer for their valuable feedback on an earlier version of this manuscript. This work was supported by the Bill and Melinda Gates Foundation (Investment ID OPP1142889).

For peer review only

ETHICS

The Ethical Committees of the Indian Institute of Public Health (No IIPHB-IEC-2016/010) and the Liverpool School of Tropical Medicine Research Ethics Committee approved the protocol, study instruments and consent procedures for the data collection of the household surveys (Research Protocol 16-023).

COMPETING INTERESTS

The authors have read the statement on competing interests and declare that they have none.

AUTHORS' CONTRIBUTIONS

JJV, BD developed the research question and survey design; WH, CJ carried out the statistical analyses; JJV obtained the funding and donor support for the research; BD trained and managed the survey teams in Bihar; JJV, WH, CJ interpreted the data; CJ responsible for data curation; all authors wrote and reviewed the paper.

FUNDING

This research was funded by the Bill and Melinda Gates Foundation Investment ID OPP1142889

DATA SHARING STATEMENT

Data are available upon reasonable request.

REFERENCES

1. UNSTATS. Official list of MDG indicators: United Nations Statistics Division, Department of Economic and Social Affairs; 2005 [Available from: https://unstats.un.org/unsd/mdg/Host.aspx?Content=Indicators/OfficialList.htm.
2. UN DESA. Sustainable Development Goals: Division for Sustainable Development Goals, United Nations Department of Economic and Social Affairs; 2015 [Available from: https://www.un.org/sustainabledevelopment/sustainable-development-goals/.
3. International Institute for Population Sciences (IIPS) and ICF. National Family Health Survey (NFHS-4), 2015-16: India. Mumbai: IIPS; 2017.
4. MOSPI. Millennium Development Goals India Country Report 2015. New Delhi: Social Statistics Division, Central Statistics Office, Ministry of Statistics and Programme Implementation; 2015.
5. Karvande S, Sonawane D, Chavan S, Mistry N. What does quality of care mean for maternal health providers from two vulnerable states of India? Case study of Bihar and Jharkhand. Journal of Health, Population and Nutrition. 2016;35(6):1-10.
6. Mohan P, Kishore B, Singh S, Bahl R, Puri A, Kumar R. Assessment of implementation of integrated management of neonatal and childhood illness in India. Journal of Health, Population and Nutrition. 2011;29(6):639-38.
7. Neogi SB, Sharma J, Chauhan M, Khanna R, Chokshi M, Srivastava, et al. Care of newborn in the community and at home. Journal of Perinatology. 2016;36(Suppl 3):s13-7.
8. CARE Family Health. Integrated Family Health Initiative: Catalysing change for healthy communities. 2013.
9. Blanc AK, Warren CE, McCarthy KJ, Kimani J, Ndwiga C, RamaRao S. Assessing the validity of indicators of the quality of maternal and newborn health care in Kenya. Journal of Global Health. 2016;6(1):1-11.
10. Eisele TP, Rhoda DA, Cutts FT, Keating J, Ren R, Barros AJD, et al. Measuring coverage in MNCH: Total survey error and the interpretation of intervention coverage estimates from household surveys. PLoS Medicine. 2013;10(5):e1001386.
11. Anoke SC, Mwai P, Jeffery C, Valadez JJ, Pagano M. Comparing two survey methods of measuring health-related indicators: Lot Quality Assurance Sampling and Demographic Health Surveys. Tropical Medicine and International Health. 2015;20(12):1756-70.

For peer review only

12. Valadez JJ, Devkota B, Pradhan MM, Meherda P, Sonal GS, Dhariwal A, et al. Improving malaria treatment and prevention in India by aiding district managers to manage their programmes with local information: a trial assessing the impact of Lot Quality Assurance Sampling on programme outcomes. Trop Med Int Health. 2014.
13. Tourangeau R. Remembering what happened: Memory errors and survey reports. In: Stone AA, Jobe JB, Bachrach CA, Cain VS, Kurtzman HS, editors. The Science of Self-report : Implications for research and practice. Mahwah, NJ: Lawrence Erlbaum; 2000.
14. Bradburn NM. Temporal representation and event dating. In: Stone AA, Jobe JB, Bachrach CA, Cain VS, Kurtzman HS, editors. The Science of Self-report : Implications for research and practice. Mahwah, NJ: Lawrence Erlbaum; 2000.
15. Tomeo CA, Rich-Edwards JW, Michels KB, Berkey CS, Hunter DJ, Frazier AL, et al. Reproducibility and validity of maternal recall of pregnancy-related events. Epidemiology. 1999;10(6):774-7.
16. Elkadry E, Kenton K, White P, Creech S, Brubaker L. Do mothers remember key events during labor? American Journal of Obstetrics and Gynecology. 2003;189(1):195-200.
17. Li R, Scanlon KS, Serdula MK. The validity and reliability of maternal recall of breastfeeding practice. Nutrition Reviews. 2005;63(4):103-10.
18. Miles M, Ryman TK, Dietz V, Zell E, Luman ET. Validity of vaccination cards and parental recall to estimate vaccination coverage: A systematic review of the literature. Vaccine. 2013;31:1560-8.
19. Valadez JJ, Weld LH. Maternal recall error of child vaccination status in a developing nation. Am J Public Health. 1992;82(1):120-2.
20. Chan M, Kazatchkine M, Lob-Levyt J, Obaid T, Schweizer J, Sidibe M, et al. Meeting the demand for results and accountability: a call for action on health data from eight global health agencies. PLoS Med. 2010;7(1):e1000223.
21. Valadez JJ, Weiss W, Leburg C, Davis R. Assessing Community Health Programs: A Trainer's Guide. Using LQAS for Baseline Surveys and Regular Monitoring. 2nd ed. St Albans: Teaching-aids At Low Cost; 2007.
22. Turner AG, Magnani RJ, Shuaib M. A not quite as quick but much cleaner alternative to the Expanded Programme on Immunization (EPI) Cluster Survey design. International Journal of Epidemiology. 1996;25(4):198-203.
23. Davis RH, Valadez JJ. Improving the collection of knowledge, attitude and practice data with community surveys: a comparison of two second-stage sampling methods. Health Policy Planning. 2014;29(8):1054-60.
24. Dodge HF, Romig HG. Sampling inspection tables: single and double sampling. 2nd ed. New York: John Wiley & Sons; 1959.
25. Robertson SE, Joseph J V. Global review of health care surveys using lot quality assurance sampling (LQAS), 1984–2004. Social Science & Medicine. 2006;63:1648-60.
26. Valadez JJ. Assessing child survival programmes in developing countries: testing lot quality assurance sampling. Boston: Harvard University Press; 1991.
27. Mantel N. Chi-square tests with one degree of freedom: extensions of the Mantel-Haenszel procedure. J Am Stat Assoc. 1963;58(303):690-700.
28. Agresti A. An Introduction to Categorical Data Analysis. 2nd ed. Hoboken, New Jersey: Wiley-Interscience; John Wiley & Sons; 2007.

For peer review only

29. World Weather Online. Patna Monthly Climate Averages, Bihar, India 2018. Available from: https://www.worldweatheronline.com/patna-weather-averages/bihar/in.aspx.

For peer review only

Supplementary Text for Tables S4a through S6

We make four comparisons to inform an evaluation of these data. The first compares the 0-5 month sample to the 0-2 month sample and is presented in Table 2 in the main text and Tables S2-3. In a second comparison, indicators estimated for the 0-2 month old **subsample** are compared to the 3-5 month old **subsample** to assess the internal consistency of estimates for the entire 0-5 sample (Table 4a-b). In the third comparison, the 0-2 **subsample** of the 0-5 sample is compared to the 0-2 month **sample** to assess the sampling variability for this age group (Tables S5a-b). Finally, in the fourth comparison, the 3-5 **subsample** of the 0-5 sample is compared to the 3-5 month **sample** (Table S6). This last comparison is limited because the 3-5 month sample collected limited data; there are only 3 indicators common to the two samples.

Table S4a has the complete results for the second comparison with point estimates for the two **subsamples** 0-2 and 3-5 of the 0-5 sample, differences and estimated confidence intervals for the estimates and differences of 104 comparisons. Fourteen indicators for which the estimated confidence interval of the difference does not include zero are listed in Table S4b. The table has 2 panels. In the top panel are 6 indicators for which a difference is also reported in Table S2. Table S2 compares the 0-2 month sample and the 0-5 month sample. For each of these indicators the reported difference is in the same direction. Two of these indicators are indicator 52, exclusive infant breast feeding, in the 2 provinces. These differences are not surprising as it is common for mothers to introduce supplemental foods as infants age. The authors speculate in their paper that the timing of the monsoon may have reduced some of the indicators for the younger infants. Of the remaining four indicators of the top panel, three of the indicator differences are negative, lower for the younger infants and might plausibly be related to a monsoon. In the bottom panel of Table S4b are 8 indicators where zero is not in the confidence interval of the difference between subsamples, which suggests difference, but the results between two full-samples in Tables S2 and S3 suggest there is no meaningful difference, except that one of them, indicator 37 in Gopalganj; it does show a difference in Table S2. Of these 8, the difference between samples is in the same direction five times and in a different direction three times. For the 5 indicators where the differences are in the same direction, the absolute value of the differences between 0-2 subsample and the 3-5 subsample are larger than the absolute values of the differences between the 0-2 month sample and the 3-5 subsample. For these 5 indicators the 0-2 month **sample** is more like the 3-5 **subsample** than the 0-2 **subsample**. For the 3 indicators where the differences are in the different directions the 0-2 **subsample** closely resembles the 0-2 month **sample**; two of the estimates are different by less than 1 percent and the third by 2.4 percent.

A third comparison is in Tables S5a-b. In Table S5a the 0-2 **subsample** is compared to the 0-2 month **sample**. The expectation here would be that there are no differences because these two samples are designed to represent the same population. However, this expectation is not met; there are 10 differences where the confidence interval for the difference does not contain 0. These differences are listed in Table S5b. Nine of these 10 indicators are also among the differences in Table S3 and Table 3 in the paper. For 3 of these 9 differences the 3-5 subsample is closer to the 0-2 month sample than it is the 0-2 subsample (indicators 15, 17-2, 51); and for 3 indicators the 3-5 subsample is further from the 0-2 month sample than the 0-2 subsample (indicators 31, 33, 35). For 3 indicators the 0-2 and 3-5 subsample indicator values are nearly equal (indicators 23, 24, 26). For the final indicator in this list (indicator 17-1, the indicator for which zero is in the confidence interval of the difference between the 0-2

For peer review only

month sample and the 0-5 sample in Table S3) the difference between the indicator values for the 0-2 and 3-5 subsamples and the 0-2 month sample are about equal in magnitude but have opposite signs.

Results from the fourth comparison, comparing the 3-5 **subsample** of the 0-5 **sample** to the 3-5 month sample are limited, as noted above. There are only 3 indicators in the 2 districts – six comparisons. Zero is within the confidence interval of 5 of the differences (Table S6).

We note in the paper that in making this number of comparisons one must expect that some will be large enough to be considered meaningful by chance alone. In the above analysis there are 3 comparisons of 51 indicators in 2 provinces producing 16, 14 and 10 differences and a fourth comparison with 1 difference in 6. In their paper the authors find that some of these differences are readily understood and others may be interpreted effects of monsoon rains. Post hoc interpretation is risky here; many of these differences may be noise in the data. There is evidence in the third comparison to support this position with 3 differences moving the 0-5 **sample** closer to the 0-2 month **sample**, 3 moving it away, and 4 not moving it one way or the other. Furthermore, 9 of the 10 differences in the third comparison are also differences in the first comparison, suggesting that about half the differences between the 0-2 month sample and the 0-5 sample may be due to differences between the 0-2 month **sample** and the 0-2 **subsample** of the 0-5 sample.

The second comparison, comparing the 0-2 subsample to the 3-5 subsample, provides evidence both undermining and supporting the conclusion that a 0-5 sample will provide the same answers as two samples 0-2 and 3-5. On the one hand 5 of the fourteen differences in the third comparison are also in the first, the comparison of the 0-5 sample to the 0-2 sample, suggesting that the inclusion of 3-5 month olds in the 0-5 sample might contribute to the differences. On the other hand, in 5 out of the 8 indicators that are not different between the 0-2 and 0-5 samples, the 3-5 subsample more closely matches the 0-2 month sample than does the 0-2 subsample of the 0-5 sample. That this is true in general and not only for these extreme differences is suggested by the mean absolute differences between samples which are 5.2 for the differences between the 0-2 month **sample** and 3-5 month **subsample** and 6.6 for the differences between the 0-2 month **subsample** and the 3-5 month **subsample**.

Finally, the overall result of all these comparisons is the same as that of the comparison presented in the main text of the paper: in each comparison 85% or more of the indicators show no differences between the samples. The most consistent evidence of differencCae is between the 0-2 **subsample** of the 0-5 sample and the 0-2 month **sample**.

For peer review only

Table S1. Subject domain and indicators labels

Domain[a]	Indicator	Indicator text
1	1	Proportion of mothers of infants (0-2/0-5) months who were registered during their last pregnancy
1	2	Proportion of mothers of infants (0-2/0-5) months whose last pregnancy was registered in the first three months of pregnancy
1	3	Proportion of mothers of infants (0-2/0-5) months attended at least one ANC visit during their last pregnancy
1	4	Proportion of mothers of infants (0-2/0-5) months attended 3 or more ANC visits during their last pregnancy
1	5	Proportion of mothers of infants (0-2/0-5) months attended 4 or more ANC visits during their last pregnancy
1	6	Proportion of mothers of infants (0-2/0-5) months who attended at least one ANC visit where BP was checked during her last pregnancy
1	7	Proportion of mothers of infants (0-2/0-5) months who attended at least one ANC where at least one sonography was performed during her last pregnancy
1	8	Proportion of mothers of infants (0-2/0-5) months who attended at least one ANC where at least one abdominal examination was performed during her last pregnancy
1	9	Proportion of mothers of infants (0-2/0-5) months who attended at least one ANC where at least one urine test was performed during her last pregnancy
1	10	Proportion of mothers of infants (0-2/0-5) months who attended at least one ANC where at least one blood test was performed during her last pregnancy
1	40	Proportion of mothers of infants (0-2/0-5) months who have used an ambulance to reach an institution for last delivery
2	11	Proportion of mothers of infants (0-2/0-5) months who were protected against tetanus in their last pregnancy (Neonatal TT)
2	12	Proportion of mothers of infants (0-2/0-5) months who received two or more doses of TT injection in their last pregnancy
2	13	Proportion of mothers of infants (0-2/0-5) months who received IFA for 100 days or more during their pregnancy
2	14	Proportion of mothers of infants (0-2/0-5) months consumed IFA for 100 days or more in their pregnancy
3	15	Proportion of mothers (home + institutional delivery) of infants (0-2/0-5) months who planned transportation to health facility in their last pregnancy
3	16	Proportion of mothers (home + institutional delivery) of infants (0-2/0-5) months who have identified anybody who would donate blood in the case of emergency in their last pregnancy

For peer review only

3	17	Proportion of mothers (home + institutional delivery) of infants (0-2/0-5) months who have identified persons who would take care of the baby immediately after birth
3	18	Proportion of mothers (home + institutional delivery) of infants (0-2/0-5) months who have arranged new blade & thread for their last delivery
3	19	Proportion of mothers who planned for institutional delivery of infants (0-2/0-5) months who arranged new blade & thread for their delivery
3	20	Proportion of mothers (home + institutional delivery) of infants (0-2/0-5) months who have arranged clean cloth for mothers and baby
3	21	Proportion of mothers who planned institutional delivery of infants (0-2/0-5) months who arranged clean cloth for mothers and baby
3	22	Proportion of mothers of infants (0-2/0-5) months who have saved money for the delivery
3	23	Proportion of mothers who planned for institutional delivery of infants (0-2/0-5) months identified person to accompany her during the delivery
4	24	Proportion of mothers of infants (0-2/0-5) months who were visited by ASHA at least once during their last pregnancy
4	25	Proportion of mothers of infants (0-2/0-5) months who were visited by AWW at least once during their last pregnancy
4	26	Proportion of mothers of infants (0-2/0-5) months who were visited by FLWs at least once during their last pregnancy
4	27	Proportion of mothers of infants (0-2/0-5 months) visited by ASHAs at least once during their last trimester of pregnancy
4	28	Proportion of mothers of infants (0-2/0-5) months who were visited by AWW at least once during their last pregnancy
4	29	Proportion of mothers of infants (0-2/0-5) months who were visited by any FLW in the last trimester during their last pregnancy
4	30	Proportion of mothers of infants (0-2/0-5) months who were visited 2 or more times by any FLW in the last trimester during their last pregnancy
4	31	Proportion of mothers of infants (0-2/0-5) months who were visited home by ASHA within 24 hours of last delivery
4	32	Proportion of mothers of infants (0-2/0-5) months who were visited home by AWW within 24 hours of last delivery
4	33	Proportion of mothers of infants (0-2/0-5) months who were visited home by any FLW within 24 hours of last delivery
4	34	Proportion of mothers of infants (0-2/0-5) months who were visited home by any AWW within the first week of last delivery

For peer review only

4	35	Proportion of mothers of infants (0-2/0-5) months who were visited home by any FLW within first week of last delivery
5	37	Proportion of mothers of infants (0-2/0-5) months whose last child was delivered at a health facility (private or public facility)
5	38	Proportion of mothers of infants (0-2/0-5) months whose last child was delivered at public facility
5	39	Proportion of mothers (home delivery) of infants (0-2/0-5) months who had home delivery attended by skilled birth attendant (SBA)
5	39.5	Proportion of mothers (home + institutional delivery) of infants (0-2/0-5) months who had home delivery attended by skill birth attendant (SBA)
6	42	Proportion of infants (home + institutional delivery) aged (0-2/0-5) months with nothing applied to the umbilical cord after cutting and tying in their last delivery
6	43	Proportion of mothers (home + institutional delivery) of infants (0-2/0-5) months who have delivered baby practiced skin to skin care (STSC) immediately after birth
6	44	Proportion of mothers with institutional delivery of infants (0-2/0-5) months who continued skin to skin care (STSC) at home
6	45	Proportion of mothers with home delivery of infants (0-2/0-5) months who continued skin to skin care (STSC) later
6	46	Proportion of mothers of infants (0-2/0-5) months who were breastfed within one hour of birth
6	47	Proportion of infants aged (0-2/0-5) months who have received delayed bath (between 48 hours and before 7 days after birth) (Public facility/Private facility/Home)
6	48	Proportion of infants aged (0-2/0-5) months who were weighed after birth (Public facility/Private facility/Home)
6	49	Proportion of infants aged (0-2/0-5) months who were delivered at HF received dry cord care
6	50	Proportion of infants aged (0-2/0-5) months who were delivered at HF continued with dry cord care
6	51	Proportion of infants aged (0-2/0-5) months who were delivered at home continued with dry cord care
7	52	Proportion of infants (0-2/0-5) months who were breast-fed in the past 24 hours (Exclusively Breast-fed)

a. 1 Antenatal care, 2 Maternal health, 3 Birth preparedness, 4 Front Line Worker Support, 6 Place of birth & attendant, 7 Exclusive Breastfeeding

For peer review only

Table S2. Indicators from two samples in Aurangabad and Gopalganj of mothers of infants aged 0-2 months compared to 0-5 months: Estimates, Cochran-Mantel-Haenszel Chi-squares, and probabilities: Bihar 2015

			Unweighted				Weighted			
Domain[a]	Indicator[b]	District[c]	Percent 0-2	Percent 0-5	Chi-square	Probability	Percent 0-2	Percent 0-5	Chi-square	Probability
1	1	1	86.6	79.9	3.6775	0.0552	85.2	77.6	4.3758	0.0365
1	1	2	85.3	85.3	0.0000	1.0000	85.0	85.4	0.0187	0.8911
1	2	1	44	40.7	0.4996	0.4797	43.0	38.3	1.0147	0.3138
1	2	2	47.7	47.0	0.0304	0.8616	47.5	48.2	0.0212	0.8843
1	3	1	84.7	80.9	1.2398	0.2655	83.8	80.4	0.9592	0.3274
1	3	2	81.2	78.6	0.5985	0.4391	80.6	77.8	0.6424	0.4229
1	4	1	60.3	63.6	0.4931	0.4826	61.1	63.1	0.1792	0.6720
1	4	2	59.4	63.2	0.8252	0.3637	57.4	61.0	0.7211	0.3958
1	5	1	42.1	45.0	0.3491	0.5546	43.2	45.7	0.2561	0.6128
1	5	2	44.7	47.7	0.4866	0.4854	43.4	46.9	0.6302	0.4273
1	6	1	59.8	59.3	0.0107	0.9177	58.9	60.1	0.0644	0.7996
1	6	2	67.7	63.5	1.0487	0.3058	66.3	63.3	0.5176	0.4719
1	7	1	42.6	39.2	0.5043	0.4776	42.0	39.5	0.2824	0.5952
1	7	2	70.3	73.3	0.6025	0.4376	69.2	72.6	0.7537	0.3853
1	8	1	44.0	45.9	0.1685	0.6815	42.7	44.8	0.2028	0.6525
1	8	2	53.4	54.1	0.0322	0.8576	52.4	54.5	0.2438	0.6215
1	9	1	45.9	42.6	0.5004	0.4793	45.8	41.7	0.7523	0.3857
1	9	2	42.9	43.6	0.0321	0.8579	42.8	43.5	0.0231	0.8792
1	10	1	67.0	59.8	2.6555	0.1032	67.2	59.6	2.9924	0.0837
1	10	2	55.3	58.3	0.5191	0.4712	54.8	58.5	0.7674	0.3810
1	40	1	11.5	17.7	3.4797	0.0621	10.6	16.0	2.8329	0.0924
1	40	2	8.6	10.2	0.3686	0.5438	8.5	9.5	0.1374	0.7109
2	11	1	98.6	98.1	0.1451	0.7033	98.5	98.1	0.0844	0.7715
2	11	2	97.0	96.6	0.0614	0.8044	96.9	97.1	0.0131	0.9089
2	12	1	68.4	66.5	0.1763	0.6746	69.3	67.5	0.1626	0.6868
2	12	2	79.7	79.3	0.0116	0.9141	81.9	80.4	0.2143	0.6434
2	13	1	3.8	2.4	0.7384	0.3902	4.0	2.6	0.6021	0.4378
2	13	2	3.4	5.6	1.5745	0.2096	3.0	5.1	1.6016	0.2057

For peer review only

Table S2. Indicators from mothers two samples in Aurangabad and Gopalganj of mothers of infants aged 0-2 months compared to 0-5 months: Estimates, Cochran-Mantel-Haenszel Chi-squares, and probabilities: Bihar 2015 - Continued

			Unweighted				Weighted			
Domain[a]	Indicator[b]	District[c]	Percent 0-2	Percent 0-5	Chi-square	Probability	Percent 0-2	Percent 0-5	Chi-square	Probability
2	14	1	1.4	1.9	0.1474	0.7010	1.6	2.0	0.1025	0.7489
2	14	2	1.9	3.8	1.7161	0.1902	1.7	3.6	1.8535	0.1734
3	15	1	26.3	34.0	3.1510	0.0759	25.9	33.3	2.9594	0.0854
3	15	2	47.0	56.4	4.9192	0.0266	45.7	56.0	5.8262	0.0158
3	16	1	4.3	3.8	0.0639	0.8004	4.3	3.6	0.1410	0.7073
3	16	2	10.2	13.9	1.8263	0.1766	9.7	14.3	2.8157	0.0933
3	17	1	48.8	56.5	2.5852	0.1079	48.5	55.6	2.2155	0.1366
3	17	2	53.4	62.8	4.9914	0.0255	51.8	62.6	6.5790	0.0103
3	18	1	24.9	22.5	0.3601	0.5485	26.1	23.4	0.4531	0.5009
3	18	2	39.5	45.1	2.2194	0.1363	38.2	43.7	2.1362	0.1439
3	19	1	22.6	14.4	3.4821	0.0620	23.5	14.5	4.0983	0.0429
3	19	2	45.2	40.7	1.1508	0.2834	43.4	39.4	0.6771	0.4106
3	20	1	40.7	37.8	0.4565	0.4993	44.1	39.5	1.1927	0.2748
3	20	2	51.9	57.1	1.8538	0.1733	50.0	55.9	2.3539	0.1250
3	21	1	40.1	30.4	3.6953	0.0546	43.6	31.2	6.0749	0.0137
3	21	2	58.1	52.8	1.7277	0.1887	55.6	51.6	0.8220	0.3646
3	22	1	65.1	59.3	1.5561	0.2122	65.5	59.4	1.7643	0.1841
3	22	2	62.4	68.4	2.2445	0.1341	60.2	68.0	3.6903	0.0547
3	23	1	65.0	49.0	8.3301	0.0039	62.5	47.0	7.8078	0.0052
3	23	2	68.8	63.0	1.8993	0.1682	68.0	62.5	1.5725	0.2098
4	24	1	61.2	75.1	9.2638	0.0023	62.2	75.2	8.2048	0.0042
4	24	2	65.8	74.4	4.8055	0.0284	63.5	73.0	5.6444	0.0175
4	25	1	11.5	12.9	0.2096	0.6471	11.1	12.5	0.2064	0.6496
4	25	2	20.7	18.4	0.4319	0.5111	19.3	19.1	0.0019	0.9656
4	26	1	62.7	76.6	9.4840	0.0021	63.5	76.7	8.6936	0.0032
4	26	2	68.4	75.9	3.8007	0.0512	66.9	74.3	3.5169	0.0607
4	27	1	42.1	47.8	1.4400	0.2301	42.3	47.6	1.2194	0.2695
4	27	2	54.5	62.4	3.4903	0.0617	52.6	61.1	4.0230	0.0449

For peer review only

Table S2. Indicators from mothers two samples in Aurangabad and Gopalganj of mothers of infants aged 0-2 months compared to 0-5 months: Estimates, Cochran-Mantel-Haenszel Chi-squares, and probabilities: Bihar 2015 - Continued

			Unweighted				Weighted			
Domain[a]	Indicator[b]	District[c]	Percent 0-2	Percent 0-5	Chi-square	Probability	Percent 0-2	Percent 0-5	Chi-square	Probability
4	28	1	8.6	8.1	0.0323	0.8575	8.2	7.6	0.0561	0.8127
4	28	2	16.2	13.9	0.5345	0.4647	15.4	13.8	0.2732	0.6012
4	29	1	43.1	48.8	1.4361	0.2308	43.3	48.5	1.1846	0.2764
4	29	2	57.5	64.7	2.9183	0.0876	55.9	63.5	3.2352	0.0721
4	30	1	29.7	35.4	1.6374	0.2007	28.7	34.2	1.5497	0.2132
4	30	2	48.1	53	1.2686	0.2600	46.9	51.4	1.0908	0.2963
4	31	1	30.1	45.9	10.9581	0.0000	29.9	44.9	9.9713	0.0016
4	31	2	47.4	53.0	1.6849	0.1943	44.5	52.7	3.6136	0.0573
4	32	1	5.3	3.8	0.4948	0.4818	5.3	3.6	0.7401	0.3896
4	32	2	9.8	9.0	0.0897	0.7646	9.1	9.9	0.0974	0.7550
4	33	1	32.5	47.8	10.1305	0.0015	32.2	46.7	9.0939	0.0026
4	33	2	50.8	56.4	1.7133	0.1906	48.2	56.2	3.5264	0.0604
4	34	1	6.2	10.0	2.0808	0.1492	6.2	9.9	1.9617	0.1613
4	34	2	13.5	9.0	2.7721	0.0959	14.4	8.9	3.9431	0.0471
4	35	1	45.0	60.3	9.7398	0.0018	44.5	59.3	9.0930	0.0026
4	35	2	64.7	69.5	1.4491	0.2287	62.5	69.0	2.5201	0.1124
5	37	1	73.2	76.6	0.6496	0.4203	72.9	74.9	0.2182	0.6404
5	37	2	80.1	86.1	3.4220	0.0643	78.9	85.5	3.9858	0.0459
5	38	1	55.5	56.5	0.0389	0.8436	54.7	55.3	0.0117	0.9139
5	38	2	52.3	61.3	4.3834	0.0363	51.2	61.6	5.8090	0.0159
5	39	1	5.7	0	3.3034	0.0691	5.4	0	3.6637	0.0556
5	39	2	10.2	8.3	0.2393	0.6247	9.1	6.6	0.3451	0.5569
5	39.5	1	1.4	0	0.0003	0.0833	1.4	0	2.8978	0.0887
5	39.5	2	1.9	1.1	0.5139	0.4735	1.8	0.9	0.7629	0.3824
6	42	1	42.6	47.4	1.0165	0.3134	43.8	47.6	0.6553	0.4182
6	42	2	41.4	46.2	1.3134	0.2518	41.8	46.3	1.1228	0.2893
6	43	1	24.9	29.7	1.3262	0.2495	25.6	30.4	1.3008	0.2541
6	43	2	27.1	29.7	0.4968	0.4809	26.9	31.2	1.2586	0.2619

For peer review only

Table S2. Indicators from mothers two samples in Aurangabad and Gopalganj of mothers of infants aged 0-2 months compared to 0-5 months: Estimates, Cochran-Mantel-Haenszel Chi-squares, and probabilities: Bihar 2015 - Continued

			Unweighted				Weighted			
Domain[a]	Indicator[b]	District[c]	Percent 0-2	Percent 0-5	Chi-square	Probability	Percent 0-2	Percent 0-5	Chi-square	Probability
6	44	1	9.8	12.5	1.2833	0.2573	12.2	13.0	0.3958	0.5293
6	44	2	21.1	20.1	0.0586	0.8087	21.6	18.7	0.5178	0.4718
6	45	1	7.5	10.2	0.6321	0.4266	7.8	11.1	0.8502	0.3565
6	45	2	8.2	5.6	0.4009	0.5266	5.2	8.3	0.1670	0.6828
6	46	1	55.5	58.9	0.4904	0.4837	56.6	59.2	0.2960	0.5864
6	46	2	62.8	68.0	1.6764	0.1954	61.7	66.3	1.2736	0.2591
6	47	1	34.0	32.1	0.1768	0.6741	35.9	32.8	0.4369	0.5086
6	47	2	67.3	66.5	0.0344	0.8528	66.6	68.3	0.1745	0.6762
6	48	1	65.6	67.5	0.1782	0.6729	64.9	66.6	0.1312	0.7172
6	48	2	71.1	77.8	3.2208	0.0727	70.7	78.2	3.9664	0.0464
6	49	1	62.7	59.4	0.1537	0.6950	64.5	61.2	0.1691	0.6809
6	49	2	68.1	67.2	0.0166	0.8974	67.3	66.8	0.0011	0.9735
6	50	1	49.0	55.0	1.2527	0.2630	48.4	54.7	1.3188	0.2508
6	50	2	56.8	55.9	0.0964	0.7562	56.1	55.5	0.0615	0.8042
6	51	1	81.1	44.9	14.9736	0.0000	78.0	45.4	11.7395	0.0000
6	51	2	63.3	38.9	4.0901	0.0431	63.7	41.1	3.4654	0.0627
7	52	1	70.3	59.8	5.1727	0.0229	69.2	59.7	4.1723	0.0411
7	52	2	82.3	67.3	16.2014	0.0000	82.1	68.4	13.3711	0.0000

a . 1 Antenatal care, 2 Maternal health, 3 Birth preparedness, 4 FLW Support, 5 Place of birth & attendant, 6 Neonatal Health, 7 Exclusive breastfeeding

b . For text see Table S1

c. 1 Aurangabad, 2 Gopalganj

For peer review only

Table S3. Weighted indicators and confidence intervals from two samples in two districts of Bihar

Indicator[a]	District[b]	0-2 Month Sample			0-5 Month Sample			Difference		
		Point estimate	Confidence interval[c]		Point estimate	Confidence interval[c]		Point estimate	Confidence interval	
			Lower	Upper		Lower	Upper		Lower	Upper
1	1	85.2	80.2	90.2	77.6	72.6	82.6	7.6	0.1	15.2
1	2	85.0	80.5	89.6	85.4	80.9	90.0	-0.4	-6.7	5.9
2	1	43.0	36.3	49.8	38.3	31.5	45.0	4.7	-4.5	14.0
2	2	47.5	41.5	53.6	48.2	42.1	54.2	-0.6	-9.5	8.2
3	1	83.8	78.8	88.8	80.4	75.4	85.4	3.4	-3.6	10.4
3	2	80.6	75.7	85.5	77.8	72.9	82.7	2.8	-4.5	10.0
4	1	61.1	54.4	67.8	63.1	56.4	69.9	-2.0	-11.4	7.3
4	2	57.4	51.2	63.6	61.0	54.8	67.2	-3.5	-12.3	5.2
5	1	43.2	36.4	50.1	45.7	38.9	52.6	-2.5	-12.2	7.2
5	2	43.4	37.1	49.8	46.9	40.5	53.2	-3.4	-12.4	5.6
6	1	58.9	52.3	65.6	60.1	53.5	66.7	-1.2	-10.4	8.0
6	2	66.3	60.2	72.4	63.3	57.2	69.4	2.9	-5.7	11.6
7	1	42.0	35.3	48.8	39.5	32.7	46.3	2.5	-7.0	12.1
7	2	69.2	63.2	75.2	72.6	66.6	78.6	-3.4	-11.7	4.9
8	1	42.7	36.1	49.3	44.8	38.2	51.4	-2.1	-11.4	7.2
8	2	52.4	46.2	58.7	54.5	48.2	60.8	-2.1	-10.9	6.7
9	1	45.8	39.0	52.6	41.7	34.9	48.4	4.1	-5.4	13.6
9	2	42.8	36.6	49.1	43.5	37.3	49.7	-0.6	-9.5	8.2
10	1	67.2	60.9	73.6	59.6	53.2	65.9	7.6	-1.2	16.5
10	2	54.8	48.5	61.1	58.5	52.2	64.8	-3.7	-12.5	5.2
11	1	98.5	96.7	100.2	98.1	96.4	99.9	0.4	-2.2	2.9
11	2	96.9	94.8	99.1	97.1	94.9	99.3	-0.2	-3.0	2.7
12	1	69.3	63.0	75.7	67.5	61.2	73.9	1.8	-7.2	10.8
12	2	81.9	77.3	86.6	80.4	75.7	85.0	1.6	-5.2	8.4
13	1	4.0	1.2	6.7	2.6	-0.1	5.4	1.3	-2.2	4.9
13	2	3.0	1.0	5.0	5.1	3.1	7.1	-2.2	-5.5	1.2
14	1	1.6	-0.2	3.4	2.0	0.2	3.8	-0.4	-3.1	2.3
14	2	1.7	0.2	3.2	3.6	2.1	5.1	-1.9	-4.7	0.9
15	1	25.9	20.1	31.6	33.3	27.5	39.0	-7.4	-15.9	1.1
15	2	45.7	39.6	51.8	56.0	49.9	62.1	-10.3	-19.0	-1.5
16	1	4.3	1.5	7.0	3.6	0.8	6.3	0.7	-3.0	4.4
16	2	9.7	6.1	13.3	14.3	10.8	17.9	-4.7	-10.5	1.1
17	1	48.5	41.8	55.2	55.6	48.9	62.3	-7.1	-16.5	2.4
17	2	51.8	45.6	58.0	62.6	56.4	68.8	-10.8	-19.6	-2.1
18	1	26.1	20.2	32.1	23.4	17.5	29.4	2.7	-5.5	10.9
18	2	38.2	33.0	43.3	43.7	38.5	48.8	-5.5	-13.2	2.2
19	1	23.5	16.4	30.7	14.5	7.3	21.7	9.1	0.4	17.7
19	2	43.4	37.3	49.5	39.4	33.3	45.5	4.0	-4.6	12.6
20	1	44.1	38.3	50.0	39.5	33.7	45.4	4.6	-3.7	12.9
20	2	50.0	44.7	55.4	55.9	50.5	61.3	-5.9	-13.7	2.0
21	1	43.6	36.7	50.5	31.2	24.3	38.1	12.4	3.3	21.5
21	2	55.6	49.5	61.7	51.6	45.5	57.7	4.0	-4.6	12.6
22	1	65.5	59.0	72.0	59.4	52.9	65.9	6.1	-3.0	15.2
22	2	60.2	54.0	66.3	68.0	61.8	74.2	-7.8	-16.4	0.7
23	1	62.5	54.9	70.2	47.0	39.3	54.7	15.5	5.4	25.7
23	2	68.0	61.2	74.8	62.5	55.7	69.3	5.5	-3.8	14.8

For peer review only

24	1	62.2	55.5	68.9	75.2	68.5	81.9	-13.0	-22.0	-4.1
24	2	63.5	57.4	69.7	73.0	66.9	79.2	-9.5	-17.9	-1.1
25	1	11.1	7.0	15.1	12.5	8.4	16.5	-1.4	-7.5	4.7
25	2	19.3	14.4	24.2	19.1	14.3	24.0	0.1	-6.9	7.2
26	1	63.5	56.8	70.1	76.7	70.1	83.4	-13.2	-22.1	-4.4
26	2	66.9	60.9	73.0	74.3	68.3	80.3	-7.4	-15.7	1.0
27	1	42.3	35.6	49.1	47.6	40.8	54.4	-5.3	-14.8	4.3
27	2	52.6	46.3	58.9	61.1	54.8	67.4	-8.5	-17.2	0.3
28	1	8.2	4.6	11.8	7.6	4.0	11.2	0.6	-4.4	5.7
28	2	15.4	11.0	19.8	13.8	9.4	18.3	1.6	-4.7	7.8
29	1	43.3	36.5	50.1	48.5	41.7	55.3	-5.2	-14.8	4.3
29	2	55.9	49.7	62.2	63.5	57.2	69.8	-7.5	-16.3	1.2
30	1	28.7	22.6	34.7	34.2	28.1	40.2	-5.5	-14.3	3.2
30	2	46.9	40.5	53.3	51.4	45.0	57.8	-4.5	-13.5	4.5
31	1	29.9	23.5	36.3	44.9	38.5	51.3	-15.0	-24.4	-5.6
31	2	44.5	38.2	50.8	52.7	46.5	59.0	-8.2	-17.2	0.7
32	1	5.3	2.2	8.5	3.6	0.4	6.7	1.7	-2.3	5.8
32	2	9.1	5.7	12.6	9.9	6.4	13.4	-0.8	-6.0	4.4
33	1	32.2	25.8	38.7	46.7	40.2	53.2	-14.5	-23.9	-5.0
33	2	48.2	41.9	54.5	56.2	49.9	62.5	-8.1	-16.9	0.8
34	1	6.2	2.8	9.6	9.9	6.5	13.3	-3.7	-8.9	1.5
34	2	14.4	9.8	19.0	8.9	4.4	13.5	5.5	-0.3	11.3
35	1	44.5	37.7	51.4	59.3	52.5	66.2	-14.8	-24.4	-5.1
35	2	62.5	56.4	68.7	69.0	62.9	75.2	-6.5	-15.1	2.1
37	1	72.9	66.7	79.1	74.9	68.7	81.0	-2.0	-10.5	6.5
37	2	78.9	73.5	84.2	85.5	80.2	90.8	-6.6	-13.7	0.4
38	1	54.7	47.8	61.7	55.3	48.4	62.2	-0.5	-10.3	9.2
38	2	51.2	44.7	57.6	61.6	55.2	68.0	-10.4	-19.4	-1.5
39	1	5.4	-0.9	11.8	0.0	-6.4	6.4	5.4	-0.9	11.8
39	2	9.1	1.3	16.9	6.6	-1.2	14.4	2.5	-9.1	14.1
39.5	1	1.4	-0.2	3.0	0.0	-1.6	1.6	1.4	-0.2	3.0
39.5	2	1.8	0.2	3.4	0.9	-0.6	2.5	0.9	-1.0	2.8
40	1	10.6	6.5	14.7	16.0	12.0	20.1	-5.4	-11.7	0.8
40	2	8.5	4.9	12.2	9.5	5.8	13.1	-0.9	-5.9	4.1
42	1	43.8	36.9	50.6	47.6	40.7	54.5	-3.8	-13.3	5.6
42	2	41.8	35.6	48.1	46.3	40.1	52.6	-4.5	-13.4	4.4
43	1	25.6	19.8	31.5	30.4	24.6	36.2	-4.8	-13.3	3.7
43	2	26.9	21.5	32.4	31.2	25.7	36.6	-4.2	-12.0	3.5
44	1	12.2	7.3	17.1	13.0	8.1	17.9	-0.8	-7.7	6.1
44	2	21.6	16.3	26.8	18.7	13.4	24.0	2.8	-4.4	10.1
45	1	7.8	0.4	15.2	11.1	3.7	18.5	-3.3	-16.2	9.6
45	2	5.2	0.3	10.1	8.3	3.4	13.2	-3.1	-17.4	11.2
46	1	56.6	49.9	63.2	59.2	52.5	65.8	-2.6	-12.2	7.0
46	2	61.7	55.5	67.9	66.3	60.1	72.6	-4.6	-13.3	4.1
47	1	35.9	29.3	42.5	32.8	26.2	39.5	3.0	-6.2	12.3
47	2	66.6	60.6	72.7	68.3	62.3	74.4	-1.7	-10.1	6.7
48	1	64.9	58.5	71.4	66.6	60.1	73.0	-1.6	-10.8	7.5
48	2	70.7	64.9	76.5	78.2	72.4	84.0	-7.5	-15.2	0.2
49	1	64.5	57.1	71.8	61.2	53.9	68.5	3.3	-6.7	13.3
49	2	67.3	60.6	73.9	66.8	60.1	73.4	0.5	-8.9	9.9
50	1	48.4	40.3	56.4	54.7	46.7	62.8	-6.4	-17.4	4.7
50	2	56.1	49.0	63.2	55.5	48.4	62.6	0.7	-9.2	10.5

For peer review only - http://bmjopen.bmj.com/site/about/guidelines.xhtml

51	1	78.0	65.9	90.2	45.4	33.3	57.6	32.6	13.0	52.2
51	2	63.7	48.8	78.5	41.1	26.2	56.0	22.5	-2.2	47.2
52	1	69.2	62.7	75.6	59.7	53.3	66.1	9.5	0.2	18.8
52	2	82.1	77.1	87.0	68.4	63.5	73.4	13.6	6.0	21.2

a. For text see Table S1
b. 1 Aurangabad, 2 Gopalganj
c. Estimated with Stata command svy

For peer review only

Table S4a: Point estimates and confidence intervals of indicators for subsamples of 0-2 and 3-5 month old infants in two districts: Bihar, India, 2015

Indicator[a]	District[b]	0-2 month subsample			3-5 month subsample			Difference		
		Point estimate	Confidence interval[c]		Point estimate	Confidence interval[c]		Point estimate	Confidence interval	
			Lower	Upper		Lower	Upper		Lower	Upper
1	1	79.2	71.9	86.5	75.2	66.0	84.4	4.0	-7.7	15.7
1	2	80.8	74.4	87.2	92.3	87.5	97.2	-11.5	-19.5	-3.5
2	1	36.1	27.8	44.4	41.5	31.7	51.3	-5.4	-18.2	7.4
2	2	46.5	38.1	54.8	50.6	40.3	61.0	-4.2	-17.5	9.1
3	1	81.0	75.0	87.1	79.5	71.5	87.5	1.5	-8.5	11.6
3	2	79.9	73.4	86.5	74.7	65.8	83.7	5.2	-5.9	16.3
4	1	61.8	53.1	70.4	65.2	55.0	75.3	-3.4	-16.7	10.0
4	2	62.7	54.7	70.7	58.4	48.4	68.3	4.4	-8.4	17.1
5	1	45.0	36.2	53.9	46.8	35.5	58.0	-1.7	-16.0	12.6
5	2	49.1	40.8	57.4	43.5	33.3	53.7	5.6	-7.6	18.7
6	1	60.3	51.9	68.7	59.8	49.6	70.0	0.5	-12.7	13.7
6	2	68.5	60.8	76.1	55.8	45.7	65.8	12.7	0.0	25.4
7	1	40.3	31.8	48.8	38.4	27.4	49.3	1.9	-11.9	15.8
7	2	74.5	67.2	81.9	69.8	60.3	79.3	4.7	-7.3	16.8
8	1	49.3	41.2	57.3	38.2	27.9	48.5	11.1	-2.0	24.2
8	2	60.0	52.0	67.9	46.4	36.7	56.2	13.6	1.0	26.1
9	1	39.8	31.3	48.4	44.4	33.9	54.8	-4.5	-18.0	9.0
9	2	44.7	36.6	52.8	41.7	31.5	51.9	3.0	-10.0	16.0
10	1	61.9	54.4	69.4	56.1	45.3	66.9	5.7	-7.4	18.9
10	2	63.9	56.1	71.7	50.5	40.3	60.6	13.5	0.7	26.2
11	1	98.4	96.3	100.6	97.6	94.3	100.9	0.8	-3.1	4.8
11	2	96.7	94.1	99.3	97.7	95.1	100.3	-1.0	-4.7	2.7
12	1	65.0	56.4	73.6	71.3	61.8	80.7	-6.3	-19.1	6.5
12	2	77.8	71.1	84.6	84.1	76.7	91.4	-6.2	-16.2	3.8
13	1	3.3	0.1	6.5	1.6	-1.5	4.8	1.7	-2.8	6.2
13	2	6.7	2.6	10.9	2.8	0.0	5.6	3.9	-1.1	8.9
14	1	2.2	-0.4	4.8	1.6	-1.5	4.8	0.6	-3.5	4.7
14	2	4.5	1.0	8.0	2.3	-0.4	4.9	2.2	-2.2	6.6
15	1	35.2	26.8	43.6	30.4	20.9	39.9	4.8	-7.8	17.5
15	2	56.8	48.7	64.9	54.8	44.3	65.3	2.0	-11.3	15.2
16	1	5.3	1.4	9.3	0.9	-0.8	2.6	4.5	0.2	8.7
16	2	16.6	10.2	23.0	11.0	4.8	17.1	5.7	-3.2	14.5
17	1	59.6	51.4	67.9	49.5	38.7	60.3	10.1	-3.5	23.7
17	2	64.4	56.5	72.4	59.9	49.8	70.0	4.5	-8.3	17.4
18	1	24.2	16.8	31.6	22.2	13.9	30.5	2.0	-9.1	13.1
18	2	45.9	38.1	53.8	40.3	32.0	48.6	5.7	-5.7	17.1
19	1	17.9	11.2	24.5	9.2	2.4	16.1	8.6	-0.9	18.2
19	2	40.5	32.4	48.6	37.7	29.0	46.4	2.8	-9.0	14.6
20	1	42.5	34.8	50.2	35.1	26.0	44.2	7.4	-4.5	19.4
20	2	59.4	51.7	67.0	50.7	42.3	59.1	8.6	-2.7	20.0
21	1	37.0	29.2	44.8	22.1	13.1	31.2	14.9	2.9	26.8
21	2	54.2	46.1	62.3	47.7	38.9	56.6	6.5	-5.6	18.5
22	1	62.5	54.0	70.9	54.7	44.8	64.6	7.8	-5.2	20.8
22	2	69.7	62.3	77.1	65.5	55.4	75.5	4.2	-8.2	16.7
23	1	47.5	39.1	55.9	46.1	35.0	57.3	1.4	-12.5	15.3

23	2	64.9	56.9	73.0	58.9	48.4	69.5	6.0	-7.3	19.3
24	1	75.1	67.4	82.9	75.3	66.0	84.7	-0.2	-12.4	11.9
24	2	67.6	59.7	75.5	81.0	72.7	89.3	-13.4	-24.8	-1.9
25	1	12.1	6.1	18.0	13.1	5.9	20.2	-1.0	-10.3	8.3
25	2	16.7	10.3	23.1	22.7	13.8	31.6	-6.0	-16.9	4.9
26	1	76.2	68.6	83.8	77.5	68.5	86.6	-1.3	-13.1	10.5
26	2	69.4	61.6	77.2	81.6	73.3	89.8	-12.2	-23.6	-0.8
27	1	50.4	41.5	59.3	43.4	33.0	53.9	6.9	-6.8	20.7
27	2	57.2	48.9	65.4	66.9	57.7	76.0	-9.7	-22.0	2.7
28	1	8.6	3.7	13.6	6.0	1.3	10.6	2.7	-4.1	9.5
28	2	9.8	5.1	14.5	19.7	11.4	28.1	-9.9	-19.5	-0.3
29	1	50.4	41.5	59.3	45.6	35.4	55.9	4.8	-8.8	18.4
29	2	59.2	51.1	67.4	69.7	60.5	79.0	-10.5	-22.9	1.8
30	1	32.7	24.5	41.0	36.3	25.9	46.7	-3.6	-16.9	9.7
30	2	49.2	41.0	57.4	54.7	44.3	65.1	-5.5	-18.8	7.7
31	1	42.0	33.0	51.1	49.2	39.0	59.5	-7.2	-20.9	6.5
31	2	48.6	40.3	56.8	58.9	48.8	69.0	-10.3	-23.3	2.7
32	1	3.9	0.4	7.3	3.2	-0.4	6.8	0.7	-4.3	5.7
32	2	9.4	4.2	14.5	10.7	4.7	16.8	-1.4	-9.3	6.5
33	1	43.6	34.5	52.6	51.4	41.1	61.7	-7.8	-21.6	5.9
33	2	52.9	44.8	61.0	61.1	51.1	71.2	-8.2	-21.1	4.7
34	1	7.0	2.5	11.5	14.2	7.0	21.4	-7.2	-15.7	1.2
34	2	8.6	3.8	13.4	9.5	4.0	15.0	-0.9	-8.2	6.4
35	1	57.2	48.2	66.2	62.5	52.7	72.4	-5.3	-18.7	8.0
35	2	63.2	55.1	71.4	77.6	68.6	86.6	-14.3	-26.5	-2.2
37	1	76.6	69.1	84.1	72.3	62.7	81.8	4.3	-7.8	16.4
37	2	81.6	74.9	88.3	91.3	85.8	96.8	-9.7	-18.4	-1.0
38	1	59.0	49.9	68.0	49.7	39.3	60.2	9.2	-4.6	23.1
38	2	56.1	47.8	64.4	69.7	61.2	78.2	-13.6	-25.5	-1.7
39	1	0.0	0.0	0.0	0.0	0.0	0.0	0.0	0.0	0.0
39	2	5.8	-3.1	14.7	9.6	-40.1	59.3	-3.8	-54.3	46.7
39.5	1	0.0	0.0	0.0	0.0	0.0	0.0	0.0	0.0	0.0
39.5	2	1.1	-0.4	2.6	0.7	-0.7	2.1	0.3	-1.7	2.4
40	1	17.6	10.9	24.4	13.6	7.5	19.8	4.0	-5.2	13.2
40	2	8.0	3.9	12.1	11.6	5.6	17.7	-3.7	-11.0	3.6
42	1	52.6	44.3	61.0	40.1	29.6	50.6	12.5	-0.9	25.9
42	2	47.5	39.4	55.6	44.6	34.4	54.8	2.9	-10.2	16.0
43	1	35.6	27.4	43.8	22.6	13.6	31.5	13.1	0.9	25.2
43	2	35.5	28.0	43.0	24.8	16.4	33.1	10.8	-0.5	22.0
44	1	10.1	4.2	16.0	17.6	9.5	25.7	-7.5	-17.6	2.5
44	2	20.6	14.1	27.0	16.2	8.7	23.8	4.3	-5.6	14.3
45	1	9.0	-4.7	22.7	13.7	-0.7	28.1	-4.7	-24.6	15.2
45	2	6.6	-9.9	23.2	14.4	14.4	14.4	-7.7	-24.3	8.8
46	1	63.4	54.6	72.2	52.8	41.5	64.0	10.7	-3.6	24.9
46	2	68.9	61.1	76.7	62.5	52.6	72.4	6.4	-6.2	19.0
47	1	33.0	24.7	41.4	32.5	22.2	42.9	0.5	-12.8	13.8
47	2	70.4	62.8	77.9	65.3	55.9	74.8	5.1	-7.1	17.2
48	1	68.8	60.6	77.0	63.2	52.5	73.8	5.7	-7.8	19.1
48	2	76.2	69.3	83.0	81.3	73.9	88.6	-5.1	-15.2	5.0
49	1	66.5	57.9	75.1	52.7	41.0	64.3	13.8	-0.6	28.3
49	2	68.6	59.9	77.3	64.4	53.8	75.0	4.2	-9.5	17.9
50	1	51.7	42.1	61.4	59.5	47.6	71.5	-7.8	-23.1	7.6

For peer review only

50	2	55.2	46.3	64.1	55.8	45.1	66.6	-0.7	-14.6	13.3
51	1	33.2	14.6	51.7	61.0	36.7	85.4	-27.9	-58.5	2.7
51	2	40.6	17.5	63.7	43.0	-6.7	92.7	-2.4	-57.2	52.4
52	1	73.0	65.1	80.9	39.7	28.6	50.7	33.3	19.8	46.9
52	2	83.8	78.2	89.3	45.8	35.6	55.9	38.0	26.4	49.5

a. For text see Table S1
b. 1 Aurangabad, 2 Gopalganj
c. Estimated with Stata command svy

For peer review only

Table S4b: Indicators in Table S4a where zero is not in the estimated confidence interval of difference between 0-2 and 3-5 month subsamples

	Indicator	District[a]	Point estimate 0-2 month	Point estimate 3-5 month	Difference	Confidence interval[b] Lower	Confidence interval[b] Upper
			In Table S4a and in Table S2				
21	Proportion of mothers who planned institutional delivery of infants (0-2/0-5) months who arranged clean cloth for mothers and baby	1	37.0	22.1	14.9	2.9	26.8
24	Proportion of mothers of infants (0-2/0-5) months who were visited by ASHA at least once during their last pregnancy	2	67.6	81.0	-13.4	-24.8	-1.9
37	Proportion of mothers of infants (0-2/0-5) months whose last child was delivered at a health facility (private or public facility)	2	81.6	91.3	-9.7	-18.4	-1.0
38	Proportion of mothers of infants (0-2/0-5) months whose last child was delivered at public facility	2	56.1	69.7	-13.6	-25.5	-1.7
52	Proportion of infants (0-2/0-5) months who were breast-fed in the past	1	73.0	39.7	33.3	19.8	46.9
52	24 hours (Exclusively Breast-fed)	2	83.8	45.8	38.0	26.4	49.5
			In Table S4a but not in Table S2				
1	Proportion of mothers of infants (0-2/0-5) months who were registered during their last pregnancy	2	80.8	92.3	-11.5	-19.5	-3.5
8	Proportion of mothers of infants (0-2/0-5) months who attended at least one ANC where at least one abdominal examination was performed during her last pregnancy	2	60.0	46.4	13.6	1.0	26.1
10	Proportion of mothers of infants (0-2/0-5) months who attended at least one ANC where at least one blood test was performed during her last pregnancy	2	63.9	50.5	13.5	0.7	26.2
16	Proportion of mothers (home + institutional delivery) of infants (0-2/0-5) months who have identified anybody who would donate blood in the case of emergency in their last pregnancy	1	5.3	0.9	4.5	0.2	8.7
26	Proportion of mothers of infants (0-2/0-5) months who were visited by FLWs at least once during their last pregnancy	2	69.4	81.6	-12.2	-23.6	-0.8
28	Proportion of mothers of infants (0-2/0-5) months who were visited by AWW in the last trimester during their last pregnancy	2	9.8	19.7	-9.9	-19.5	-0.3
35	Proportion of mothers of infants (0-2/0-5) months who were visited home by any FLW within first week of last delivery	2	63.2	77.6	-14.3	-26.5	-2.2

For peer review only

43	Proportion of mothers (home + institutional delivery) of infants (0-2/0-5) months who have delivered baby practiced skin to skin care (STSC) immediately after birth	1	35.6	22.6	13.1	0.9	25.2

a. 1 = Aurangabad; 2 = Gopalganj
b. Calculated from standard errors for point estimates estimated with Stata command svy

For peer review only

Table S5a: Point estimates and confidence intervals of indicators for a sample 0-2 month old infants and a subsample of 0-2 month old infants from a sample of 0-5 month old infants in two districts: Bihar, India, 2015

Indicator[a]	District[b]	0-2 month subsample			0-2 month sample			Difference		
		Point estimate	Confidence interval[c]		Point estimate	Confidence interval[c]		Point estimate	Confidence interval	
			Lower	Upper		Lower	Upper		Lower	Upper
1	1	79.2	71.9	86.5	85.2	80.2	90.2	-6.0	-14.8	2.8
1	2	80.8	74.4	87.2	85.0	80.5	89.6	-4.2	-12.1	3.6
2	1	36.1	27.8	44.4	43.0	36.3	49.8	-6.9	-17.6	3.8
2	2	46.5	38.1	54.8	47.5	41.5	53.6	-1.1	-11.3	9.2
3	1	81.0	75.0	87.1	83.8	78.8	88.8	-2.8	-10.7	5.1
3	2	79.9	73.4	86.5	80.6	75.7	85.5	-0.7	-8.8	7.5
4	1	61.8	53.1	70.4	61.1	54.4	67.8	0.7	-10.3	11.6
4	2	62.7	54.7	70.7	57.4	51.2	63.6	5.3	-4.8	15.4
5	1	45.0	36.2	53.9	43.2	36.4	50.1	1.8	-9.4	13.0
5	2	49.1	40.8	57.4	43.4	37.1	49.8	5.7	-4.8	16.2
6	1	60.3	51.9	68.7	58.9	52.3	65.6	1.4	-9.3	12.1
6	2	68.5	60.8	76.1	66.3	60.2	72.4	2.2	-7.6	12.0
7	1	40.3	31.8	48.8	42.0	35.3	48.8	-1.7	-12.6	9.2
7	2	74.5	67.2	81.9	69.2	63.2	75.2	5.3	-4.2	14.8
8	1	49.3	41.2	57.3	42.7	36.1	49.3	6.5	-3.9	16.9
8	2	60.0	52.0	67.9	52.4	46.2	58.7	7.6	-2.6	17.7
9	1	39.8	31.3	48.4	45.8	39.0	52.6	-5.9	-16.8	5.0
9	2	44.7	36.6	52.8	42.8	36.6	49.1	1.8	-8.3	12.0
10	1	61.9	54.4	69.4	67.2	60.9	73.6	-5.3	-15.2	4.5
10	2	63.9	56.1	71.7	54.8	48.5	61.1	9.1	-0.9	19.1
11	1	98.4	96.3	100.6	98.5	96.7	100.2	-0.0	-2.8	2.8
11	2	96.7	94.1	99.3	96.9	94.8	99.1	-0.2	-3.7	3.2
12	1	65.0	56.4	73.6	69.3	63.0	75.7	-4.3	-15.0	6.4
12	2	77.8	71.1	84.6	81.9	77.3	86.6	-4.1	-12.3	4.1
13	1	3.3	0.1	6.5	4.0	1.2	6.7	-0.6	-4.9	3.6
13	2	6.7	2.6	10.9	3.0	1.0	5.0	3.7	-0.9	8.3
14	1	2.2	-0.4	4.8	1.6	-0.2	3.4	0.7	-2.5	3.8
14	2	4.5	1.0	8.0	1.7	0.2	3.2	2.8	-1.1	6.7
15	1	35.2	26.8	43.6	25.9	20.1	31.6	9.3	-0.8	19.5
15	2	56.8	48.7	64.9	45.7	39.6	51.8	11.1	0.9	21.2
16	1	5.3	1.4	9.3	4.3	1.5	7.0	1.1	-3.7	5.9
16	2	16.6	10.2	23.0	9.7	6.1	13.3	7.0	-0.4	14.3
17	1	59.6	51.4	67.9	48.5	41.8	55.2	11.1	0.5	21.7
17	2	64.4	56.5	72.4	51.8	45.6	58.0	12.7	2.6	22.7
18	1	24.2	16.8	31.6	26.1	20.2	32.1	-1.9	-11.4	7.6
18	2	45.9	38.1	53.8	38.2	33.0	43.3	7.8	-1.6	17.1
19	1	17.9	11.2	24.5	23.5	16.4	30.7	-5.7	-15.5	4.1
19	2	40.5	32.4	48.6	43.4	37.3	49.5	-2.9	-13.0	7.3
20	1	42.5	34.8	50.2	44.1	38.3	50.0	-1.6	-11.3	8.0
20	2	59.4	51.7	67.0	50.0	44.7	55.4	9.3	0.0	18.7
21	1	37.0	29.2	44.8	43.6	36.7	50.5	-6.6	-17.0	3.8
21	2	54.2	46.1	62.3	55.6	49.5	61.7	-1.4	-11.6	8.7
22	1	62.5	54.0	70.9	65.5	59.0	72.0	-3.0	-13.7	7.7
22	2	69.7	62.3	77.1	60.2	54.0	66.3	9.5	-0.1	19.2

For peer review only

23	1	47.5	39.1	55.9	62.5	54.9	70.2	-15.0	-26.4	-3.6
23	2	64.9	56.9	73.0	68.0	61.2	74.8	-3.1	-13.7	7.4
24	1	75.1	67.4	82.9	62.2	55.5	68.9	12.9	2.7	23.2
24	2	67.6	59.7	75.5	63.5	57.4	69.7	4.1	-5.9	14.1
25	1	12.1	6.1	18.0	11.1	7.0	15.1	1.0	-6.2	8.2
25	2	16.7	10.3	23.1	19.3	14.4	24.2	-2.6	-10.6	5.5
26	1	76.2	68.6	83.8	63.5	56.8	70.1	12.7	2.6	22.8
26	2	69.4	61.6	77.2	66.9	60.9	73.0	2.4	-7.4	12.3
27	1	50.4	41.5	59.3	42.3	35.6	49.1	8.1	-3.1	19.3
27	2	57.2	48.9	65.4	52.6	46.3	58.9	4.6	-5.8	15.0
28	1	8.6	3.7	13.6	8.2	4.6	11.8	0.5	-5.7	6.6
28	2	9.8	5.1	14.5	15.4	11.0	19.8	-5.6	-12.0	0.8
29	1	50.4	41.5	59.3	43.3	36.5	50.1	7.1	-4.1	18.3
29	2	59.2	51.1	67.4	55.9	49.7	62.2	3.3	-7.0	13.6
30	1	32.7	24.5	41.0	28.7	22.6	34.7	4.1	-6.1	14.3
30	2	49.2	41.0	57.4	46.9	40.5	53.3	2.3	-8.1	12.7
31	1	42.0	33.0	51.1	29.9	23.5	36.3	12.1	1.1	23.2
31	2	48.6	40.3	56.8	44.5	38.2	50.8	4.1	-6.3	14.5
32	1	3.9	0.4	7.3	5.3	2.2	8.5	-1.5	-6.2	3.2
32	2	9.4	4.2	14.5	9.1	5.7	12.6	0.2	-6.0	6.4
33	1	43.6	34.5	52.6	32.2	25.8	38.7	11.3	0.2	22.5
33	2	52.9	44.8	61.0	48.2	41.9	54.5	4.7	-5.5	15.0
34	1	7.0	2.5	11.5	6.2	2.8	9.6	0.8	-4.8	6.4
34	2	8.6	3.8	13.4	14.4	9.8	19.0	-5.8	-12.5	0.8
35	1	57.2	48.2	66.2	44.5	37.7	51.4	12.7	1.3	24.0
35	2	63.2	55.1	71.4	62.5	56.4	68.7	0.7	-9.5	10.9
37	1	76.6	69.1	84.1	72.9	66.7	79.1	3.7	-6.0	13.4
37	2	81.6	74.9	88.3	78.9	73.5	84.2	2.7	-5.8	11.3
38	1	59.0	49.9	68.0	54.7	47.8	61.7	4.2	-7.2	15.6
38	2	56.1	47.8	64.4	51.2	44.7	57.6	4.9	-5.6	15.4
39	1	0.0	0.0	0.0	5.4	-0.9	11.8	-5.4	-11.8	0.9
39	2	5.8	-3.1	14.7	9.1	1.3	16.9	-3.3	-15.1	8.5
40	1	0.0	0.0	0.0	1.4	-0.2	3.0	-1.4	-3.0	0.2
40	2	1.1	-0.4	2.6	1.8	0.2	3.4	-0.7	-2.9	1.4
40	1	17.6	10.9	24.4	10.6	6.5	14.7	7.0	-0.9	14.9
40	2	8.0	3.9	12.1	8.5	4.9	12.2	-0.6	-6.0	4.9
42	1	52.6	44.3	61.0	43.8	36.9	50.6	8.9	-2.0	19.7
42	2	47.5	39.4	55.6	41.8	35.6	48.1	5.7	-4.6	15.9
43	1	35.6	27.4	43.8	25.6	19.8	31.5	10.0	-0.1	20.1
43	2	35.5	28.0	43.0	26.9	21.5	32.4	8.6	-0.7	17.8
44	1	10.1	4.2	16.0	12.2	7.3	17.1	-2.1	-9.8	5.6
44	2	20.6	14.1	27.0	21.6	16.3	26.8	-1.0	-9.3	7.3
45	1	9.0	-4.7	22.7	7.8	0.4	15.2	1.2	-14.3	16.8
45	2	6.6	-9.9	23.2	5.2	0.3	10.1	1.4	-15.8	18.7
46	1	63.4	54.6	72.2	56.6	49.9	63.2	6.9	-4.2	17.9
46	2	68.9	61.1	76.7	61.7	55.5	67.9	7.2	-2.8	17.2
47	1	33.0	24.7	41.4	35.9	29.3	42.5	-2.8	-13.5	7.8
47	2	70.4	62.8	77.9	66.6	60.6	72.7	3.7	-6.0	13.4
48	1	68.8	60.6	77.0	64.9	58.5	71.4	3.9	-6.5	14.3
48	2	76.2	69.3	83.0	70.7	64.9	76.5	5.4	-3.6	14.4
49	1	66.5	57.9	75.1	64.5	57.1	71.8	2.1	-9.2	13.3
49	2	68.6	59.9	77.3	67.3	60.6	73.9	1.3	-9.7	12.3

For peer review only

50	1	51.7	42.1	61.4	48.4	40.3	56.4	3.4	-9.2	15.9
50	2	55.2	46.3	64.1	56.1	49.0	63.2	-0.9	-12.3	10.4
51	1	33.2	14.6	51.7	78.0	65.9	90.2	-44.9	-67.0	-22.7
51	2	40.6	17.5	63.7	63.7	48.8	78.5	-23.0	-50.5	4.5
52	1	73.0	65.1	80.9	69.2	62.7	75.6	3.8	-6.3	14.0
52	2	83.8	78.2	89.3	82.1	77.1	87.0	1.7	-5.7	9.1

a. For text see Table S1
b. 1 Aurangabad, 2 Gopalganj
c. Estimated with Stata command svy

For peer review only

Table S5b: Point estimates of weighted indicators and differences between a sample of mothers of 0-2 month old infants and a subsample of mothers of 0-2 month old infants from a sample of mothers of 0-5 month old infants in two districts of Bihar, India

	Indicator	District[a]	Point estimates		Difference		
					Estimate	Confidence interval[b]	
			Subsample	Sample		Lower	Upper
15	Proportion of mothers (home + institutional delivery) of infants (0-2/0-5) months who planned transportation to health facility in their last pregnancy	2	56.8	45.7	11.1	0.9	21.2
17	Proportion of mothers (home + institutional delivery) of infants (0-2/0-5) months who have identified persons who would take care of the baby immediately after birth	1	59.6	48.5	11.1	0.5	21.7
		2	64.4	51.8	12.7	2.6	22.7
23	Proportion of mothers who planned for institutional delivery of infants (0-2/0-5) months identified person to accompany her during the delivery	1	47.5	62.5	-15.0	-26.4	-3.6
24	Proportion of mothers of infants (0-2/0-5) months who were visited by ASHA at least once during their last pregnancy	1	75.1	62.2	12.9	2.7	23.2
26	Proportion of mothers of infants (0-2/0-5) months who were visited by FLWs at least once during their last pregnancy	1	76.2	63.5	12.7	2.6	22.8
31	Proportion of mothers of infants (0-2/0-5) months who were visited home by ASHA within 24 hours of last delivery	1	42.0	29.9	12.1	1.1	23.2
33	Proportion of mothers of infants (0-2/0-5) months who were visited home by any FLW within 24 hours of last delivery	1	43.6	32.2	11.3	0.2	22.5
35	Proportion of mothers of infants (0-2/0-5) months who were visited home by any FLW within first week of last delivery	1	57.2	44.5	12.7	1.3	24.0
51	Proportion of infants aged (0-2/0-5) months who were delivered at home continued with dry cord care	1	33.2	78.0	-44.9	-67.0	-22.7

a. 1 = Aurangabad; 2 = Gopalganj
b. Calculated from standard errors for point estimates estimated with Stata command svy

For peer review only

Table S6: Point estimates and confidence intervals of indicators for a sample of 3-5 month infants and subsample of 3-5 month infants in two districts: Bihar, India, 2015

Indicator[a]	District[b]	3-5 month subsample			3-5 month sample			Difference		
		Point estimate	Confidence interval[c]		Point estimate	Confidence interval[c]		Point estimate	Confidence interval	
			Lower	Upper		Lower	Upper		Lower	Upper
37	1	72.3	62.7	81.8	70.4	64.2	76.5	1.9	13.3	-9.5
37	2	91.3	85.8	96.8	86.2	81.8	90.6	5.1	12.2	-2.0
38	1	49.7	39.3	60.2	45.8	38.9	52.6	4.0	16.4	-8.5
38	2	69.7	61.2	78.2	64.4	58.3	70.5	5.4	15.8	-5.1
52	1	39.7	28.6	50.7	65.3	58.8	71.8	-25.6	-12.8	-38.4
52	2	45.8	35.6	55.9	55.2	49.1	61.2	-9.4	2.4	-21.2

a. For text see Table S1
b. 1 Aurangabad, 2 Gopalganj
c. Estimated with Stata command svy

For peer review only
